# Hemodynamic Changes During Physiological and Pharmacological Stress Testing in Patients With Heart Failure: A Systematic Review and Meta-Analysis

**DOI:** 10.3389/fcvm.2022.718114

**Published:** 2022-04-19

**Authors:** Anne Bingel, Daniel Messroghli, Andreas Weimar, Kilian Runte, Maximilian Salcher-Konrad, Sebastian Kelle, Burkert Pieske, Felix Berger, Titus Kuehne, Leonid Goubergrits, Daniel Fuerstenau, Marcus Kelm

**Affiliations:** ^1^Department of Internal Medicine and Cardiology, German Heart Center Berlin, Berlin, Germany; ^2^Department of Internal Medicine/Cardiology, Charité—Universitätsmedizin Berlin, Berlin, Germany; ^3^German Center for Cardiovascular Research (DZHK), Partner Site Berlin, Berlin, Germany; ^4^Corporate Member of Freie Universität Berlin and Humboldt-Universität zu Berlin, Institute of Medical Informatics, Charité – Universitätsmedizin Berlin, Berlin, Germany; ^5^Department of Congenital Heart Disease, German Heart Center Berlin, Berlin, Germany; ^6^Care Policy and Evaluation Centre, London School of Economics and Political Science, London, United Kingdom; ^7^Institute for Imaging Science and Computational Modelling in Cardiovascular Medicine, Charité—Universitätsmedizin Berlin, Berlin, Germany; ^8^Einstein Center Digital Future (ECDF), Berlin, Germany; ^9^Department of Digitalization, Copenhagen Business School, Frederiksberg, Denmark; ^10^Berlin Institute of Health (BIH), Berlin, Germany

**Keywords:** heart failure, stress testing, meta-analysis, HFpEF—heart failure with preserved ejection fraction, HFrEF—heart failure with reduced ejection fraction, physiologic changes, exercise testing (in) heart failure

## Abstract

Although disease etiologies differ, heart failure patients with preserved and reduced ejection fraction (HFpEF and HFrEF, respectively) both present with clinical symptoms when under stress and impaired exercise capacity. The extent to which the adaptation of heart rate (HR), stroke volume (SV), and cardiac output (CO) under stress conditions is altered can be quantified by stress testing in conjunction with imaging methods and may help to detect the diminishment in a patient’s condition early. The aim of this meta-analysis was to quantify hemodynamic changes during physiological and pharmacological stress testing in patients with HF. A systematic literature search (PROSPERO 2020:CRD42020161212) in MEDLINE was conducted to assess hemodynamic changes under dynamic and pharmacological stress testing at different stress intensities in HFpEF and HFrEF patients. Pooled mean changes were estimated using a random effects model. Altogether, 140 study arms with 7,248 exercise tests were analyzed. High-intensity dynamic stress testing represented 73% of these data (70 study arms with 5,318 exercise tests), where: HR increased by 45.69 bpm (95% CI 44.51–46.88; *I*^2^ = 98.4%), SV by 13.49 ml (95% CI 6.87–20.10; *I*^2^ = 68.5%), and CO by 3.41 L/min (95% CI 2.86–3.95; *I*^2^ = 86.3%). No significant differences between HFrEF and HFpEF groups were found. Despite the limited availability of comparative studies, these reference values can help to estimate the expected hemodynamic responses in patients with HF. No differences in chronotropic reactions, changes in SV, or CO were found between HFrEF and HFpEF. When compared to healthy individuals, exercise tolerance, as well as associated HR and CO changes under moderate-high dynamic stress, was substantially impaired in both HF groups. This may contribute to a better disease understanding, future study planning, and patient-specific predictive models.

**Systematic Review Registration:**

[https://www.crd.york.ac.uk/prospero/], identifier [CRD42020161212].

## Introduction

Mortality rates and the frequency of symptom deterioration requiring hospitalization are nearly identical between heart failure (HF) with reduced (HFrEF) and preserved ejection fraction (HFpEF) (1). Both tend to have an impaired exercise capacity causing symptoms with exertion. An impaired contractile reserve and left ventricular remodelng are the key characteristics of HFrEF (2). The origins of HFrEF comprise a broad spectrum of etiologies, of which hypertension and ischemic heart disease are the leading causes (3). Almost half of all patients suffering from HF have a preserved ejection fraction (HFpEF) (4). These individuals are typically older, more often female, diabetic, and obese, and more frequently present with renal disease and arterial hypertension combined with left ventricular hypertrophy (5, 6). HFpEF is characterized by an impaired diastolic function accompanied by vascular changes resulting in an abnormal ventricular-arterial coupling (7). As poor functional capacity reduces the quality of life and indicates a worse prognosis in both groups of HF (8), the objective quantification of exercise intolerance is of importance, especially when symptoms occur (9).

The extent to which reduced cardiac output (CO) limits exercise tolerance can be quantified by different forms of cardiac stress testing, using dynamic and pharmacological, as well as isometric stress. Combining these tools with imaging methods, such as cardiac magnetic resonance imaging (MRI) or echocardiography, allows for the analysis of hemodynamic parameters. While the diagnosis of HFrEF is unequivocal, identifying patients with HFpEF can be more challenging, especially when patients present in a stable condition, so that the diagnosis mainly relies on imaging parameters indirectly indicating elevated left ventricular filling pressures (10, 11). Computational models simulating physiological or non-physiological responses to stress have, therefore, become of interest in achieving a better understanding of both cardiovascular hemodynamic interactions and early detection (12). To develop and optimize such predictions in patients with HF, reliable and robust disease-specific reference data of hemodynamic responses are required.

We performed, therefore, a systematic analysis of the available literature that has assessed hemodynamic changes under stress testing in patients with HF. In addition to providing reference ranges for the expected changes during exercise testing, we explored the associations of these stress-induced changes to cardiovascular parameters at rest, as well as medical therapy profiles of the included studies.

## Materials and Methods

### Search Strategy

A pre-established review protocol was used and registered in PROSPERO (CRD42020161212). The specific search included studies in which patients with HF performed dynamic, isometric, or pharmacological stress testing and where hemodynamic changes were assessed by MRI, ECG, or echocardiography. The search aspects are specified in the standardized scheme addressing patient population, interventions, comparators, outcomes, and study design (PICOS) in Table 1. Prior to our analysis, no meta-analysis has addressed this question in the HF patient population. Nevertheless, the study was built on previous study addressing this question in healthy controls (13). We conducted our search in MEDLINE (*via* PubMed) deploying pre-specified search items (Supplementary Table 1). No relevant deviations were found compared to an Embase query. The date of the final search was 29 February 2020.

**TABLE 1:** The population, interventions, comparators, outcomes, and study design (PICOS) scheme.

PICOS	
Patient population	HFrEF patients undergoing stress testing combined with MRI, ECG, or echocardiography HFpEF patients undergoing stress testing combined with MRI, ECG, or echocardiography
Interventions	Dynamic exercise Dobutamine infusion Isometric exercise
Comparators	Resting state
Outcomes	Heart rate [bpm] Stroke volume [ml] Cardiac output [L/min] Ejection fraction [%]
Study design	Studies with or without a control group

### Study Selection and Quality Assessment

If at least one of the parameters, such as heart rate (HR), stroke volume (SV), CO, or ejection fraction (EF), under resting and stress conditions was assessed in a human patient population, studies were included. Any studies published before 1985 and publications that were not available in the English or German language, or which could not be accessed as full texts within the institutional subscriptions or the National Library license, were not considered. Studies that assessed stress conditions other than dynamic, isometric exercise, or dobutamine infusion as pharmacological stress were excluded. If less than 10 subjects were part of a study arm, these results were not included. Furthermore, we excluded review letters, comments, conference posters, and single case reports. According to these criteria, articles were screened on the title and abstract level before full texts were retrieved. Every cohort testing for several forms of stress on different intensity levels formed a separate study arm. Each article was reviewed by one reviewer (AW) before verification by a second reviewer (AB) was performed. In case of a disagreement, a third reviewer was involved in the review process (MK). Stepwise study assessment was guided by a modified version of the Downs and Black checklist (14). Studies were assessed for their reporting, external validity, internal validity, distribution, and adjustment for confounding variables, where appropriate as described previously (13) in more detail. Studies were categorized into low, moderate, and high quality based on their quality assessment scores.

### Data Extraction

If available, means and standard deviations under resting and different forms of stress conditions were extracted and documented. If unable to provide information on the variance, such studies were excluded from the analysis. If studies provided indexed SV or the cardiac index and body surface area (BSA), then the absolute SV and CO would be calculated. Data extraction included information on the clinical characteristics of study cohorts, such as sex, age, BSA, body mass index (BMI), and the New York Heart Association (NYHA) functional classification. Comorbidities such as arterial hypertension, atrial fibrillation, diabetes mellitus, and coronary artery disease (CAD), as well as medication usage (beta blockers, angiotensin-converting enzyme inhibitors (ACEi), angiotensin II receptor blockers (ARBs), and aldosterone antagonists), were also extracted. If available, information on cardiac resynchronization therapy (CRT) completed the baseline data. For further analyses and comparison of HFrEF or HFpEF patients with healthy subjects, we used results from a previously published analysis (13).

### Intensity Classification

Studies in which dynamic stress testing was performed were categorized as light, moderate, or high intensity according to the intensity stated in watts (W) during ergometric exercise (assuming a body weight of 60–80 kg) (15), the percentage of age-specific maximal HR [HR_*max*_ = 220 − age (years)] (16), or the statement of the authors regarding the intensity level. In the case of incongruity between these three indicators, we complied with the statement of the study’s authors for a final classification. Submaximal exercise capacity in patients with HF was commonly defined between 20 and 30 W (17–23), with load increments between 10 and 20 W and exhaustion above 30 W and was thus lower than in healthy subjects (17, 18, 24). This classification was applied for dynamic stress testing studies (Table 2).

**TABLE 2:** Intensity levels of stress testing.

Intensity	Dynamic exercise	Dobutamine stress
Light	Ergometer: < 20 W* HR max: ≤ 54% Statement: Light	0–10 μg/kg/min
Moderate	Ergometer: 20–30 W* HR max: 55–84% Statement: Submaximal/moderate	11–20 μg/kg/min
High	Ergometer: > 30 W* HR max ≥ 85% Statement: Exhaustion/symptom-limited	> 20 μg/kg/min

*Submaximal exercise capacity in patients with HF was commonly defined between 20 and 30 W (17–23), with load increments between 10 and 20 W and exhaustion above 30 W and was thus lower than in healthy subjects (17, 18, 24). This classification was applied for dynamic stress testing studies in this table.

We included studies that performed pharmacological stress testing using dobutamine. According to the well-established classifications, the intensity of pharmacological stress was categorized as light for a low-dose infusion of dobutamine of 0–10 μg/kg/min, as moderate for 11–20 μg/kg/min, and as high for a dose exceeding 20 μg/kg/min (25–28). Isometric stress tests were categorized as light intensity exercise tests, given that static contraction causes only a slight increase in HR or CO, mainly affecting mean arterial pressure and not being expected to reach the changes of higher levels of dynamic exercise (29). A summary of these criteria is illustrated in Table 2.

### Heart Failure Classification

According to 2016 and in line with the 2021 ESC guidelines, patients were classified as individuals with HFpEF when left ventricular ejection fraction (LVEF) was ≥ 50% (30). The 2012 ESC guidelines defined HFrEF when LVEF is below 35%, whereas the more recent 2016 guidelines changed this definition to an LVEF below 40%, and the 2021 ESC guidelines further changed the definition to below or equal 40%. An LVEF of 35–50% was considered a “gray area” in the 2012 guidelines, whereas more recent guidelines define a new class of HF individuals with mid-range/mildly reduced ejection fraction (HFmrEF) when LVEF is 40–49% (31, 32). There was, therefore, an inhomogeneity of classification among the studies investigating stress testing in HFrEF before and after 2016. The definition and terminology of HF according to LVEF are displayed in Table 3. For reasons regarding the simplification of our analysis, patients with an LVEF < 50% were classified as HFrEF when no separation to HFmrEF was made.

**TABLE 3:** Definition and terminology of heart failure (HF) related to left ventricular ejection fraction.

EF in%	< 40*	40–49	≥ 50
Classification according to ESC guidelines 2012 (31)	HFrEF (< 35%)	Gray area (35–50%)	HFpEF
Classification according to ESC guidelines 2016/2021* (32)	HFrEF	HFmrEF	HFpEF
Classification for analysis	HFrEF		HFpEF

*The 2021 ESC guidelines have changed the definition of HFrEF ≤ 40% and HFmrEF between 41 and 49. Classification for data analysis across studies from different time periods is shown in the bottom row.

### Statistical Analysis

The analyses were executed in STATA, version 15.1 (StataCorp, College Station, Texas, United States), by using the “*metan*” package. A multivariate meta-regression model was used to determine variables that potentially influenced outcome parameters. Correlations were investigated through univariate meta-regression. Furthermore, a pairwise meta-analysis was conducted in studies directly comparing different types of stress. Otherwise, study arms were grouped according to stress type and stress level, with the aim to obtain pooled estimates of changes. Furthermore, results were analyzed separately for HFrEF and HFpEF patients. Mean differences of hemodynamic parameters between rest and stress conditions, with respective standard errors of the difference between means, were calculated (33). Outcomes were pooled using a DerSimonian-Laird random effects model (34).

Heterogeneity was assessed using the Q-statistic and with a visual inspection of forest plots for all interventions and outcomes. Between-study variation, due to true heterogeneity, was measured using the *I*^2^ statistic (35), with values of 25% or higher indicating significant heterogeneity that supports the use of a random effects model (36, 37). Results are shown as absolute mean changes and with 95% confidence intervals (CIs) between resting and stress conditions, as well as a visualization in forest plots (Supplementary Material). A lack of overlap between the CIs of pooled changes indicated significant differences between the different stress types (37).

## Results

### Study Characteristics

**FIGURE 1:**
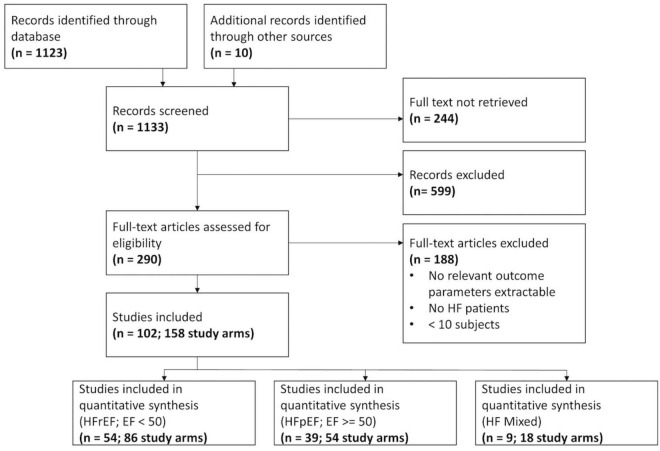
Flow chart of the Preferred Reporting Items for Systematic Reviews and Meta-Analyses (PRISMA).

A total of 1,123 references were extracted from the database. Ten additional studies were obtained from further sources, mainly as they were referenced in other studies. After screening at the title and abstract level, 290 full-texts were extracted. Notably, 102 studies examining stress testing in HFpEF and HFrEF patients with a total of 158 study arms, 9,298 subjects, and 9,764 stress examinations could be retrieved after screening the full-texts. Of note, 7,248 stress examinations were considered for further analysis after eliminating studies in which HFpEF and HFrEF could not be clearly assigned (*N* = 9). The Preferred Reporting Items for Systematic Reviews and Meta-Analyses (PRISMA) flow chart (Figure 1), the network of evidence (Supplementary Figure 1), and the list of included studies (Supplementary Table 2) show details of the study selection process. Mean absolute changes for HR, SV, CO, and EF from single-arm studies are shown in Supplementary Table 3. The results of the quality assessment are shown in Supplementary Table 4.

Additional single study arms were included, resulting in a total of 114 study arms (5,920 stress examinations) for dynamic exercise testing, 25 study arms (1,308 stress examinations) for pharmacological stress testing, and 1 study arm (20 stress examinations) for isometric exercise testing in a pooled analysis. Only one study (3 study arms) was found to directly compare dynamic, pharmacological, and isometric stress testing in HF patients (38). Three studies (6 study arms) could be obtained that directly matched HFpEF and HFrEF individuals (39–41).

Baseline characteristics for all patients with HFpEF or HFrEF undergoing dynamic or dobutamine stress testing are listed in Table 4. In *N* = 5 study arms (3%), atrial fibrillation was defined as an exclusion criterion, and in *N* = 15 (9.4%), atrial fibrillation was reported with an average of 15%. Due to the low number of study arms reporting on a minority of subjects with atrial fibrillation, the parameter was excluded from further analysis. Due to a low number of study arms of individuals undergoing isometric stress tests (*N* = 1), these were not considered for further analysis. Baseline tables for those HF patients comparing dynamic and pharmacological stress testing are shown in Supplementary Tables 5–7. HFrEF patients were generally younger than the HFpEF patients, with a lower BMI and a higher NYHA class, and were predominantly male, while HFpEF studies included more female patients. Within the dynamic exercise group of HFrEF, individuals tended to be both younger and with a better EF than the patients receiving dobutamine.

**TABLE 4:** Baseline characteristics for individuals with HF.

	HFrEF		HFpEF		Sign.
		Study arms reporting variable (N of tests)		Study arms reporting variable (N of tests)	*P*-value
Total N of stress tests		86 (5,027)		54 (2221)	
Age, years	62 (57–66)	86 (5,027)	67.2 (65–70)	54 (2,221)	0.0001
Male,%	80.28 (72.46–93.75)	86 (5,027)	37.46 (28.13–52.5)	54 (2,221)	0.0001
BSA, m^2^	1.89 (1.86–1.94)	8 (399)	1.9 (1.71–1.99)	17 (652)	0.8613
BMI, kg/m^2^	27 (26.4–28.5)	23 (1,164)	31 (29.8–33.6)	43 (1,774)	0.0001
NYHA class	2.48 (2.20–2.92)	72 (4,674)	2.27 (2.00–2.46)	30 (1,018)	0.0009
CRT,%	25.1 (12–100)	29 (1,954)			
ACE,%	82.61 (74–92)	51 (3,718)	44 (36–53)	17 (779)	0.0001
ARB,%	20 (13–26)	27 (2,338)	28 (19–33)	15 (686)	0.0275
Beta blockers,%	88 (78–93)	65 (3,866)	64 (44–71)	49 (2,086)	0.0001
Aldosterone antagonist,%	53.25 (48.1–69)	42 (3,194)	24 (9–26)	5 (171)	0.0007
Hypertension,%	42 (28–62)	30 (1,726)	80 (69–94)	48 (1,938)	0.0001
Diabetes mellitus,%	23 (17–33)	35 (1,769)	24.5 (15–36)	46 (2,059)	0.6644
CAD,%	56 (38–70)	49 (2,207)	19 (10–36)	22 (796)	0.0001
Resting HR, bpm	72 (69–78)	85 (4,925)	69 (67–75)	54 (2,221)	0.0219
Resting SV, ml	64.85 (55–82)	6 (201)	71 (65–74.1)	13 (409)	0.5686
Resting CO, L/min	3.9 (3.5–4.3)	19 (723)	5.1 (4.9–5.1)	47 (2,010)	0.0001
Resting EF,%	30.15 (26.5–35)	84 (4,958)	62 (60–63)	17 (596)	0.0001
Light intensity,%	3	3 (64)	4	2 (31)	
Moderate intensity,%	14	12 (597)	22	12 (455)	
High intensity,%	83	71 (4,366)	74	40 (1,735)	

*Values are reported as medians (interquartile range). ACE, Angiotensin-converting enzyme inhibitors; ARB, Angiotensin II Receptor Blockers; BMI, body mass index; BSA, body surface area; CAD, Coronary artery disease; CRT, cardiac resynchronization therapy; HR, heart rate; SV, stroke volume; CO, cardiac output; EF, ejection fraction.*

### Pooled Effects of Rest-Stress Changes From Single-Arm Studies

We reported effect measures and, where multiple studies were available, pooled effects in (1) light intensity, (2) moderate intensity, and (3) high intensity:

1. Dynamic exercise with light intensity was reported in one study: Compared to resting baseline values, HR increased by 21 bpm (95% CI 0.84–41.16), SV by 40 ml (95% CI 22.03–57.97), and CO by 5.5 L/min (95% CI 3.45–7.55). Low-dose dobutamine infusion (5–10 μg/kg/min) resulted in the changes of HR by 8.9 bpm (95% CI 5.13–12.67; *I*^2^ = 0.0%), SV 9 ml (95% CI –3.23 to 21.23; reported in one study), CO 0.97 L/min (95% CI 0.62–1.32; *I*^2^ = 0.0%), and EF 4.65% (95% CI 2.2–7.11; *I*^2^ = 0.0%). Pooled changes of isometric exercise were reported in one study: HR 7 bpm (95% CI –0.11 to 14.11), CO 0 L/min (95% CI –0.89 to 0.89), and –5% for EF (95% CI –8.51 to –1.49).
2. Within the moderate dynamic intensity group, pooled estimates of changes in HR were 21.23 bpm (95% CI 19.69–22.76; *I*^2^ = 0.0), SV 6.02 ml (95% CI –0.9 to 12.94; *I*^2^ = 67.0%), CO 1.83 L/min (95% CI 1.32–2.33; *I*^2^ = 66.6%), and EF 4.59% (95% CI 1.08–8.11; *I*^2^ = 0.0%). Moderate dosage of dobutamine infusion (11–20 μg/kg/min) resulted in HR changes of 18.3 bpm (95% CI 10.42–26.17; reported in one study), SV –0.61 ml (95% CI –29.02 to 27.81; *I*^2^ = 88.8%), CO 1.65 L/min (95% CI 0.61–2.69; *I*^2^ = 71.3%), and EF 6.06% (95% CI 3.23–8.89; *I*^2^ = 82.5%).
3. High dynamic exercise increased HR by 45.69 bpm (95% CI 44.51–46.88; *I*^2^ = 98.4%), SV by 13.49 ml (95% CI 6.87–20.10; *I*^2^ = 68.5%), CO by 3.41 L/min (95% CI 2.86–3.95; *I*^2^ = 86.3%), and EF by 3.69% (95% CI 2.49–4.89; *I*^2^ = 52.9%). For high dosage of dobutamine infusion (11–20 μg/kg/min), changes in HR were 40.72 bpm (95% CI 33.93–47.50; *I*^2^ = 92.7%), and changes in EF were 11.87% (95% CI 10.06–13.67; *I*^2^ = 44.7%). There were not enough studies available investigating changes in SV and CO for high-intensity pharmacological stress testing. A detailed summary of all findings and a subgroup analysis for both HF groups is available in the Supplementary Figures 2–4.

### Comparison Between HFrEF, HFpEF, and Healthy Subjects

We identified six categories in which changes in HR, SV, CO, or EF from single study arms could be compared between patients with HFpEF and HFrEF, where at least two studies were available for both disease groups at the same intensity level and stress type (marked in bold in Table 5). Those included (1) HR change by dynamic exercise at moderate intensity, (2) HR change by dynamic exercise at high intensity, (3) HR change by pharmacological exercise at high intensity, (4) SV increases by dynamic exercise at high intensity, (5) CO increases by dynamic exercise at high intensity, and (6) EF increases by dynamic exercise at high intensity.

**TABLE 5:** Available stress testing studies for HF individuals (number of stress tests).

		Light intensity		Moderate intensity		High intensity	
Parameter	Stress type	HFrEF	HFpEF	HFrEF	HFpEF	HFrEF	HFpEF
Heart rate change (HR)	Dynamic	0 studies	1 study (*N* = 11)	**2 studies** (*N* = 52)	**11 studies** **(*N*** = **435)**	**38 studies (*N*** = **3,671)**	**32 studies (*N*** = **1,647)**
	Pharmacologic	2 studies (*N* = 64)	0 studies	7 studies (*N* = 545)	1 study (*N* = 20)	**8 studies (*N*** = **591)**	**2 studies (*N*** = **88)**
Stroke volume (SV)	Dynamic	0 studies	1 study (*N* = 11)	0 studies	7 studies (*N* = 250)	**3 studies (*N*** = **131)**	**5 studies (*N*** = **148)**
	Pharmacologic	1 study (*N* = 22)	0 studies	1 study (*N* = 46)	0 studies	0 studies	0 studies
Cardiac output (CO)	Dynamic	0 studies	1 study (*N* = 11)	0 studies	8 studies (*N* = 347)	**9 studies (*N*** = **597)**	**6 studies (*N*** = **198)**
	Pharmacologic	2 studies (*N* = 64)	0 studies	2 studies (*N* = 60)	1 study (*N* = 20)	0 studies	0 studies
Ejection fraction (EF)	Dynamic	0 studies	0 studies	0 studies	2 studies (*N* = 51)	**12 studies (*N*** = **928)**	**2 studies (*N*** = **37)**
	Pharmacologic	2 studies (*N* = 64)	0 studies	6 studies (*N* = 510)	1 study (*N* = 20)	5 studies (*N* = 346)	1 study (*N* = 47)

*Studies can include multiple study arms. Six categories were identified for direct comparison where at least two studies were available for both disease groups (marked in bold).*

**FIGURE 2:**
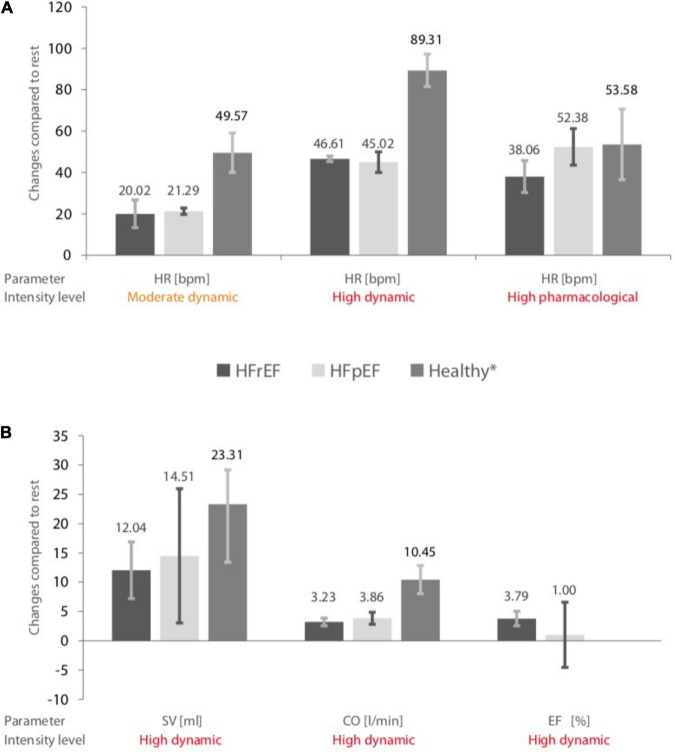
**(A)** Pooled changes in heart rate (HR) during different stress levels in heart failure with preserved ejection fraction (HFrEF) patients, heart failure with reduced ejection fraction (HFpEF) patients, and healthy controls. **(B)** Changes in stroke volume (SV), cardiac output (CO), and ejection fraction (EF) during a high dynamic stress test in HFrEF, HFpEF patients, and healthy controls. The error bars indicate mean values ± standard deviations. *Data obtained from a previous meta-analysis (13). Included heart failure study arms and sample sizes are given in Table 5.

For eligible studies, where at least two studies were available in each HF group, mean absolute changes and 95% CIs of HR, SV, CO, and EF of HF subjects, as well as in healthy controls (13), are visually summarized in Figure 2.

1. The changes in HR by moderate dynamic exercise in HFrEF patients were 20.02 bpm (95% CI 13.31–26.74; *I*^2^ = 0.0), 21.29 bpm (95% CI 19.72–22.87; *I*^2^ = 0.0) in HFpEF patients, and 49.57 bpm (95% CI 40.03–59.1; *I*^2^ = 97.0%) in healthy controls.
2. By high dynamic exercise in HFrEF individuals, pooled estimates of changes in HR were 46.61 bpm (95% CI 45.22–48.01; *I*^2^ = 98.8%), 45.02 bpm (95% CI 40.03–50.01; *I*^2^ = 95.8%) for HFpEF patients, and 89.31 bpm (95% CI 81.46–97.17; *I*^2^ = 97.6%) for healthy subjects.
3. High pharmacological stress in HFrEF patients resulted in changes in HR of 38.06 bpm (95% CI 30.36–45.76; *I*^2^ = 93.1%), in HFpEF patients of 52.38 bpm (95% CI 43.56–61.20; *I*^2^ = 74.8%), and in healthy subjects of 53.58 bpm (95% CI 36.53–70.64; *I*^2^ = 98.4%).
4. High dynamic exercise in HFrEF subjects resulted in a change in SV of 12.04 ml (95% CI 7.19–16.90; *I*^2^ = 0.0%), in HFpEF patients of 14.51 ml (95% CI 3.04–25.97; *I*^2^ = 80.6%), and in healthy subjects of 21.31 ml (95% CI 13.42–29.21; *I*^2^ = 91.1%).
5. High dynamic exercise in HFrEF patients resulted in changes in CO of 3.23 L/min (95% CI 2.56–3.89; *I*^2^ = 87.9%), in HFpEF patients of 3.86 L/min (95% CI 2.82–4.89; *I*^2^ = 84.0%), and in healthy subjects of 10.45 L/min (95% CI 8.04–12.85; *I*^2^ = 98.9%).
6. High dynamic exercise in HFrEF patients increased EF by 3.79% (95% CI 2.56–5.03; *I*^2^ = 55.6%) and by 1% (95% CI –4.59 to 6.59; *I*^2^ = 0.0%) in HFpEF patients. There were no data available for changes in EF in healthy subjects.

High-intensity dynamic stress testing represented 73% of the data included for comparison. A detailed assessment of study heterogeneity and a comparison is found in Supplementary Material, including a visual representation as forest plots (Supplementary Figures 5–31).

### Effects of Stress Type, Intensity Level, and Age on Stress-Induced Hemodynamic Changes

The results of a multivariable meta-regression model [*p* < 0.001, *F*(6, 131) = 27.5, adjusted *R*^2^ = 57.91%] indicate that high-intensity level stress testing was associated with a greater absolute increase in HR, as compared to light intensity level stress testing (45.69 bpm vs. 21.0 bpm; Coef., 31.2; 95% CI 19.5–42.9; *p* < 0.001). Furthermore, age was associated with HR changes (Coef., –0.55; 95% CI –0.88 to –0.21; *p* = 0.002) within the combined model. No differences were found for moderate intensity compared to light intensity stress testing (*p* = 0.112) nor for pharmacological compared to dynamic stress testing (*p* = 0.130). Detailed results of the model are shown in Supplementary Table 8.

Pharmacological stress testing (Coef., 6.7; 95% CI 4.5–8.9; 95% CI 4.5–9; *p* < 0.001) and high-intensity level stress testing (Coef., 5.9; 95% CI 1.9–9.9; *p* = 0.005) were both associated with higher increases in EF under stress conditions [model *p* < 0.001, *F*(6, 39) 10.26, adjusted *R*^2^ = 71.22%]. No associations of intensity level or stress type were found for SV or CO. HF group allocation (HFpEF or HFrEF) was not associated with stress-induced changes in HR, SV, CO, or EF. All subsequent analyses of HR, SV, and CO were performed across studies from both HF groups and all intervention types and included separation for different intensity levels.

**FIGURE 3:**
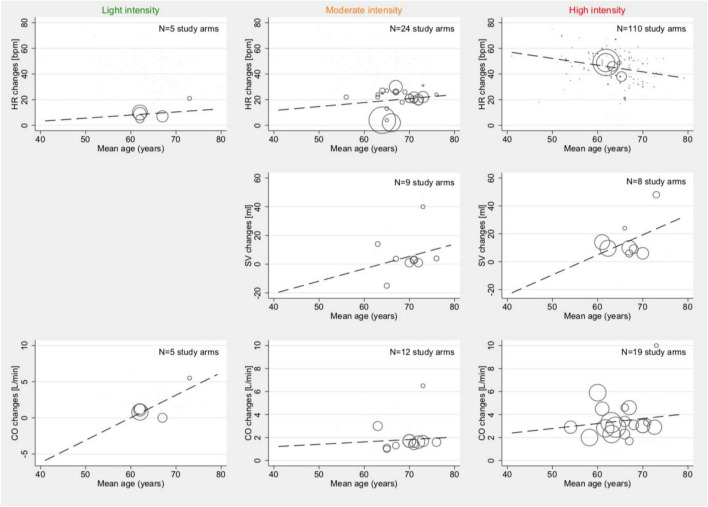
Associations between mean age and mean absolute changes in HR (top row), SV (2nd row), and CO (bottom row) among studies reporting outcomes with light- (1st column), moderate- (2nd column), and high intensity (3rd column). Studies with both HFpEF and HFrEF patients under dynamic and pharmacological stress testing were included. Bubble size indicates the sample size of one study arm in relation to other study arms within the same category.

Univariable meta-regression, stratified by intensity levels, showed an association with age and HR changes within the high-intensity study arm (Coef., –0.52; 95% CI –0.86 to –0.18; *p* = 0.003; Cons., 78.3; 95% CI 56.8–99.8). No other significant correlations between the age of patients within a study arm and changes in HR, changes in SV, or changes in CO were found (*p* > 0.05). HR changes for light intensity were *p* = 0.734, and those for moderate intensity were *p* = 0.461. SV changes for moderate intensity (*p* = 0.680) and high intensity (*p* = 0.284) were calculated, without data availability for light intensity. CO changes for light intensity were *p* = 0.168, those for moderate intensity were *p* = 0.826, and those for high intensity were *p* = 0.565. Graphical plots of the meta-regression models are shown in Figure 3.

### Effects of Resting Conditions on Stress-Induced Hemodynamic Changes

Meta-regression did not show associations between the average resting HR and those reported HR changes during light (*p* = 0.675) and medium intensity (*p* = 0.219) stress testing. For high intensity, an inconclusive association was demonstrated (Coef., 0.31; 95% CI –0.004 to 0.630; *p* = 0.053; Cons., 22.6; 95% CI –0.6 to 45.8). Whereas no sufficient amount of studies was available to assess SV changes in light activity, there was a relevant inverse correlation between the reported average resting SV and SV changes during moderate-intensity stress testing (Coef., –1.3; 95% CI –2.6 to –0.04; *p* = 0.044; Cons., 98.3; 95% CI 7.0–189.7). No such correlation was found for SV changes during high-intensity stress testing.

**FIGURE 4:**
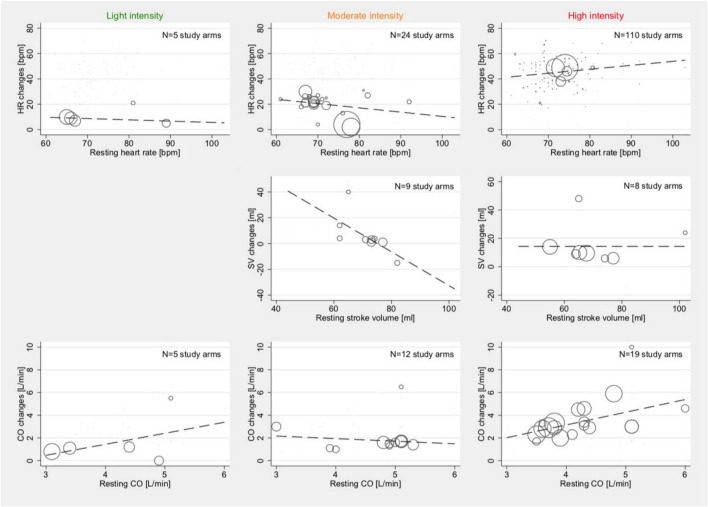
Associations between resting conditions and changes under stress: HR (top row), SV (2nd row), and CO (bottom row) among studies reporting outcome with light- (1st column), moderate- (2nd column), and high intensity (3rd column). Studies with both HFpEF and HFrEF patients under dynamic and pharmacological stress testing were included. Bubble size indicates the sample size of one study arm in relation to other study arms within the same category.

Resting CO was not associated with CO changes under light-intensity (*p* = 0.476) and moderate-intensity (*p* = 0.625) stress testing but was correlated during high activity (Coef., 1.12; 95% CI 0.14–2.1; *p* = 0.027; Cons., –1.3; 95% CI –0.5 to 2.8). The results of these meta-regression analyses are illustrated in Figure 4.

### Effects of Reported Treatment on Stress-Induced Hemodynamic Changes and Resting Conditions

Associations between stress testing-induced changes in hemodynamic parameters and reported treatment were analyzed for HR changes due to an insufficient amount of studies having reported data on treatment for SV and CO. No associations between reported treatment and HR changes were found for light- and moderate-intensity stress levels for either HFrEF or HFpEF patients.

**FIGURE 5:**
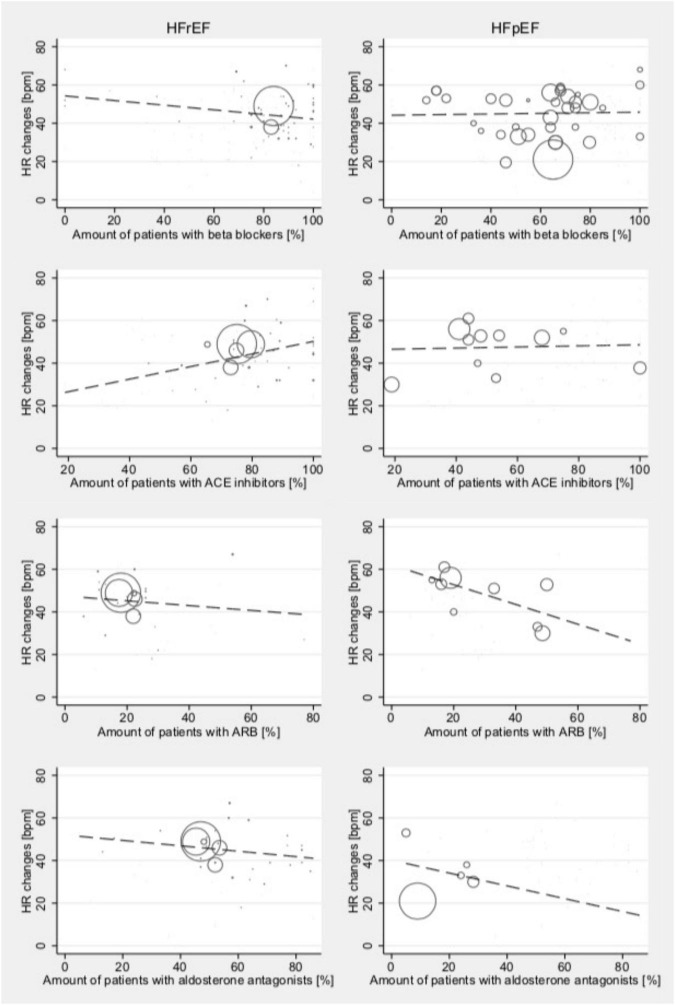
Relationship between medical treatment and mean absolute stress testing-induced HR changes in those studies reporting outcomes for HFrEF (left) and HFpEF (right) patients at high-intensity levels. Studies include both dynamic and pharmacological stress testing. Bubble size indicates study sample size.

Meta-regression models indicated an association between the proportion of patients taking ACEi and the stress testing-induced changes in HR among studies reporting data for HFrEF patients tested at high-intensity levels (Coef., 0.30; 95% CI 0.03–0.56; *p* = 0.028; Cons., 20.71; 95% CI –1.1 to 42.5; Figure 5). No such effects were seen in HFpEF patients. In patients with HFpEF, there was an inconclusive association between the intake of ARB and HR changes (Coef., –0.46; 95% CI –0.96 to 0.03; *p* = 0.061; Cons., 62.0; 95% CI –45.5 to 78.5); this was not seen in HFrEF. In patients with HFrEF, there was an inconclusive association between the intake of beta blockers and HR changes (Coef., –0.12; 95% CI –0.26 to 0.01; *p* = 0.079; Cons., 54.3; 95% CI –42.8 to 65.7); this was not seen in patients with HFpEF. Furthermore, there were no associations between treatment with aldosterone antagonists and stress testing-induced change in HR at any intensity level for HFrEF or HFpEF patients. No effects were found for CRT.

**FIGURE 6:**
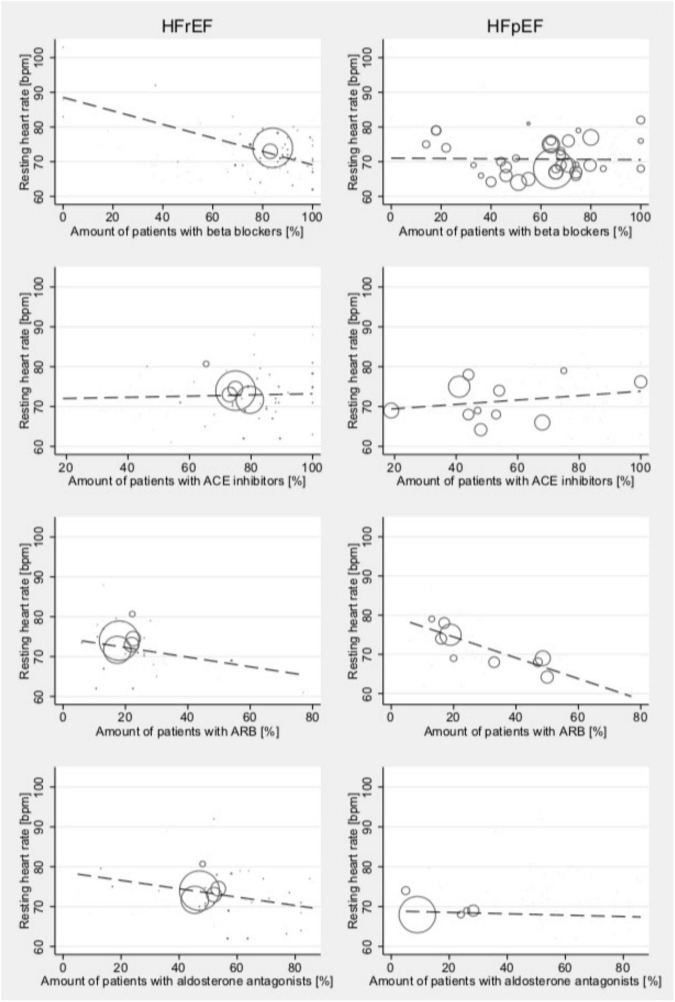
Relationship between medical treatment and the resting HR among studies reporting outcomes for HFrEF (left) and HFpEF (right) patients test at high-intensity levels. Studies include both dynamic and pharmacological stress testing. Bubble size indicates study sample size.

As treatment can impact resting HR rather than affecting changes under exercise conditions, the associations between different treatment methods and the resting HR were analyzed (Figure 6): resting HR was associated with reported beta-blocker intake (Coef., 0.01; 95% CI –0.12 to 0.15; *p* < 0.001; Cons., 88.5; 95% CI 82.5–94.6) in HFrEF, while this effect was not found in patients with HFpEF. Resting HR in HFpEF was associated with ARB intake (Coef., –0.27; 95% CI –0.47 to –0.07; *p* = 0.016; Cons., 79.8; 95% CI 73.2–86.4). No other associations of medical treatment and resting HR were found.

### Comparative Studies

We identified one study that directly compared pharmacological, dynamic (bicycle exercise), and isometric stress testing in 20 patients (38). In this study, HR change was at 7 bpm with isometric exercise (95% CI –0.11 to 14.11), 26 bpm (95% CI 19.26–32.74) with dobutamine infusion (20 μg/kg/min), and 38 bpm (95% CI 27.12–48.88) when stressed dynamically with high intensity.

We also identified 3 studies (6 study arms) directly comparing HFpEF with HFrEF patients during high-intensity dynamic stress testing (39–41). Changes in CO and EF were analyzed in one study with no difference between HFrEF and HFpEF patients. HR changes were tested in all three studies (Table 6).

**TABLE 6:** Studies directly comparing HFpEF with HFrEF patients during high-intensity dynamic stress testing.

Study	*N*	Parameter	HFrEF	HFpEF
Farr et al. (39)^a^	HFrEF: *N* = 185 HFpEF: *N* = 43	HR change, bpm	44 (40–48)	37 (30–45)
Sugimoto et al. (40)^b^	HFrEF: *N* = 49 HFpEF: *N* = 20	HR change, bpm	37 (31–43)	38 (27–49)
		CO change, L/min	2.3 (1.64–2.96)	2.9 (2.10–3.70)
		EF change	3% (–0.85–6.85)	1% (–7.73–9.73)
Wang et al. (41)^c^	HFrEF: *N* = 50 HFpEF: *N* = 80	HR change, bpm	62 (55–69)	54 (49–59)

*^a^HFrEF EF < 50%, HFpEF EF ≥ 50%.*

*^b^HFrEF EF < 40%, HFpEF EF > 50%.*

*^c^HFrEF EF < 50%, HFpEF EF ≥ 50%.*

## Discussion

This systematic review and meta-analysis reports on the stress-induced changes of hemodynamic parameters in patients with HFrEF and HFpEF. Despite the limited availability of comparative studies, pooled changes of the included study arms are presented. In the activity levels where sufficient data from both HF groups were available, the results were compared between both HF groups as well as with data previously reported in healthy populations (13). Most of the data included in this comparison were from high intensity examinations.

In studies where HF patients were tested with dynamic exercise at moderate or high intensity, smaller changes in HR and CO were found when compared to healthy controls. When stressed pharmacologically at high intensity, changes in HR were lower and 95% CIs marginally overlapped with those from healthy controls. Although CO at high dynamic stress testing was lower in HF than in controls, there were no differences found in SV between both HF groups and controls.

Whereas HR increases during exercise follow a typical pattern in healthy individuals, such regulation is commonly compromised in HF patients (42). Explanations for an attenuated HR increase, in response to dynamic exercise, include the use of HR-lowering drugs (typically beta blockers), as well as lower exercise intensity levels compared to healthy individuals. Such an impaired chronotropic reserve has been described in HF patients due to imbalances in the autonomic nervous system (43).

Thus, examining HR responses to incremental workload or to dobutamine infusion may help to identify patients with HF or to assess the severity of autonomic dysfunction. While both methods have their unique advantages and disadvantages, our results suggest that for the distinction of HF patients from healthy individuals, the evaluation of HR and CO changes in response to dynamic stress testing may be more appropriate than pharmacological stress testing. Furthermore, dynamic exercise testing is typically considered the most physiological type of stress (44). Nevertheless, dynamic stress testing includes the assessment of a personal maximum or submaximal workload, which can be substantially altered in HF. In conjunction with wearable devices, models based on such changes were recently shown to be capable of predicting the outcome of standardized 6-min walk tests in patients with heart disease (45). The adaptation of such models, as well as surveillance strategies to disease-specific aspects of HF, may help to better identify patients at risk providing data-driven approaches to patients and caregivers that can help to detect deterioration early on (46).

One study arm comprising 20 stress examinations was considered for isometric exercise testing. Additionally, and after the date of the final search, Blum et al. published a study comparing strain during handgrip exercise between HF groups in 53 patients. This recent study includes information on HR responses and addresses particular responses of HFmrEF patients to isometric exercise (47). Furthermore, no sufficient data for comparison were available in HFmrEF patients. Studies with older classifications of HFrEF may, however, include HFmrEF patients without allowing for further distinction.

The HF is often accompanied by cardiac and non-cardiac disease, as well as potential confounders, which may contribute additionally to reduced exercise capacity and which may limit the patient’s ability to perform dynamic exercise testing adequately. Etiologies and treatment regimens for HFrEF and HFpEF vastly differ, and in line with these concepts, relevant group differences were found: HFpEF patients were older, predominantly female, had a higher BMI, were in a lower NYHA class, and were less frequently characterized with CAD. Only a few studies reported on a small minority of subjects with atrial fibrillation. This was in clear contrast to the existing literature where around 50% of all HF patients have been described to also suffer from atrial fibrillation and 30% of all patients with atrial fibrillation to suffer from HF (48). Medication profiles differed according to current treatment practice (main differences: ACEi were used by 83% of HFrEF patients and by 44% in HFpEF; beta blockers were used by 88% in HFrEF and by only 64% in HFpEF; aldosterone antagonists were used by 53% of HFrEF patients and only 24% in HFpEF). By including the HF group in our analysis, these differences, as well as other potentially unobserved variables, were indirectly considered.

Relevant group characteristics with a sufficient amount of studies were reported for stress type, stress intensity, age, patient group allocation, and medication. The magnitude of exercise-induced HR responses in patients with HF did not substantially differ between studies investigating dynamic stress testing and pharmacological exercise, respectively. Nevertheless, it remains open to further evaluate whether the assessment of chronotropic competency by exercise testing and pharmacological stress testing can be considered as an alternative for those patients who do not tolerate dynamic testing. Furthermore, HR changes were inversely correlated with age within high-intensity exercise study arms. No consistent reporting was found for pacemaker use, although the devices may influence HR response under stress conditions. Nevertheless, CRTs were reported in HFrEF, as no consensus for a benefit in HFpEF exists.

This meta-analysis was not designed to assess the effects of daily pharmacotherapy on stress testing in HF, as this would require comparable studies in combination with standardized stress testing protocols. Some authors, however, provide population-based medication data. The increase in HR was more pronounced, and thus, more physiological patients were treated with ACEi in HFrEF studies. The positive effects associated with the inhibition of the renin-angiotensin-aldosterone system (RAAS) were previously shown to lead to a significant reduction in mortality and morbidity rates, and in turn, ACEi are considered first-line treatment for patients with HFrEF (49). In HFpEF, no such association of ACEi intake and HR changes under stress was found. However, as treatment with ACEi is not commonly recommended, the average number of patients taking ACEi was lower within the HFpEF study arms. The effects of RAAS inhibition in HFpEF are less well understood, and a benefit in reducing the mortality rate has not been demonstrated (50). Moreover, an inconclusive inverse association between ARB intake and HR changes under stress was observed in HFpEF but not in HFrEF. In line with these results, the findings of our analyses suggest that RAAS inhibition might be of lesser benefit in HFpEF than in the HFrEF populations. In patients who do not tolerate ACEi, ARBs are recommended and frequently used alternatives. However, it is evident that ACEi do not have the same inhibitory effect on RAAS activity and, therefore, that their beneficial effects differ from ARB (51), which could explain this discrepancy in the findings of our current study.

We did not find sufficient data on the more recently advocated combined use of ARBs and neprilysin inhibitors (i.e., valsartan + sacubitril) within the analyzed study arms, which has been proposed particularly in HF patients with symptoms under stress conditions (52). Whereas the majority of patients within HFrEF study arms were under beta-blocker therapy, the use of beta blockers in HFpEF is still under controversial discussion. In HFrEF, the resting HR was inversely correlated with the number of patients for beta-blocker intake. No such effect was seen in HFpEF. Neither relevant effects on the number of patients taking beta blockers nor aldosterone antagonists on HR changes under exercise were found. Although heterogeneously reported in the analyzed studies, the inverse correlation between resting HR and beta-blocker use can be seen as an indicator for medication intake before stress testing. Elimination of the beta-blocker effects would require withholding the drug for 5 half-lives (53). As this is known to be rarely done, current recommendations by the American Society of Echocardiography suggest that the discontinuation of beta blockers is not essential but may require intermediate (15–20 μg/kg/min) dobutamine doses (53).

### Limitations

The majority of the studies were observational trials for HFpEF or HFrEF patients, and mainly, our results are based on a comparison of single study arms. Only three studies were identified which had directly compared the two HF groups. The results of these studies were in line with our findings from the single-arm analysis, confirming that exercise-induced HR changes are similar between both HF groups. Nevertheless, comparative studies of stratified HF populations, as well as HFmrEF patient populations in a standardized exercise protocol, would be highly desirable for an improved disease understanding.

For some intensity/parameter categories, only a few data were available (Table 5), and the uncertainty of pooled changes was consequently high. Therefore, the stress responses of HFpEF and HFrEF patients were only compared when at least two studies were available for each disease group at the same intensity level.

The intensities for dynamic stress testing used in the analyzed studies were low compared to intensities for a variety of different sports activities in healthy individuals (15). When compared to data from healthy subjects, lower HR changes during exercise found in HF patients may be explained by this effect. The classification of exercise intensities, however, was adapted according to the predefined lower submaximal exercise load targets and load increments in HF (17–22, 24). Nevertheless, large heterogeneity exists between the classification of exercise and to classifications in healthy cohorts. Subjective submaximal exercise and exhaustion, as well as symptoms, were commonly instantiated criteria as stated by authors in a majority of the studies. Due to a lack of studies that subjected HF patients to more intensive stress conditions, we were not able to systematically analyze hemodynamic changes for higher workloads.

Relevant heterogeneity can also be found in the dynamic exercise type and stress testing protocols. Responses to different protocols, load increments as well as responses to treadmill, supine, and upright bicycle exercise testing are known to differ. However, information on test protocols is not consistently available, and subgrouping according to available information did not reveal relevant differences. The study, therefore, further emphasizes the need for standardized stress testing protocols, transparent reporting, and for data-sharing initiatives to allow for more detailed network meta-analyses.

## Conclusion and Outlook

Reference values presented in this review can help to estimate the expected range of hemodynamic and circulatory responses in patients with HF. This may contribute to a better disease understanding, future study planning, and patient-specific predictive models. Although based on different etiologies and having differing baseline characteristics, no substantial differences in chronotropic reactions, changes in SV, or CO were found between HFrEF and HFpEF. When compared to healthy individuals, exercise tolerance, as well as associated HR and CO increases under moderate-high dynamic stress, was substantially impaired within HF patients and may reflect a relevant aspect of disease burden.

## Data Availability Statement

The raw data supporting the conclusions of this article are publically available. Supplementary Table 2 includes a full overview of all included studies.

## Author Contributions

AB, MS-K, DF, LG, and MK conceptualized and designed the study. AW and AB conducted the study selection. AW and MK performed the quality assessment. AW extracted the data. LG and MK conducted the statistical analysis. AB and MK drafted the manuscript. KR, MS-K, LG, SK, DM, BP, FB, and TK revised the manuscript. All authors interpreted the data and approved the final version of the manuscript.

## Conflict of Interest

The authors declare that the research was conducted in the absence of any commercial or financial relationships that could be construed as a potential conflict of interest.

## Publisher’s Note

All claims expressed in this article are solely those of the authors and do not necessarily represent those of their affiliated organizations, or those of the publisher, the editors and the reviewers. Any product that may be evaluated in this article, or claim that may be made by its manufacturer, is not guaranteed or endorsed by the publisher.
