# Supplementary material for: Hemodynamic Changes During Physiological and Pharmacological Stress Testing in Patients With Heart Failure: A Systematic Review and Meta-Analysis
Source: Front Cardiovasc Med. 2022 Apr 19;9:718114. doi: 10.3389/fcvm.2022.718114 (PMC9062977; doi:10.3389/fcvm.2022.718114)
Supplement: Supplementary file 1 [file Data_Sheet_1.pdf]

Supplementary Material

Hemodynamic Changes During Physiological and Pharmacological Stress Testing in Patients with Heart Failure – a *Systematic Review and Meta-Analysis*

frontiers

Supplementary tables

Table S1. Search terms used for database search.

PubMed, 29 February 2020		N
1	a) MRI/"Magnetic resonance" / "Magnetic resonance imaging"[Mesh]	750.024
	b) Echocardiography / "Echocardiography"[Mesh]	170.570
	c) ECG / "Electrocardiography"[Mesh]	226.022
2	a) Exercise / "Exercise"[Mesh] / "Exercise Test"[Mesh]	399.427
	b) Dobutamine*/ "Dobutamine"[Mesh]	9.512
	c) Handgrip / Isometric exercise / "Isometric Contraction"[Mesh]	409.851
3	a) "Heart failure" / "Heart failure"[Mesh]	194.756
4	a) Flow / Blood flow / Aortic flow / Peak flow / "Blood Flow Velocity"[Mesh] / "Regional Blood Flow"[Mesh]	777.363
		49.059
	b) "Stroke volume" / "Stroke index"/ "Stroke volume"[Mesh]	107.622
	c) "Cardiac output" / "Cardiac index" / "Cardiac output"[Mesh]	19.009
		16.395
	d) HRV / "Heart rate variability"	23.629
5	**(1a OR 1b OR 1c) AND (3a) AND (4a OR 4b OR 4c OR 4d)**	**11.082**
6	**(5) AND (2a OR 2b OR 2c)**	1.502
7	Limit to: Humans; English, German; Publication after 1985/01/01	1.303
8	Limit to: Full text availability	**1.122**

Table S2 List of included studies

First Author	Publication	Location	Patient number	Mean age (yrs)	Intervention	Intensity	Modality	Relevant outcomes	Comments	Quality
Abinader (1999)	Effects of Low Altitude on Exercise Performance in Patients With Congestive Heart Failure After Healing of Acute Myocardial Infarction	Israel	12	57	treadmill	exhaustion	ecg	HR	Haifa	9/19 moderate
Acanfora (2016)	Exercise training effects on elderly and middle-age patients with chronic heart failure after acute decompensation: A randomized, controlled trial	Italy	16	53	bicycle ergometer	symptom-limited	echocardiography	HR, EF		14/19 high
Agostoni (2010)	Relationship of resting hemoglobin concentration to peak oxygen uptake in heart failure patients	Italy	523	61,9	bicycle ergometer	exhaustion	ecg	HR		10/17 moderate
Agostoni (2010) 2	Effects of β-blockers on ventilation efficiency in heart failure	Italy	187	63,3	bicycle ergometer	exhaustion	ecg	HR		11/17 high

Aslanger (2015)	Effects of exercise on postexercise ventricular–arterial coupling and pulsatile efficiency in patients with systolic dysfunction	France	30	54	bicycle ergometer	exhaustion	echocardiography	HR		13/19 moderate
Bandera (2017)	Mitral regurgitation in heart failure: insights from CPET combined with exercise echocardiography	Italy	25	67	supine ergometer	symptom-limited	echocardiography	HR, LVEF, CO		12/17 high
Bhella (2011)	Abnormal haemodynamic response to exercise in heart failure with preserved ejection fraction	USA	11	73	treadmill	exhaustion	echocardiography	HR, CO, CI, SV, SVI	Same Patients	10/19 moderate
Borlaug (2006)	Impaired Chronotropic and Vasodilator Reserves Limit Exercise Capacity in Patients With Heart Failure and a Preserved Ejection Fraction	USA	17	65	bicycle ergometer	exhaustion	echocardiography	HR		11/19 moderate
Borlaug (2011)	Exercise Hemodynamics Enhance Diagnosis of Early Heart Failure with Preserved Ejection Fraction	USA	32	65	supine ergometer	exhaustion	echocardiography	HR, CI		10/19 moderate
Borlaug (2016)	Abnormal right ventricular-pulmonary artery coupling with exercise in heart failure with preserved ejection fraction	USA	50	70	supine ergometer	submaximal	echocardiography	HR, CO, SV	Same Patients	13/19 moderate

Cahalin (2013)	Heart rate recovery after the 6 min walk test rather than distance ambulated is a powerful prognostic indicator in heart failure with reduced and preserved ejection fraction: a comparison with cardiopulmonary exercise testing	Italy	50	64,5	bicycle ergometer	symptom-limited	ecg	HR		12/17 high
Chattopadhyay (2008)	The effect of pharmacological stress on intraventricular dyssynchrony in left ventricular systolic dysfunction	UK	30	69	dobutamine	40 μg/kg/min	echocardiography	HR		14/19 high
Chattopadhyay (2009)	Lack of Diastolic Reserve in Patients With Heart Failure and Normal Ejection Fraction	UK	41	70	dobutamine	40 μg/kg/min	echocardiography	HR		15/19 high
Choi (2006)	Factors Associated With Decreased Cerebral Blood Flow in Congestive Heart Failure Secondary to Idiopathic Dilated Cardiomyopathy	Korea	52	41	bicycle ergometer	symptom-limited	echocardiography	HR		11/19 moderate
Ciampi (2009)	Identification of responders to cardiac resynchronization therapy by contractile reserveduringstressechocardiography	Italy	69	70	dobutamine	40 μg/kg/min	echocardiography	HR, EDV, ESV, LVEF		12/17 high

Ciampi (2010)	Pressure-Volume Relationship During Dobutamine Stress Echocardiography Predicts Exercise Tolerance in Patients with Congestive Heart Failure	Italy	84	68	dobutamine	40 µg/kg/min	echocardiography	HR, LVEDV, LVESV, EF		12/17 high
Ciampi (2010) 2	Clinical and prognostic role of pressure-volume relationship in the identification of responders to cardiac resynchronization therapy	Italy	72	69	dobutamine	40 µg/kg/min	echocardiography	HR, LVEDV, LVESV, EF		12/17 high
Ciampi (2011)	Additive value of severe diastolic dysfunction and contractile reserve in the identification of responders to cardiac resynchronization therapy	Italy	69	69	dobutamine	40 µg/kg/min	echocardiography	HR, EDV, ESV, LVEF		13/17 high
Contini (2013)	Multiparametric comparison of CARvedilol, vs. NEbivolol, vs. BIsoprolol in moderate heart failure: The CARNEBI trial	Italy	61	61	bicycle ergometer	exhaustion	echocardiography	HR	Same patients	9/17 moderate

D'Andrea (2007)	Effect of dynamic myocardial dyssynchrony on mitral regurgitation during supine bicycle exercise stress echocardiography in patients with idiopathic dilated cardiomyopathy and 'narrow' QRS	Italy	60	62,3	supine ergometer	symptom-limited	echocardiography	HR, LVEDV, LVESV, LVEF, LVSV		10/17 moderate
De Groote (2015)	Sildenafil in heart transplant candidates with pulmonary hypertension	France	18	47	dobutamine	40 µg/kg/min	echocardiography	HR, CI		13/17 high
Dini (2010)	Peak power output to left ventricular mass: an index to predict ventricular pumping performance and morbidity in advanced heart failure. J Am Soc Echocardiogr, 23(12), 1259-1265.	Italy	60	61	semisupine ergometer	symptom-limited	Echocardiography	HR EDVi ESVi SV EF EO		14/19 high
Domínguez (2018)	Heart rate response and functional capacity in patients with chronic heart failure with preserved ejection fraction	Spain	74	72,5	bicycle ergometer	symptom-limited	ecg	HR		13/17 high
Donal (2012)	Heart failure with a preserved ejection fraction additive value of an exercise stress echocardiography	France	21	76	supine ergometer	submaximal	echocardiography	HR, SV, LVEF, CO		12/19 moderate

Edelmann (2011)	Neurohumorale Aktivierung und maximale Leistungsfähigkeit bei diastolischer Dysfunktion und diastolischer Herzinsuffizienz	Germany	40	67,5	supine ergometer	exhaustion	echocardiography	HR		10/17 moderate
Egstrup (2013)	Haemodynamic response during low-dose dobutamine infusion in patients with chronic systolic heart failure: comparison of echocardiographic and invasive measurements	Denmark	14	65	dobutamine	20 µg/kg/min	echocardiography	HR, CO, LVEF		10/17 moderate
Farr (2008)	Cardiopulmonary Exercise Variables in Diastolic Versus Systolic Heart Failure	USA	43	67,4	treadmill	symptom-limited	echocardiography	HR		11/17 high
Gallet (2014)	Hemodynamic effects ofIvabradine in addition to dobutamine in patients with severe systolic dysfunction	France	22	62	dobutamine	10 µg/kg/min	echocardiography	HR, EDV, ESV, SV, EF, CO		12/19 moderate
Guazzi (2016)	Right Ventricular Contractile Reserve and Pulmonary Circulation Uncoupling During Exercise Challenge in Heart Failure	Italy	97	64	bicycle ergometer	exhaustion	echocardiography	HR, EF, SVI, CO		11/17 high

Gudjonsson (2002)	Relation of "Inotropic Reserve" to Functional Capacity in Heart Failure Secondary to Ischemic or Nonischemic Cardiomyopathy	USA	35	52	treadmill	symptom-limited	echocardiograph y	HR		9/17 moderat e
Hambrecht (1995)	Physical Training in Patients With Stable Chronic Heart Failure: Effects on Cardiorespiratory Fitness and Ultrastructural Abnormalities of Leg Muscles	Germany	10	52	bicycle ergometer	symptom-limited	echocardiograph y	HR		10/19 moderat e
Hasselberg (2015)	Left ventricular global longitudinal strain is associated with exercise capacity in failing hearts with preserved and reduced ejection fraction	Norway	37	58	bicycle ergometer	exhaustion	ecg	HR		11/17 high
Henein (2013)	Impaired left ventricular systolic function reserve limits cardiac output and exercise capacity in HFpEF patients due to systemic hypertension	Sweden	17	68	bicycle ergometer	exhaustion	echocardiograph y	HR, EDV, SV, CO, EF		15/19 high
Holland (2010)	Contribution of exercise echocardiography to the diagnosis of heart failure with preserved ejection fraction (HFpEF)	Australia	148	60	treadmill	symptom-limited	echocardiograph y	HR		10/17 moderat e

Holland (2011)	Contribution of abnormal central blood pressure to left ventricular filling pressure during exercise in patients with heart failure and preserved ejection fraction	Australia	15	63	treadmill	exhaustion	ecg	HR		11/19 moderate
Hummel (2012)	Functional and Hemodynamic Cardiac Determinants of Exercise Capacity in Patients With Systolic Heart Failure	The Netherlands	102	60	treadmill	exhaustion	echocardiography	CO, CI		10/17 moderate
Iacopino (2010)	Low-Dose Dobutamine Stress Echocardiography to Assess Left Ventricular Contractile Reserve for Cardiac Resynchronization Therapy: Data From the Low-Dose Dobutamine Stress Echocardiography to Predict Cardiac Resynchronization Therapy Response (LODO-CRT) Trial	Italy	271	67	dobutamine	20 µg/kg/min	echocardiography	HR, LVEF		12/17 high
Lancellotti (2015)	Clinical Significance of Exercise Pulmonary Hypertension in Secondary Mitral Regurgitation	Belgium	159	65	bicycle ergometer	symptom-limited	ecg	HR		12/17 high

Lee (2010)	Importance of dynamic dyssynchrony in the occurrence of hypertensive heart failure with normal ejection fraction	China	47	58	dobutamine	40 µg/kg/min	echocardiography	HR, LVEF, LVEDV, LVESV		15/19 high
Madaric (2007)	Early and late effects of cardiac resynchronization therapy on exercise-induced mitral regurgitation: relationship with left ventricular dyssynchrony, remodelling and cardiopulmonary performance	The Netherlands	28	67	spiroergometry	symptom-limited	echocardiography	HR, LVEF, LVEDV, LVESV	Same Patients	10/17 moderate
Maeder (2010)	Hemodynamic Basis of Exercise Limitation in Patients With Heart Failure and Normal Ejection Fraction	Australia	14	69	supine ergometer	symptom-limited	echocardiography	HR, CI, SVI		15/19 high
Marchandise (2014)	Usefulness of Tissue Doppler Imaging to Evaluate Pulmonary. Capillary Wedge Pressure During Exercise in Patients With Reduced Left Ventricular Ejection Fraction	Belgium	40	54	bicycle ergometer	symptom-limited	echocardiography	HR, CO, CI		8/17 moderate

Marechaux (2008)	Cardiac Correlates of Exercise Induced Pulmonary Hypertension in Patients with Chronic Heart Failure Due to Left Ventricular Systolic Dysfunction	France	85	57	semisupine ergometer	symptom-limited	echocardiography	HR, LVEF		10/17 moderate
Masada (2017)	Mitral systolic velocity at peak exercise predicts impaired exercise capacity in patients with heart failure with preserved ejection fraction	Japan	50	67	supine ergometer	exhaustion	echocardiography	SV, CO, HR		10/19 moderate
McIntosh (2013)	Tissue Doppler–Derived Contractile Reserve Is a Simple and Strong Predictor of Cardiopulmonary Exercise Performance across a Range of Cardiac Diseases	UK	20	66	semisupine ergometer	exhaustion	echocardiography	LVEF, CO, HR		13/19 moderate
Meiler (1987)	An analysis of the determinants performance in congestive of exercise heart failure	USA	29	56	bicycle ergometer	exhaustion	echocardiography	HR, SVI, CI	Same patients	10/17 moderate
Meyer (2004)	Left ventricular chamber stiffness at rest as a determinant of exercise capacity in heart failure subjects with decreased ejection fraction	USA	41	54,4	treadmill	exhaustion	echocardiography	HR		11/17 high

Mezzani (2018)	Exercise gas exchange in continuous-flow left ventricular assist device recipients	Italy	15	62	bicycle ergometer	exhaustion	echocardiography	HR		11/19 moderate
Murin (2015)	Global Myocardial Contractile Reserve Assessed by High-Dose Dobutamine Stress Echocardiography Predicts Response to the Cardiac Resynchronization Therapy	Slovak Republic	52	62	dobutamine	40 µg/kg/min	echocardiography	HR, LVEDV, LVESV,LVEF		11/17 high
Nadruz (2019)	Cardiovascular phenotype and prognosis of patients with heart failure induced by cancer therapy	USA	75	48,2	spiroergometry	symptom-limited	echocardiography	HR		13/17 high
Ng (2010)	Impact of sleep disordered breathing severity on hemodynamics, autonomic balance and cardiopulmonary functional status in chronic heart failure	Australia	13	66	treadmill	symptom-limited	echocardiography	HR, SV, CO		14/17 high
Nodari (2003)	b-Blocker treatment of patients with diastolic heart failure and arterial hypertension.A prospective, randomized, comparison of the long-term effects of atenolol vs.nebivolol	Italy	13	62	bicycle ergometer	exhaustion	echocardiography	HR, CI, SVI		10/17 moderate

Nodari (2011)	Effects of n-3 Polyunsaturated Fatty Acids on Left Ventricular Function and Functional Capacity in Patients With Dilated Cardiomyopathy	Italy	67	61	bicycle ergometer	symptom-limited	echocardiograph y	HR		14/19 high
Obokata (2017	Evidence Supporting the Existence of a Distinct Obese Phenotype of Heart Failure with Preserved Ejection Fraction	USA	99	65	supine ergometer	exhaustion	echocardiograph y	HR, CI		14/19 high
Obokata (2017) 2	The Role of Diastolic Stress Testing in the Evaluation for HFpEF: A Simultaneous Invasive-Echocardiographic Study	USA	50	70	supine ergometer	submaximal	echocardiograph y	HR, CO, CI	Same Patients	12/17 high
Olson (2016)	Impaired Pulmonary Diffusion in Heart Failure with Preserved Ejection Fraction	USA	20	67	supine ergometer	submaximal	echocardiograph y	HR, SV,	Same Patients	12/19 moderat e
Pal (2015)	Effect of Selective Heart Rate Slowing in Heart Failure With Preserved Ejection Fraction	UK	22	74,6	spiroergometr y	exhaustion	ecg	HR		12/17 high

Paraskevaidis (2008)	Usefulness of Dobutamine-Induced Changes of the Two-Dimensional Longitudinal Deformation Predict Clinical and Neurohumoral Improvement in Men After Levosimendan Treatment in Acutely Decompensated Chronic Heart Failure	Greece	21	65	dobutamine	20 μg/kg/min	echocardiography	HR, EF, EDV, ESV		10/17 moderate
Paraskevaidis (2017)	Multidimensional contractile reserve predicts adverse outcome in patients with severe systolic heart failure: a 4-year follow-up study	Greece	32	66	dobutamine	20 μg/kg/min	echocardiography	HR, LVEF, LVEDV, LVESV		13/17 high
Patrianakos (2009)	Proximal aortic stiffness is related to left ventricular function and exercise capacity in patients with dilated cardiomyopathy	Greece	60	52,1	treadmill	symptom-limited	echocardiography	HR		11/19 moderate
Peeters (1996)	The 6-Minute Walk as an Appropriate Exercise Test in Elderly Patients With Chronic Heart Failure	Belgium	7	79,2	treadmill	exhaustion	echocardiography	HR	after exercise	9/19 moderate
Penicka (2010)	Heart Failure With Preserved Ejection Fraction in Outpatients With Unexplained Dyspnea	Belgium	20	67	handgrip	handgrip	echocardiography	HR, CO, EDV, ESV, EF	Same Patients	13/19 moderate

Phan (2009)	Left ventricular torsion and strain patterns in heart failure with normal ejection fraction are similar to age-related changes	UK	40	67	treadmill	exhaustion	echocardiography	HR		12/19 moderate
Phan (2009) 2	Impaired Heart Rate Recovery and Chronotropic Incompetence in Patients With Heart Failure With Preserved Ejection Fraction	UK	41	69	treadmill	exhaustion	ecg	HR		13/19 moderate
Phan (2009) 3	Heart Failure With Preserved Ejection Fraction Is Characterized by Dynamic Impairment of Active Relaxation and Contraction of the Left Ventricle on Exercise and Associated With Myocardial Energy Deficiency	UK	37	67	treadmill	symptom-limited	ecg	HR		12/19 moderate
Phan (2010)	Myocardial Contractile Inefficiency and Dyssynchrony in Heart Failure With Preserved Ejection Fraction and Narrow QRS Complex	UK	38	67	treadmill	symptom-limited	ecg	HR		12/19 moderate

Pingitore (2013)	Influence of preload and afterload on stroke volume response to low-dose dobutamine stress in patients with non-ischemic heart failure: a cardiac MR study. *Int J Cardiol, 166*(2), 475-481.	Italy	22	65	dobutamine	20 µg/kg/min	MRI	HR, SV		14/19 high
Piotrowicz (2016)	Positive Effects of the Reversion of Depression on the Sympathovagal Balance after Telerehabilitation in Heart Failure Patients	Poland	46	54,3	treadmill	symptom-limited	echocardiography	HR		15/19 high
Popovic (2018)	A flattening oxygen consumption trajectory phenotypes disease severity and poor prognosis in patients with heart failure with reduced, mid-range, and preserved ejection fraction	USA	92	65,9	bicycle ergometer	symptom-limited	ecg	HR		13/17 high
Puntawangkoon (2009)	Reduced peripheral arterial blood flow with preserved cardiac output during submaximal bicycle exercise in elderly heart failure." J Cardiovasc Magn Reson 11: 48.	USA	12	70	supine ergometer	exhaustion	MRI	HR	Same patients	15/19 high

Rengo (1995)	Comparison of the Safety and Efficacy of Delapril with Enalapril in Patients with Congestive Heart Failure	Italy	99	59,2	bicycle ergometer	exhaustion	echocardiograph y	HR		9/17 moderat e
Rommel (2016)	Extracellular Volume Fraction for Characterization of Patients With Heart Failure and Preserved Ejection Fraction	Germany	24	66,1	bicycle ergometer	exhaustion	echocardiograph y	HR		12/19 moderat e
Rubis (2009)	Usefulness of the Evaluation of Isovolumic and Ejection Phase Myocardial Signals during Stress Echocardiography in Predicting Exercise Capacity in Heart Failure Patients	Poland	30	59,3	treadmill & bicycle ergometer	exhaustion	echocardiograph y	HR, LVEF		11/17 high
Rubis (2010)	The dynamic assessment of right-ventricular function and its relation to exercise capacity in heart failure	Poland	53	58,2	semisupine ergometer	exhaustion	echocardiograph y	HR, CO, LVEF		11/17 high
Russell (1999)	Lack of Effect of Increased Inspired Oxygen Concentrations on Maximal Exercise Capacity or Ventilation in Stable Heart Failure	USA	16	56	bicycle ergometer	symptom-limited	ecg	HR		11/17 high
Sato (2015)	Comparisons of prognostic factors between young and elderly patients with chronic heart failure	Japan	123	77,8	bicycle ergometer	symptom-limited	echocardiograph y	HR		12/17 high

Scali (2017)	Exercise-induced B-lines identify worse functional and prognostic stage in heart failure patients with depressed left ventricular ejection fraction	Italy	103	64	semisupine ergometer	exhaustion	echocardiography	HR, LVEF		12/17 high
Schinkel (2002)	Assessment of residual myocardial viability in regions with chronic electrocardiographic Q-wave infarction	Netherlands	150	59	dobutamine	40 µg/kg/min	echocardiography	HR		9/17 moderate
Shafiq (2016)	Prognostic value of Cardiopulmonary Exercise Testing in Heart Failure with preserved Ejection Fraction. The Henry Ford HospITal CardioPulmonary EXercise Testing (FIT-CPX) Project	USA	126	58	treadmill	symptom-limited	ecg	HR		11/17 high
Shelton (2010)	Cardiac output does not limit submaximal exercise capacity in patients with chronic heart failure	UK	23	68,4	bicycle ergometer	submaximal	echocardiography	HR, CI		12/19 moderate
Shen (2017)	Impaired chronotropic response to physical activities in heart failure patients	China	13	52,5	treadmill	symptom-limited	ecg	HR		13/17 high
Stankovic (2014)	Dynamic relationship of left-ventricular dyssynchrony and contractile reserve in patients undergoing cardiac resynchronization therapy	Belgium	19	63	dobutamine	20 µg/kg/min	echocardiography	HR, LVEF, LVEDV, LVESV		11/17 high

Sugimoto (2017)	Left Atrial Function Dynamics During Exercise in Heart Failure	Italy	20	72,6	bicycle ergometer	symptom-limited	echocardiograph y	HR, EF, CO, SVI		15/19 high
Tabet (2000)	Comparison of the prognostic value of left ventricular filling and peak oxygen uptake in patients with systolic heart failure	France	15	51	bicycle ergometer	exhaustion	echocardiograph y	HR		11/17 high
Takata (1990)	The Role of Oxygen Extraction in Peripheral Tissues in Exercise Capacity in Chronic Heart Failure	Japan	16	59	supine ergometer	symptom-limited	echocardiograph y	HR, CI, SVI	Same Patients	9/17 moderat e
Tan (2009)	The Pathophysiology of Heart Failure With Normal Ejection Fraction	UK	56	72	bicycle ergometer	submaximal	echocardiograph y	HR, SV, CO		11/19 moderat e
Tan (2010)	Reduced left atrial function on exercise in patients with heart failure and normal ejection fraction	UK	50	72	semisupine ergometer	submaximal	echocardiograph y	HR		13/19 moderat e
Tan (2010) 2	Abnormal left ventricular function occurs on exercise in well-treated hypertensive subjects with normal resting echocardiography	UK	30	71	bicycle ergometer	submaximal	echocardiograph y	HR, SV, CO		12/19 moderat e
Tan (2013)	Exercise-induced torsional dyssynchrony relates to impaired functional capacity in patients with heart failure and normal ejection fraction	UK	67	73	semisupine ergometer	submaximal	echocardiograph y	HR, SVI, CO		14/19 high

Tartière-Kesri (2012)	Increased Proximal Arterial Stiffness and Cardiac Response With Moderate Exercise in Patients With Heart Failure and Preserved Ejection Fraction	France	23	66	bicycle ergometer	exhaustion	echocardiograph y	HR		14/19 high
Valzania (2007)	Electromechanical effects of cardiac resynchronization therapy during rest and stress in patients with heart failure	Italy	21	62	dobutamine	10 μg/kg/min	echocardiograph y	LVEDV, LVESV, LVEF, CO,HR	Same Patients	11/17 high
Van Iterson (2017)	Comparisons of Noninvasive Methods Used to Assess Exercise Stroke Volume in HFpEF	USA	18	69	bicycle ergometer	exhaustion	ecg	HR	Same Patients	15/19 high
Vigna (2000)	Neuroadrenergic Activation and Response to Dobutamine in Congestive Heart Failure Secondary to Idiopathic Dilated Cardiomyopathy	Italy	35	64	dobutamine	20 μg/kg/min	ecg	HR		9/17 moderat e
Waldenhjort (2009)	Congestive heart failure with and without atrial fibrillation – different patient populations?	Sweden	41	74	bicycle ergometer	symptom-limited	ecg	HR		9/17 moderat e
Wang (2007)	Coexistence and exercise exacerbation of intraleft ventricular contractile dyssynchrony in hypertensive patients with diastolic heart failure	Taiwan	13	70	treadmill	exhaustion	ecg	HR	postexercis e	10/17 moderat e

Wang (2014)	Changes of ventricular and peripheral performance in patients with heart failure and normal ejection fraction: insights from ergometry stress echocardiography	Hong Kong	80	65	supine ergometer	symptom-limited	echocardiography	HR		14/19 high
Wang (2015)	Left ventricular long-axis performance during exercise is an important prognosticator in patients with heart failure and preserved ejection fraction	Hong Kong	37	66	bicycle ergometer	symptom-limited	echocardiography	HR, SVI, CI		12/17 high
Wang (2016)	Importance of chronotropic response and left ventricular long-axis function for exercise performance in patients with heart failure and preserved ejection fraction	Hong Kong	20	61	bicycle ergometer	symptom-limited	echocardiography	HR, SVI, CI		10/17 moderate
Wenzelburger (2011)	Mitral annular plane systolic excursion on exercise: a simple diagnostic tool for heart failure with preserved ejection fraction	UK	62	71	semisupine ergometer	submaximal	echocardiography	HR, SV, CO		11/19 moderate
Williams (2005)	Exercise Duration and Peak Systolic Blood Pressure Are Predictive of Mortality in Ambulatory Patients with Mild-Moderate Chronic Heart Failure	UK	85	55,7	treadmill	exhaustion	echocardiography	HR		10/17 moderate

Wong (2010)	Relation of ventricular-vascular coupling to exercise capacity in ischemic cardiomyopathy: a cardiac multi-modality imaging study	USA	43	59	treadmill	symptom-limited	echocardiography	HR		11/17 high
Zaborska (2018)	Echocardiographic predictors of exercise intolerance in patients with heart failure with severely reduced ejection fraction	Poland	67	67	treadmill	symptom-limited	ecg	HR		12/17 high

Table S3 Summarized changes in haemodynamic parameters depending on intensity levels and stress type in different groups of heart failure

		**Heart rate [bpm]**		**Stroke volume [ml]**		**Cardiac output [l/min]**		**Ejection fraction [%]**	
		Mean change (95% CI)	N	Mean change (95% CI)	N	Mean change (95% CI)	N	Mean change (95% CI)	N
**All heart failure patients**									
**Light**	Dynamic	21 (0.84, 41.16)	11	40 (22.03, 57.97)	11	5.5 (3.45, 7.55)	11	N/A	-
	Dobutamine	8.9	64	9	22	0.97	64	4.65	64

		(5.13, 12.67)		(-3.23, 21.23)		(0.62, 1.32)		(2.2, 7.11)	
	Isometric	7 (-0.11, 14.11)	20	0 (-0.89, 0.89)	20	N/A	-	-5 (-8.51, -1.49)	20
**Moderate**	Dynamic	21.23 (19.69, 22.76)	487	6.02 (-0.9, 12.94)	250	1.83 (1.32, 2.33)	347	4.59 (1.08, 8.11)	51
	Dobutamine	18.3 (10.42, 26.17)	565	-0.61 (-29.02, 27.81)	46	1.65 (0.61, 2.69)	80	6.06 (3.23, 8.89)	530
**High**	Dynamic	45.69 (44.51, 46.88)	5318	13.49 (6.87, 20.1)	279	3.41 (2.86, 3.95)	795	3.69 (2.49, 4.89)	965
	Dobutamine	40.72 (33.93, 47.5)	679	N/A	-	N/A	-	11.87 (10.06, 13.67)	393
**HFrEF patients**									
**Light**	Dynamic	N/A	-	N/A	-	N/A	-	N/A	-
	Dobutamine	8.9 (4.13, 12.67)	64	9 (-3.23, 21.23)	22	0.97 (0.62, 1.32)	64	4.65 (2.2, 7.11)	64
**Moderate**	Dynamic	20.02 (13.31, 26.74)	52	N/A	-	N/A	-	N/A	-
	Dobutamine	17.47 (9.27, 25.66)	545	-0.61 (-29.02, 27.81)	46	1.74 (0.38, 3.11)	60	5.87 (2.8, 8.94)	510
**High**	Dynamic	46.61 (45.22, 48.01)	3671	12.04 (7.19, 16.9)	131	3.23 (2.56, 3.89)	597	3.79 (2.56, 5.03)	928
	Dobutamine	38.06 (30.36, 45.76)	591	N/A	-	N/A	-	12.05 (9.85, 14.24)	346
**HFpEF patients**									

**Light**	Dynamic	21 (0.84, 41.16)	11	40 (22.03, 57.97)	11	5.5 (3.45, 7.55)	11	N/A	-
	Dobutamine	N/A	-	N/A	-	N/A	-	N/A	-
	Isometric	7 (-0.11, 14.11)	20	0 (-0.89, 0.89)	20	N/A	-	-5 (-8.51, -1.49)	20
**Moderate**	Dynamic	21.29 (19.72, 22.87)	435	6.02 (-0.9, 12.94)	250	1.83 (1.32, 2.33)	347	4.59 (1.08, 8.11)	51
	Dobutamine	26 (19.26, 32.74)	20	N/A	-	1.3 (0.07, 2.53)	20	8 (2.57, 13.43)	20
**High**	Dynamic	45.02 (40.03, 50.01)	1647	14.51 (3.04, 25.97	148	3.86 (2.82, 4.89)	198	1 (-4.59, 6.59)	37
	Dobutamine	52.38 (43.56, 61.20)	88	N/A	-	N/A	-	11 (7.93, 14.07)	47

N indicates number of stress examinations included into analysis; bpm, beats per minute; ml, milliliter, l/min, liters per minute; %, percent; N/A, no data available.

Table S4 Quality assessment

Quality of all 102 studies included in this analysis was assessed by using the modified Downs and Black checklist [16]: 52 studies were found to be of high methodological quality and 50 of moderate quality. The final quality score of each study is summarized in Table S2 (see data supplements), whereas a detailed overview of 27 parameters assessing study quality and their scores are provided in Table S4. All studies stated their aims, hypothesis and main outcomes clearly. Low scores were found for external validity and selection bias, as well as for information on selection method, place and the period of participants´ recruitment, which were rarely given. Despite these drawbacks, all 102 studies were included in the current analysis. However, it should be highlighted that the scope of these meta-analyses was limited to mainly dynamic and pharmacological stress testing.

Overall, risk of bias across studies was high. The included meta-analyses therefore presented maximal bias and did not evaluate the merits of particular stress testing set ups or protocols, rather only those of heterogeneously performed dynamic stress testing in comparison to pharmacological stress testing. Within this limited scope, only 3 studies directly compared different groups of heart failure patients.

**Author**	**Publication**	**Reporting**										**External validity**			**Internal validity - bias**							**Internal validity - confounding**						**P.**
		**1**	**2**	**3**	**4**	**5**	**6**	**7**	**8**	**9**	**10**	**11**	**12**	**13**	**14**	**15**	**16**	**17**	**18**	**19**	**20**	**21**	**22**	**23**	**24**	**25**	**26**	**27**
Abinader (1999)	Effects of Low Altitude on Exercise Performance in Patients With Congestive Heart Failure After Healing of Acute Myocardial Infarction	1	1	0	1	0	1	1	0		0	0	0	1			1			1	1	0	0			0		0
Acanfora (2016)	Exercise training effects on elderly and middle-age patients with chronic heart failure after acute decompensation: A randomized, controlled trial	1	1	1	1	2	1	1	1		1	0	0	1			1			1	0	1	0			0		0
Agostoni (2010)	Relationship of resting hemoglobin concentration to peak oxygen uptake in heart failure patients	1	1	1	1		1	1	0		0	0	0	1			1			0	0	1	1					0

Agostoni (2010) 2	Effects of β-blockers on ventilation efficiency in heart failure	1	1	1	1		1	1	0		1	0	0	1			1			1	0	0	1					0
Aslanger (2015)	Effects of exercise on postexercise ventricular–arterial coupling and pulsatile efficiency in patients with systolic dysfunction	1	1	1	1	0	1	1	1		1	0	0	1			1			1	0	1	0			1		0
Bandera (2017)	Mitral regurgitation in heart failure: insights from CPET combined with exercise echocardiography	1	1	1	1		1	1	1		1	0	0	1			1			1	0	0	1					0
Bhella (2011)	Abnormal haemodynamic response to exercise in heart failure with preserved ejection fraction	1	1	1	1	0	1	1	0		1	0	0	1			1			1	0	0	0			0		0
Borlaug (2006)	Impaired Chronotropic and Vasodilator Reserves Limit Exercise Capacity in Patients With Heart Failure and a Preserved Ejection Fraction	1	1	1	1	0	1	1	0		1	0	0	1			1			1	1	0	0			0		0

Borlaug (2011)	Exercise Hemodynamics Enhance Diagnosis of Early Heart Failure with Preserved Ejection Fraction	1	1	1	1	1	1	1	0		0	0	0	1			1			0	0	0	1			0		0
Borlaug (2016)	Abnormal right ventricular-pulmonary artery coupling with exercise in heart failure with preserved ejection fraction	1	1	1	1	1	1	1	0		1	1	0	1			1			1	0	0	0			1		0
Cahalin (2013)	Heart rate recovery after the 6 min walk test rather than distance ambulated is a powerful prognostic indicator in heart failure with reduced and preserved ejection fraction: a comparison with cardiopulmonary exercise testing	1	1	1	1		1	1	1		1	0	0	1			1			1	0	0	1					0
Chattopadhyay (2008)	The effect of pharmacological stress on intraventricular dyssynchrony in left ventricular systolic dysfunction	1	1	1	1	2	1	1	0		1	0	0	1			1			1	1	0	0			1		0

Chattopadhyay (2009)	Lack of Diastolic Reserve in Patients With Heart Failure and Normal Ejection Fraction	1	1	1	1	2	1	1	0		1	1	0	1			1			1	1	0	1			0		0
Choi (2006)	Factors Associated With Decreased Cerebral Blood Flow in Congestive Heart Failure Secondary to Idiopathic Dilated Cardiomyopathy	1	1	1	1	0	1	1	0		1	0	0	1			1			1	0	0	1			0		0
Ciampi (2009)	Identification of responders to cardiac resynchronization therapy by contractile reserveduringstressechocardiography	1	1	1	1		1	1	0		1	0	0	1			1			1	1	0	1					0
Ciampi (2010)	Pressure-Volume Relationship During Dobutamine Stress Echocardiography Predicts Exercise Tolerance in Patients with Congestive Heart Failure	1	1	1	1		1	1	0		0	0	0	1			1			1	1	1	1					0

Ciampi (2010) 2	Clinical and prognostic role of pressure-volume relationship in the identification of responders to cardiac resynchronization therapy	1	1	1	1		1	1	0		1	0	0	1			1			1	1	0	1					0
Ciampi (2011)	Additive value of severe diastolic dysfunction and contractile reserve in the identification of responders to cardiac resynchronization therapy	1	1	1	1		1	1	1		1	0	0	1			1			1	0	1	1					0
Contini (2013)	Multiparametric comparison of CARvedilol, vs. NEbivolol, vs. BIsoprolol in moderate heart failure: The CARNEBI trial	1	1	1	1		1	1	0		1	0	0	1			1			0	0	0	0					0
D'Andrea (2007)	Effect of dynamic myocardial dyssynchrony on mitral regurgitation during supine bicycle exercise stress echocardiography in patients with idiopathic dilated cardiomyopathy and 'narrow' QRS	1	1	1	1		1	1	0		0	0	0	1			1			1	0	0	1					0

De Groote (2015)	Sildenafil in heart transplant candidates with pulmonary hypertension	1	1	1	1		1	1	0		1	1	0	1			1			1	0	1	1					0
Dini (2010)	Peak power output to left ventricular mass: an index to predict ventricular pumping performance and morbidity in advanced heart failure. J Am Soc Echocardiogr, 23(12), 1259-1265.	1	1	1	1	2	1	1	1		1	0	0	1			1			1	0	1	0			0		0
Domínguez (2018)	Heart rate response and functional capacity in patients with chronic heart failure with preserved ejection fraction	1	1	1	1		1	1	0		1	0	0	1			1			1	1	1	1					0
Donal (2012)	Heart failure with a preserved ejection fraction additive value of an exercise stress echocardiography	1	1	1	1	2	1	1	0		1	0	0	1			1			1	0	0	0			0		0

Edelmann (2011)	Neurohumorale Aktivierung und maximale Leistungsfähigkeit bei diastolischer Dysfunktion und diastolischer Herzinsuffizienz	1	1	1	1		1	1	0		1	0	0	1			1			1	0	0	0					0
Egstrup (2013)	Haemodynamic response during low-dose dobutamine infusion in patients with chronic systolic heart failure: comparison of echocardiographic and invasive measurements	1	1	1	1		1	1	0		1	0	0	1			1			1	0	0	0					0
Farr (2008)	Cardiopulmonary Exercise Variables in Diastolic Versus Systolic Heart Failure	1	1	1	1		1	1	0		1	0	0	1			1			1	0	0	0					1
Gallet (2014)	Hemodynamic effects ofIvabradine in addition to dobutamine in patients with severe systolic dysfunction	1	1	1	1	1	1	1	0		1	0	0	1			1			1	0	0	0			1		0

Guazzi (2016)	Right Ventricular Contractile Reserve and Pulmonary Circulation Uncoupling During Exercise Challenge in Heart Failure	1	1	1	1		1	1	0		1	0	0	1			1			1	0	0	1					0
Gudjonsson (2002)	Relation of "Inotropic Reserve" to Functional Capacity in Heart Failure Secondary to Ischemic or Nonischemic Cardiomyopathy	1	1	1	1		1	1	0		0	0	0	1			1			1	0	0	0					0
Hambrecht (1995)	Physical Training in Patients With Stable Chronic Heart Failure: Effects on Cardiorespiratory Fitness and Ultrastructural Abnormalities of Leg Muscles	1	1	1	1	0	1	1	1		0	0	0	1			1			0	1	0	0			0		0
Hasselberg (2015)	Left ventricular global longitudinal strain is associated with exercise capacity in failing hearts with preserved and reduced ejection fraction	1	1	1	1		1	1	0		1	0	0	1			1			1	0	1	0					0

Henein (2013)	Impaired left ventricular systolic function reserve limits cardiac output and exercise capacity in HFpEF patients due to systemic hypertension	1	1	1	1	2	1	1	1		0	1	0	1			1			1	1	0	0			1		0
Holland (2010)	Contribution of exercise echocardiography to the diagnosis of heart failure with preserved ejection fraction (HFpEF)	1	1	1	1		1	1	0		0	1	0	1			1			0	0	0	1					0
Holland (2011)	Contribution of abnormal central blood pressure to left ventricular filling pressure during exercise in patients with heart failure and preserved ejection fraction	1	1	1	1	2	1	1	0		0	0	0	1			1			1	0	0	0			0		0
Hummel (2012)	Functional and Hemodynamic Cardiac Determinants of Exercise Capacity in Patients With Systolic Heart Failure	1	1	1	1		1	1	0		1	0	0	1			1			0	0	1	0					0

Iacopino (2010)	Low-Dose Dobutamine Stress Echocardiography to Assess Left Ventricular Contractile Reserve for Cardiac Resynchronization Therapy: Data From the Low-Dose Dobutamine Stress Echocardiography to Predict Cardiac Resynchronization Therapy Response (LODO-CRT) Trial	1	1	1	1		1	1	1		1	0	0	1			1			0	1	1	0					0
Lancellotti (2015)	Clinical Significance of Exercise Pulmonary Hypertension in Secondary Mitral Regurgitation	1	1	1	1		1	1	0		1	0	0	1			1			1	0	1	1					0
Lee (2010)	Importance of dynamic dyssynchrony in the occurrence of hypertensive heart failure with normal ejection fraction	1	1	1	1	2	1	1	0		1	0	0	1			1			1	1	1	0			1		0

Madaric (2007)	Early and late effects of cardiac resynchronization therapy on exercise-induced mitral regurgitation: relationship with left ventricular dyssynchrony, remodelling and cardiopulmonary performance	1	1	1	1		1	1	1		1	0	0	1			1			0	0	0	0					0
Maeder (2010)	Hemodynamic Basis of Exercise Limitation in Patients With Heart Failure and Normal Ejection Fraction	1	1	1	1	2	1	1	0		1	0	0	1			1			1	1	1	1			0		0
Marchandise (2014)	Usefulness of Tissue Doppler Imaging to Evaluate Pulmonary. Capillary Wedge Pressure During Exercise in Patients With Reduced Left Ventricular Ejection Fraction	1	1	1	1		1	1	0		0	0	0	1			1			0	0	0	0					0
Marechaux (2008)	Cardiac Correlates of Exercise Induced Pulmonary Hypertension in Patients with Chronic Heart Failure Due to Left Ventricular Systolic Dysfunction	1	1	1	1		1	1	0		1	0	0	1			1			1	0	0	0					0

Masada (2017)	Mitral systolic velocity at peak exercise predicts impaired exercise capacity in patients with heart failure with preserved ejection fraction	1	1	1	1	2	1	1	0		0	0	0	1			1			0	0	0	0			0		0
McIntosh (2013)	Tissue Doppler–Derived Contractile Reserve Is a Simple and Strong Predictor of Cardiopulmonary Exercise Performance across a Range of Cardiac Diseases	1	1	1	1	2	1	1	0		1	0	0	1			1			1	0	0	0			1		0
Meiler (1987)	An analysis of the determinants performance in congestive of exercise heart failure	1	1	1	1		1	1	0		1	0	0	1			1			1	0	0	0					0
Meyer (2004)	Left ventricular chamber stiffness at rest as a determinant of exercise capacity in heart failure subjects with decreased ejection fraction	1	1	1	1		1	1	0		1	0	0	1			1			1	0	1	0					0

Mezzani (2018)	Exercise gas exchange in continuous-flow left ventricular assist device recipients	1	1	1	1	1	1	1	0		0	0	0	1			1			1	0	1	0			0		0
Murin (2015)	Global Myocardial Contractile Reserve Assessed by High-Dose Dobutamine Stress Echocardiography Predicts Response to the Cardiac Resynchronization Therapy	1	1	1	1		1	1	0		0	0	0	1			1			1	0	1	1					0
Nadruz (2019)	Cardiovascular phenotype and prognosis of patients with heart failure induced by cancer therapy	1	1	1	1		1	1	1		1	0	0	1			1			0	1	1	1					0
Ng (2010)	Impact of sleep disordered breathing severity on hemodynamics, autonomic balance and cardiopulmonary functional status in chronic heart failure	1	1	1	1		1	1	1		1	1	0	1			1			1	1	1	0					0

Nodari (2003)	b-Blocker treatment of patients with diastolic heart failure and arterial hypertension.A prospective, randomized, comparison of the long-term effects of atenolol vs.nebivolol	1	1	1	1		1	1	0		1	0	0	1			1			0	1	0	0					0
Nodari (2011)	Effects of n-3 Polyunsaturated Fatty Acids on Left Ventricular Function and Functional Capacity in Patients With Dilated Cardiomyopathy	1	1	1	1	1	1	1	1		1	0	0	1			1			1	1	0	0			1		0
Obokata (2017	Evidence Supporting the Existence of a Distinct Obese Phenotype of Heart Failure with Preserved Ejection Fraction	1	1	1	1	2	1	1	0		1	0	0	1			1			1	0	1	1			0		0
Obokata (2017) 2	The Role of Diastolic Stress Testing in the Evaluation for HFpEF: A Simultaneous Invasive-Echocardiographic Study	1	1	1	1		1	1	0		1	0	0	1			1			1	0	1	1					0
Olson (2016)	Impaired Pulmonary Diffusion in Heart Failure with Preserved Ejection Fraction	1	1	1	1	2	1	1	0		1	0	0	1			1			1	0	0	0			0		0

Pal (2015)	Effect of Selective Heart Rate Slowing in Heart Failure With Preserved Ejection Fraction	1	1	1	1		1	1	1		1	0	0	1			1			1	0	1	0					0
Paraskevaidis (2008)	Usefulness of Dobutamine-Induced Changes of the Two-Dimensional Longitudinal Deformation Predict Clinical and Neurohumoral Improvement in Men After Levosimendan Treatment in Acutely Decompensated Chronic Heart Failure	1	1	1	1		1	1	0		1	0	0	1			1			1	0	0	0					0
Paraskevaidis (2017)	Multidimensional contractile reserve predicts adverse outcome in patients with severe systolic heart failure: a 4-year follow-up study	1	1	1	1		1	1	1		1	0	0	1			1			0	1	1	1					0

Patrianakos (2009)	Proximal aortic stiffness is related to left ventricular function and exercise capacity in patients with dilated cardiomyopathy	1	1	1	1	1	1	1	0		0	0	0	1			1			1	0	1	0			0		0
Peeters (1996)	The 6-Minute Walk as an Appropriate Exercise Test in Elderly Patients With Chronic Heart Failure	1	1	1	1	0	1	1	0		0	0	0	1			1			1	0	0	0			0		0
Penicka (2010)	Heart Failure With Preserved Ejection Fraction in Outpatients With Unexplained Dyspnea	1	1	1	1	2	1	1	0		1	0	0	1			1			1	0	0	0			1		0
Phan (2009)	Left ventricular torsion and strain patterns in heart failure with normal ejection fraction are similar to age-related changes	1	1	1	1	2	1	1	0		1	0	0	1			1			1	0	0	0			0		0
Phan (2009) 2	Impaired Heart Rate Recovery and Chronotropic Incompetence in Patients With Heart Failure With Preserved Ejection Fraction	1	1	1	1	2	1	1	0		1	0	0	1			1			1	1	0	0			0		0

Phan (2009) 3	Heart Failure With Preserved Ejection Fraction Is Characterized by Dynamic Impairment of Active Relaxation and Contraction of the Left Ventricle on Exercise and Associated With Myocardial Energy Deficiency	1	1	1	1	1	1	1	0		1	0	0	1			1			0	1	1	0			0		0
Phan (2010)	Myocardial Contractile Inefficiency and Dyssynchrony in Heart Failure With Preserved Ejection Fraction and Narrow QRS Complex	1	1	1	1	2	1	1	0		1	0	0	1			1			0	0	1	0			0		0
Pingitore (2013)	Influence of preload and afterload on stroke volume response to low-dose dobutamine stress in patients with non-ischemic heart failure: a cardiac MR study. *Int J Cardiol, 166*(2), 475-481.	1	1	1	1	2	1	1	1		1	0	0	1			1			1	1	0	0			0		0

Piotrowicz (2016)	Positive Effects of the Reversion of Depression on the Sympathovagal Balance after Telerehabilitation in Heart Failure Patients	1	1	1	1	2	1	1	0		1	0	0	1			1			1	0	1	1			1		0
Popovic (2018)	A flattening oxygen consumption trajectory phenotypes disease severity and poor prognosis in patients with heart failure with reduced, mid-range, and preserved ejection fraction	1	1	1	1		1	1	1		1	0	0	1			1			1	0	1	1					0
Puntawangkoon (2009)	Reduced peripheral arterial blood flow with preserved cardiac output during submaximal bicycle exercise in elderly heart failure." J Cardiovasc Magn Reson 11: 48.	1	1	1	1	2	1	1	1		1	0	0	1			1			1	1	0	0			1		0
Rengo (1995)	Comparison of the Safety and Efficacy of Delapril with Enalapril in Patients with Congestive Heart Failure	1	1	1	1		1	1	1		0	0	0	1			1			0	0	0	0					0

Rommel (2016)	Extracellular Volume Fraction for Characterization of Patients With Heart Failure and Preserved Ejection Fraction	1	1	1	1	2	1	1	0		1	0	0	1			1			0	0	0	0			1		0
Rubis (2009)	Usefulness of the Evaluation of Isovolumic and Ejection Phase Myocardial Signals during Stress Echocardiography in Predicting Exercise Capacity in Heart Failure Patients	1	1	1	1		1	1	0		1	0	0	1			1			1	0	1	0					0
Rubis (2010)	The dynamic assessment of right-ventricular function and its relation to exercise capacity in heart failure	1	1	1	1		1	1	0		1	0	0	1			1			1	0	1	0					0
Russell (1999)	Lack of Effect of Increased Inspired Oxygen Concentrations on Maximal Exercise Capacity or Ventilation in Stable Heart Failure	1	1	1	1		1	1	1		1	0	0	1			1			1	0	0	0					0

Sato (2015)	Comparisons of prognostic factors between young and elderly patients with chronic heart failure	1	1	1	1		1	1	0		1	0	0	1			1			1	0	1	1					0
Scali (2017)	Exercise-induced B-lines identify worse functional and prognostic stage in heart failure patients with depressed left ventricular ejection fraction	1	1	1	1		1	1	1		0	0	0	1			1			1	0	1	1					0
Schinkel (2002)	Assessment of residual myocardial viability in regions with chronic electrocardiographic Q-wave infarction	1	1	1	1		1	1	0		0	0	0	1			1			1	0	0	0					0
Shafiq (2016)	Prognostic value of Cardiopulmonary Exercise Testing in Heart Failure with preserved Ejection Fraction. The Henry Ford HospITal CardioPulmonary EXercise Testing (FIT-CPX) Project	1	1	1	1		1	1	0		1	0	0	1			1			0	0	1	1					0

Shelton (2010)	Cardiac output does not limit submaximal exercise capacity in patients with chronic heart failure	1	1	1	1	1	1	1	0		1	0	0	1			1			0	1	1	0			0		0
Shen (2017)	Impaired chronotropic response to physical activities in heart failure patients	1	1	1	1		1	1	1		1	0	0	1			1			1	1	0	1					0
Stankovic (2014)	Dynamic relationship of left-ventricular dyssynchrony and contractile reserve in patients undergoing cardiac resynchronization therapy	1	1	1	1		1	1	1		1	0	0	1			1			1	0	0	0					0
Sugimoto (2017)	Left Atrial Function Dynamics During Exercise in Heart Failure	1	1	1	1	2	1	1	0		1	0	0	1			1			0	0	1	1			1		1
Tabet (2000)	Comparison of the prognostic value of left ventricular filling and peak oxygen uptake in patients with systolic heart failure	1	1	1	1		1	1	1		1	0	0	1			1			0	0	0	0					0

Takata (1990)	The Role of Oxygen Extraction in Peripheral Tissues in Exercise Capacity in Chronic Heart Failure	1	1	1	1		1	1	0		0	0	0	1			1			1	0	0	0					0
Tan (2009)	The Pathophysiology of Heart Failure With Normal Ejection Fraction	1	1	1	1	2	1	1	0		1	0	0	1			1			0	0	0	0			0		0
Tan (2010)	Reduced left atrial function on exercise in patients with heart failure and normal ejection fraction	1	1	1	1	2	1	1	1		1	0	0	1			1			1	0	0	0			0		0
Tan (2010) 2	Abnormal left ventricular function occurs on exercise in well-treated hypertensive subjects with normal resting echocardiography	1	1	1	1	2	1	1	1		1	0	0	1			1			0	0	0	0			0		0
Tan (2013)	Exercise-induced torsional dyssynchrony relates to impaired functional capacity in patients with heart failure and normal ejection fraction	1	1	1	1	2	1	1	0		1	1	0	1			1			0	1	1	0			0		0

Tartière-Kesri (2012)	Increased Proximal Arterial Stiffness and Cardiac Response With Moderate Exercise in Patients With Heart Failure and Preserved Ejection Fraction	1	1	1	1	2	1	1	0		1	0	0	1			1			1	1	1	0			0		0
Valzania (2007)	Electromechanical effects of cardiac resynchronization therapy during rest and stress in patients with heart failure	1	1	1	1		1	1	0		1	0	0	1			1			1	1	0	0					0
Van Iterson (2017)	Comparisons of Noninvasive Methods Used to Assess Exercise Stroke Volume in HFpEF	1	1	1	1	2	1	1	1		1	1	0	1			1			1	0	0	0			1		0
Vigna (2000)	Neuroadrenergic Activation and Response to Dobutamine in Congestive Heart Failure Secondary to Idiopathic Dilated Cardiomyopathy	1	1	1	1		1	1	0		0	0	0	1			1			0	0	0	1					0
Waldenhjort (2009)	Congestive heart failure with and without atrial fibrillation – different patient populations?	1	1	1	1		1	1	0		0	0	0	1			1			1	0	0	0					0

Wang (2007)	Coexistence and exercise exacerbation of intraleft ventricular contractile dyssynchrony in hypertensive patients with diastolic heart failure	1	1	1	1		1	1	0		1	0	0	1			1			1	0	0	0					0
Wang (2014)	Changes of ventricular and peripheral performance in patients with heart failure and normal ejection fraction: insights from ergometry stress echocardiography	1	1	1	1	2	1	1	0		1	0	0	1			1			0	0	1	0			1		1
Wang (2015)	Left ventricular long-axis performance during exercise is an important prognosticator in patients with heart failure and preserved ejection fraction	1	1	1	1		1	1	1		1	0	0	1			1			0	0	1	1					0

Wang (2016)	Importance of chronotropic response and left ventricular long-axis function for exercise performance in patients with heart failure and preserved ejection fraction	1	1	1	1		1	1	0		1	0	0	1			1			0	0	1	0					0
Wenzelburger (2011)	Mitral annular plane systolic excursion on exercise: a simple diagnostic tool for heart failure with preserved ejection fraction	1	1	1	1	2	1	1	0		1	0	0	1			1			0	0	0	0			0		0
Williams (2005)	Exercise Duration and Peak Systolic Blood Pressure Are Predictive of Mortality in Ambulatory Patients with Mild-Moderate Chronic Heart Failure	1	1	1	1		1	1	0		1	0	0	1			1			1	0	0	0					0
Wong (2010)	Relation of ventricular-vascular coupling to exercise capacity in ischemic cardiomyopathy: a cardiac multi-modality imaging study	1	1	1	1		1	1	0		1	0	0	1			1			1	0	0	1					0

Zaborska (2018)	Echocardiographic predictors of exercise intolerance in patients with heart failure with severely reduced ejection fraction	1	1	1	1		1	1	0		1	0	0	1			1			1	0	1	1					0

Reporting: 1. Hypothesis/aims/objectives clearly stated **2.** Main outcome measures clearly described **3.** Characteristics of patients/subjects clearly described **4.** Interventions of interest clearly described **5.** Distribution of principal confounders in each group clearly described **6.** Main findings clearly described **7.** Estimates of random variability in the data provided **8.** Important adverse events reported **9.** Characteristics of patients lost to follow-up described **10.** Actual probability values reported External validity: **11.** Participants approached representative of entire population **12.** Participants recruited representative of entire population **13.** Staff, places and facilities representative of majority of population Internal validity-bias: **14.** Blinding of study subjects **15.** Blinding of assessors **16.** Data based on data-dredging clearly stated **17.** Adjustment of different length of follow-up or duration between case and control **18.** Appropriate statistical tests used **19.** Compliance to intervention reliable **20.** Main outcome measure reliable and valid Internal validity-confounding: **21.** Intervention groups or case-controls recruited from same population **22.** Intervention groups or case-controls recruited at the same time **23.** Study subjects randomized to the interventions **24.** Was concealed randomization to allocation undertaken **25.** Adequate adjustment made in the analysis of confounders **26.** Patient losses accounted for Power (P.): **27.** Sufficiently powered cohort size

Table S5 Haemodynamic changes (weighted by study participants) across all patients with heart failure

	Dynamic exercise		Pharmacological stress		Kruskal-Wallis-Test
		Study arms reporting variable (N of tests)		Study arms reporting variable (N of tests)	P-Value
Total N of stress tests		114 (5920)		25 (1308)	
Age, years	65 (60 – 69)	114 (5920)	65 (62 – 68)	25 (1308)	0.832
Male, %	68.4 (40 – 82.1)	114 (5920)	76.7 (69.4 – 83.3)	25 (1308)	0.054
BSA, m2	1.87 (1.71 – 1.99)	23 (1011)	2.03 (2.03 – 2.03)	1 (20)	0.134
BMI,kg/m2	30 (27.8 – 32)	59 (2714)	26.5 (26.1 – 27.1)	6 (204)	0.063
Resting HR,bpm	70.8 (68 – 77)	113 (5818)	72 (70 – 76)	25 (1308)	0.433
Resting SV, ml	69.35 (65 – 74.1)	16 (542)	62 (44 – 82)	3 (68)	0.402
Resting CO, l/min	4.6 (3.85 – 5.1)	28 (1155)	3.9 (3.1 – 4.4)	7 (144)	0.084
Resting EF, %	36 (31 – 62)	105 (5640)	29 (26 – 33)	25 (1308)	0.001
Light intensity, %	1	1 (11)	12	3 (64)	
Moderate intensity, %	11	13 (487)	44	11 (565)	
High intensity, %	88	100 (5422)	44	11 (679)	

Values are reported as median (interquartile range). BMI indicates body mass index; BSA, body surface area; HR, heart rate; SV, stroke volume; CO, cardiac output; EF, ejection fraction.

Table S6: Haemodynamic changes (weighted by study participants) in HFrEF patients

	Dynamic exercise		Pharmacological stress		Kruskal-Wallis-Test
		Study arms reporting variable (N of tests)		Study arms reporting variable (N of tests)	P-Value
Total N of stress tests		64 (3827)		22 (1200)	
Age, years	61.75 (56 – 65.15)	64 (3827)	64.5 (62 – 68)	22 (1200)	0.008
Male, %	80.7 (73 – 93.8)	64 (3827)	79.1 (72.5 – 83.3)	22 (1200)	0.517
BSA, m2	1.89 (1.86 – 1.94)	8 (399)			
BMI,kg/m2	27.6 (26.54 – 28.6)	19 (1021)	26.25 (26.05 – 26.75)	4 (143)	0.074
Resting HR,bpm	71.7 (68.5 – 79)	63 (3725)	72 (70 – 77)	22 (1200)	0.964
Resting SV, ml	67.7 (55 – 102)	3 (133)	62 (44 – 82)	3 (68)	0.513
Resting CO, l/min	3.9 (3.6 – 4.3)	13(599)	3.65 (3.1 – 4)	6 (124)	0.204
Resting EF, %	31.55 (28 – 35)	62 (3758)	28 (26 – 30)	22 (1200)	0.01
Light intensity, %			14	3 (64)	
Moderate intensity, %	3	2 (52)	45	10 (545)	
High intensity, %	97	62 (3775)	41	9 (591)	

Values are reported as median (interquartile range). BMI indicates body mass index; BSA, body surface area; HR, heart rate; SV, stroke volume; CO, cardiac output; EF, ejection fraction.

Table S7: Haemodynamic changes (weighted by study participants) in HFpEF patients

	Dynamic exercise		Pharmacological stress		Kruskal-Wallis-Test
		Study arms reporting variable (N of tests)		Study arms reporting variable (N of tests)	P-Value
Total N of stress tests		50 (2093)		3 (108)	
Age, years	67.45 (65 – 70)	50 (2093)	67 (58 – 70)	3 (108)	0.756
Male, %	37.5 (28.9 – 53.5)	50 (2093)	39 (25 – 44.7)	3 (108)	0.38
BSA, m2	1.82 (1.69 – 1.99)	15 (612)	2.03 (2.03 – 2.03)	1 (20)	0.202
BMI,kg/m2	31 (29.8 – 33.8)	40 (1693)	29 (26.6 – 31.4)	2 (61)	0.703
Resting HR,bpm	69 (68 – 75)	50 (2093)	69 (67 – 76)	3 (108)	0.568
Resting SV, ml	71 (65 – 74.1)	13 (409)			
Resting CO, l/min	5.1 (4.8 – 5.1)	15 (556)	4.9 (4.9 – 4.9)	1 (20)	0.67
Resting EF, %	62 (60 – 63)	47 (2010)	62 (60 – 67)	3 (108)	0.689
Light intensity, %	2	1 (11)			
Moderate intensity, %	22	11 (435)	33	1 (20)	
High intensity, %	76	38 (1647)	67	2 (88)	

Values are reported as median (interquartile range). BMI indicates body mass index; BSA, body surface area; HR, heart rate; SV, stroke volume; CO, cardiac output; EF, ejection fraction.

Table S8. Multivariable meta-regression analysis assessing each variable's impact on HR changes, number of observations: 138, weighted by the number of participants in each of the studies

	Coef.	Std. Err.	t	P>t	[95% Conf.	Interval]
**Intervention type**	-4.136818	2.712053	-1.53	0.13	-9.501906	1.228269
**Moderate intensity**	9.635064	6.028653	1.6	0.112	-2.291049	21.56118
**High intensity**	31.19496	5.916508	5.27	<0.001	19.4907	42.89922
**Age [years]**	-0.5480376	0.1700176	-3.22	0.002	-0.8843729	-0.2117023
**Heart failure group**	2.957683	2.179663	1.36	0.177	-1.354211	7.269576

Supplement to the Results section

Pooled effects of rest-stress changes from single arm studies for HFrEF

Pooled effects, sample size and uncertainty are summarized in Figure S3. Low dose infusion of dobutamine (5 - 10μg/kg/min) resulted in changes of 8.9 bpm (95% CI, 5.13 to 12.67; I^2=0.0%), 9ml (95% CI, -3.23 to 21.23; reported in one study), 0.97 l/min (95% CI, 0.62 to 1.32; I^2=0,0%), 4.65% (95% CI, 2.2 to 7.11 I^2=0.0%) for HR, SV, CO and EF respectively. No studies analysing light dynamic exercise testing and changes in in HR, SV, CO and EF were available.

In the moderate intensity group of dynamic stress testing pooled estimates of changes in HR were 20.02 bpm (95% CI, 13.31 to 26.74; I^2=0.0). There were no studies available investigating changes in SV, CO and EF with a moderate level of dynamic exercise. For moderate dose of dobutamine infusion (11 - 20μg/kg/min) changes in HR were 17.4 bpm (95% CI, 9.27 to 25.66; I^2=97%), in SV -0.61ml (95% CI, -29.02 to 27.81; I^2=88.8%), in CO 1.74l/min (95% CI, 0.38 to 3.11; I^2=79.3%) and in EF 5.87% (95% CI, 2.8 to 8.94; I^2=84.4%).

A high level of dynamic exercise increased HR by 46.61 bpm (95% CI, 45.22 to 48.01; I^2=98.8%), SV by 12.04 ml (95% CI, 7.19 to 16.90; I^2=0.0%), CO by 3.23 l/min (95% CI, 2.56 to 3.89; I^2=87.9%) and EF by 3.79% (95% CI, 2.56 to 5.03; I^2=55.6%). For high dosage of dobutamine (21 - 40μg/kg/min) HR increased by 38.06 bpm (95% CI, 30.36 to 45.76; I^2=93.1%) and EF increased by 12.05% (95% CI, 9.85 to 14.24; I^2=53.7%). There were no studies available which performed high dose pharmacological stress testing in HFrEF individuals listing changes in SV and CO.

Pooled effects of rest-stress changes from single arm studies for HFpEF

Pooled effects, sample size and uncertainty are summarized in Figure S4. Low intensity dynamic exercise increased HR by 21 bpm (95% CI, 0.84 to 41.16; reported in one study), SV by 40 ml (95% CI, 22.03 to 57.97; reported in one study), CO by 5.5 l/min (95% CI, 3.45 to 7.55; reported in one study) compared to resting baseline values. No study analysed changes in EF with low dynamic exercise testing and there was also no study that investigated changes in HR, SV, CO and EF with low dose

of dobutamine infusions. Mean absolute changes of isometric exercise were 7 bpm (95% CI, -0.11 to 14.11; reported in one study), 0 l/min (95% CI, -0.89 to 0.89; reported in one study) and -5% (95% CI, -8.51 to -1.49; reported in one study) for HR, CO and EF respectively.

In the moderate-intensity group for dynamic stress testing pooled estimates of changes in HR were 21.29 bpm (95% CI, 19.72 to 22.87; I^2=0.0), in SV 6.02 ml (95% CI, -0,9 to 12.94; I^2=67.0%), in CO 1.83 l/min (95% CI, 1.32 to 2.33; I^2=66.6%) and in EF 4.59% (95% CI, 1.08 to 8.11; I^2=0.0%). For moderate dosage of dobutamine (11 - 20µg/kg/min) changes in HR were 26 bpm (95% CI, 19.26 to 32.74; reported in one study), in CO 1.3 l/min (95% CI, 0.07 to 2.53; reported in one study) and in EF 8% (95% CI, 2.57 to 13.43; reported in one study). There was no study available analysing changes in SV with moderate pharmacological testing.

High dynamic exercise increased HR by 45.02 bpm (95% CI, 40.03 to 50.01; I^2=95.8%), SV by 14.51 ml (95% CI, 3.04 to 25.97; I^2=80.6%), CO by 3.86 l/min (95% CI, 2.82 to 4.89; I^2=84.0%) and EF by 1% (95% CI, -4.59 to 6.59; I^2=0.0%). For high dose dobutamine infusion (11 - 20µg/kg/min) changes in HR were 52.38 bpm (95% CI, 43.56 to 61.20; I^2=74.8%) and in EF 11% (95% CI, 7.93 to 14.07; reported in one study). No study investigated changes in SV and CO with high dose pharmacological exercise testing.

Heterogeneity assessment

Between-study heterogeneity was found for HFrEF patients for HR change in the high intensity dynamic as well as the moderate and high dose pharmacological group. This was also the case for SV changes in the moderate dose pharmacological group, for CO change in the high dynamic and moderate pharmacological group and for EF in the moderate pharmacological group. For HFpEF patients, heterogeneity was found for change of HR, SV and CO in the high-intensity dynamic group. For both HF groups, heterogeneity was found for HR change in the moderate pharmacological and in the high dynamic and pharmacological group. This was also the case for SV in the moderate pharmacological group and for CO change in the high dynamic group. For changes of EF, heterogeneity was found in the moderate pharmacological group. Medium heterogeneity was found amongst HFrEF patients for EF change in the high dynamic (I^2=55.6%, p=0.001) and in the high pharmacological group (I^2=53.7%, p=0.071). For HFpEF patients, medium heterogeneity was found for HR change in the high dose pharmacological group (I^2=74.8%, p=0.046) and for change of SV and CO in the moderate-intensity dynamic group (I^2=67%, p=0.006; I^2=66.6%, p=0.004). For both groups medium heterogeneity was found for SV change in the moderate and high dynamic group (I^2=67%, p=0.006; I^2=68.5%, p=0.002), for CO change in the moderate dynamic and

moderate pharmacological group (I^2=66.6%, *p*=0.004; I^2=71.3%, *p*=0.015) and for EF change in the high dynamic group (I^2=52.9%, *p*=0.001). Lower heterogeneity was found for both HF groups for EF change in the high dose pharmacological group (I^2=44.7%, *p*=0.108). No heterogeneity was found for HFrEF patients for HR change in the low and moderate dynamic group, nor for SV change in the high dynamic group, or for change of CO and EF in the low dose pharmacological group. A visual representation of heterogeneity is available within the supplemental materials as forest plots (S5- S31).

Supplementary Figures

Figure S1

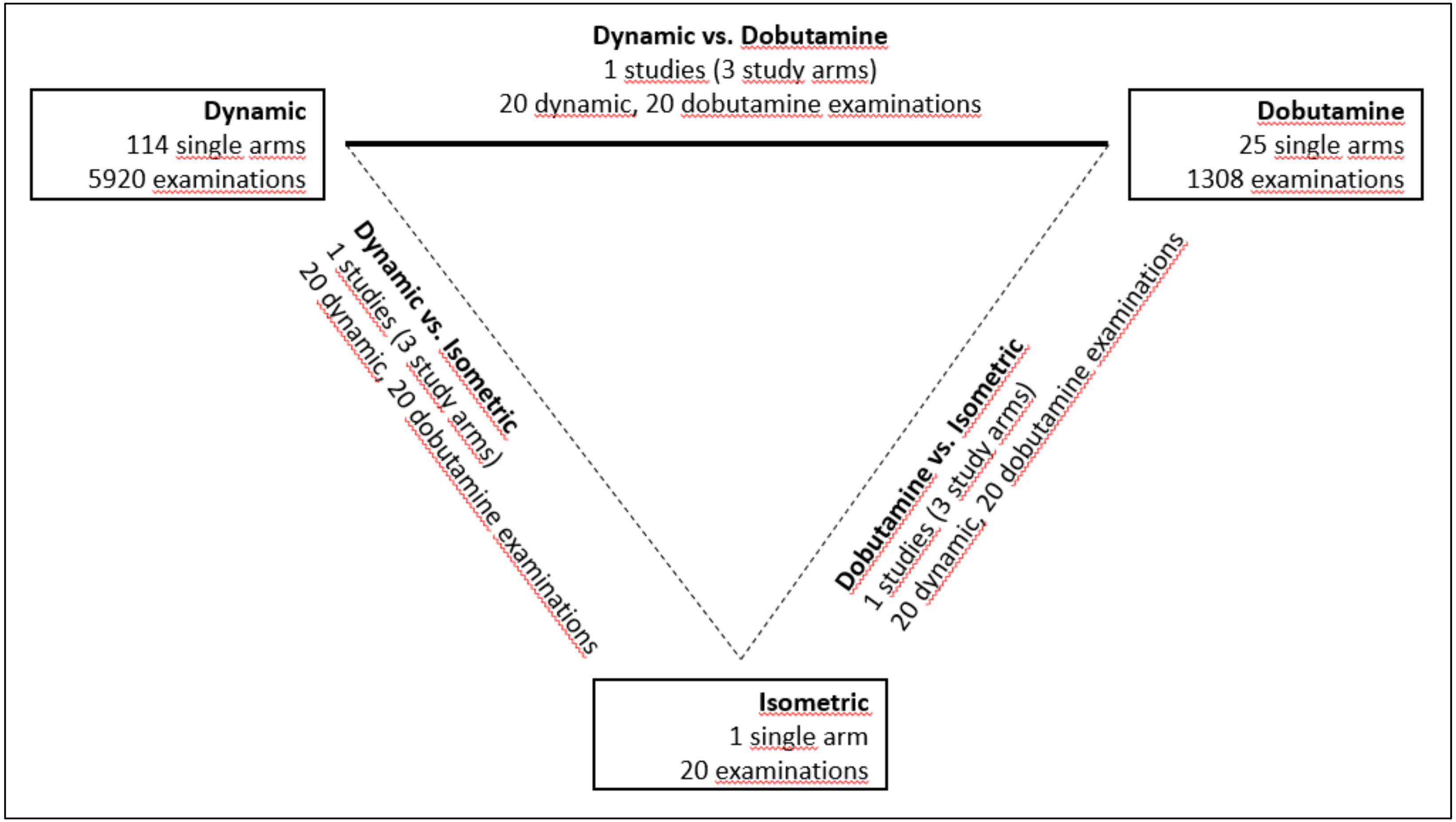

Figure S1. Network of evidence for findings in heart failure subjects (adapted from Salcher et al., Circ Cardiovasc Interv. 2016; 9). Different types of stress testing (dynamic, isometric, pharmacological stress) are shown at the edges. Lines connecting the edges represent the limited number of studies directly comparing the different stress types to each other

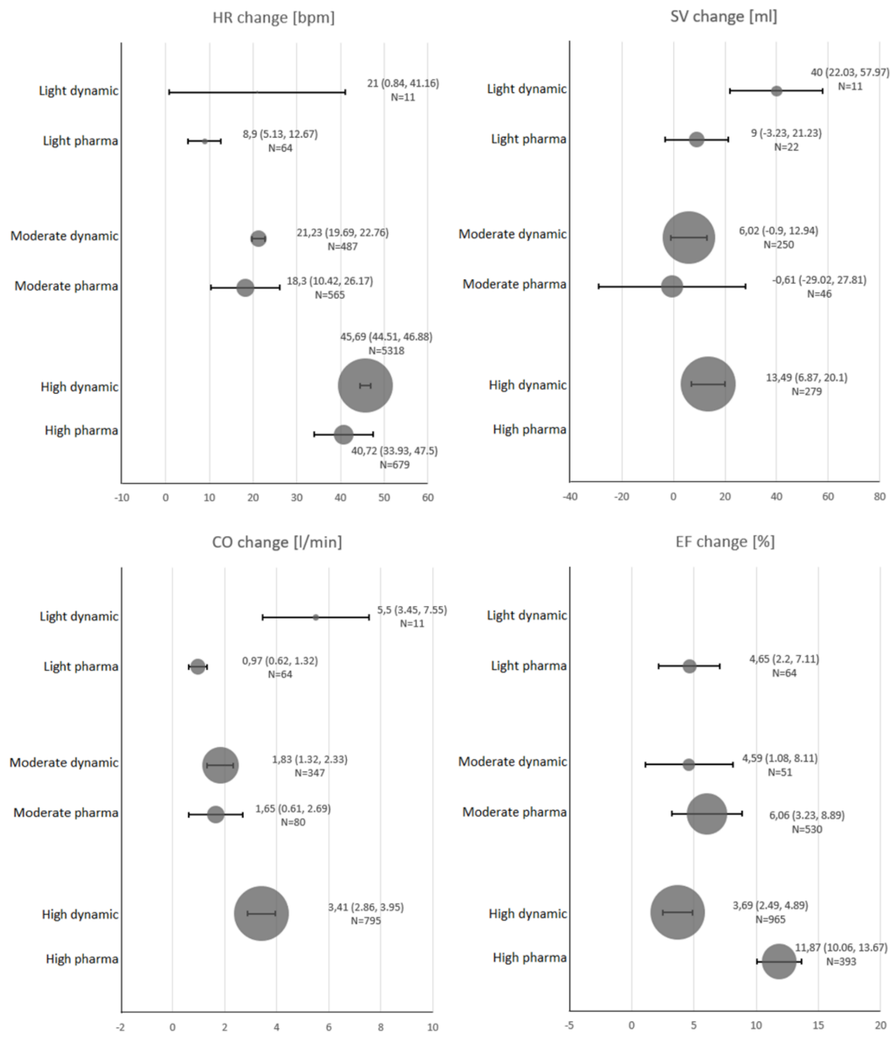

Figure S2. Changes in haemodynamic parameters in heart failure patients across different levels and types of stress testing

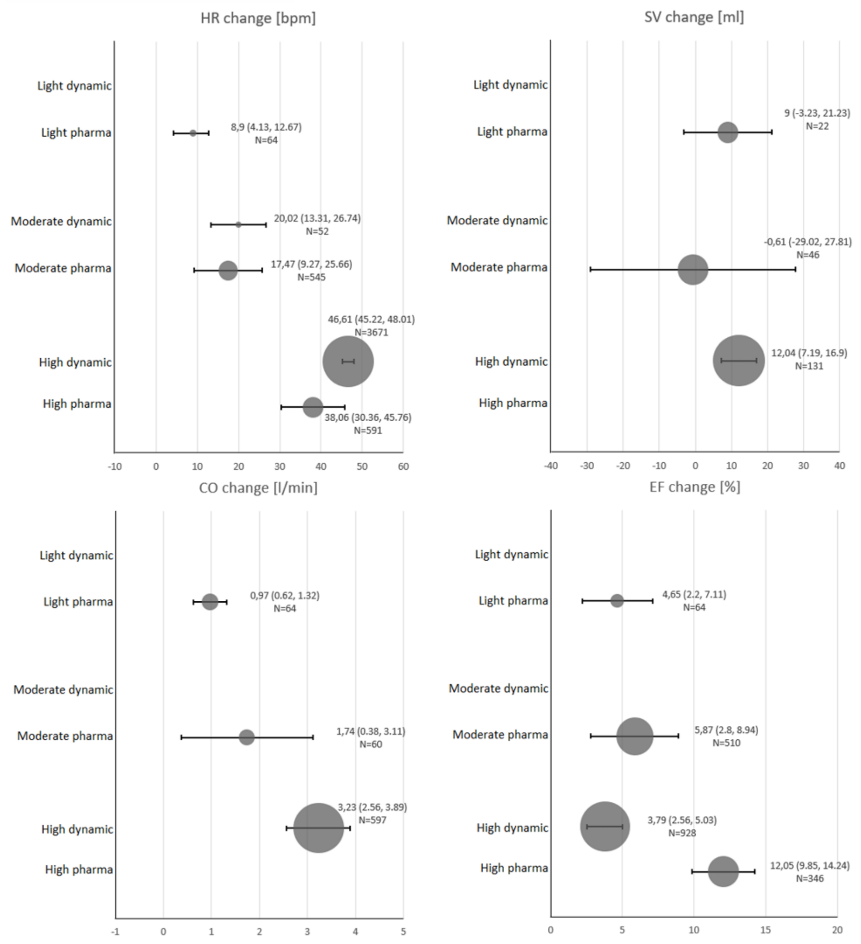

Figure S3. Changes in haemodynamic parameters in heart failure patients with reduced ejection fraction (HFrEF) across different levels and types of stress testing

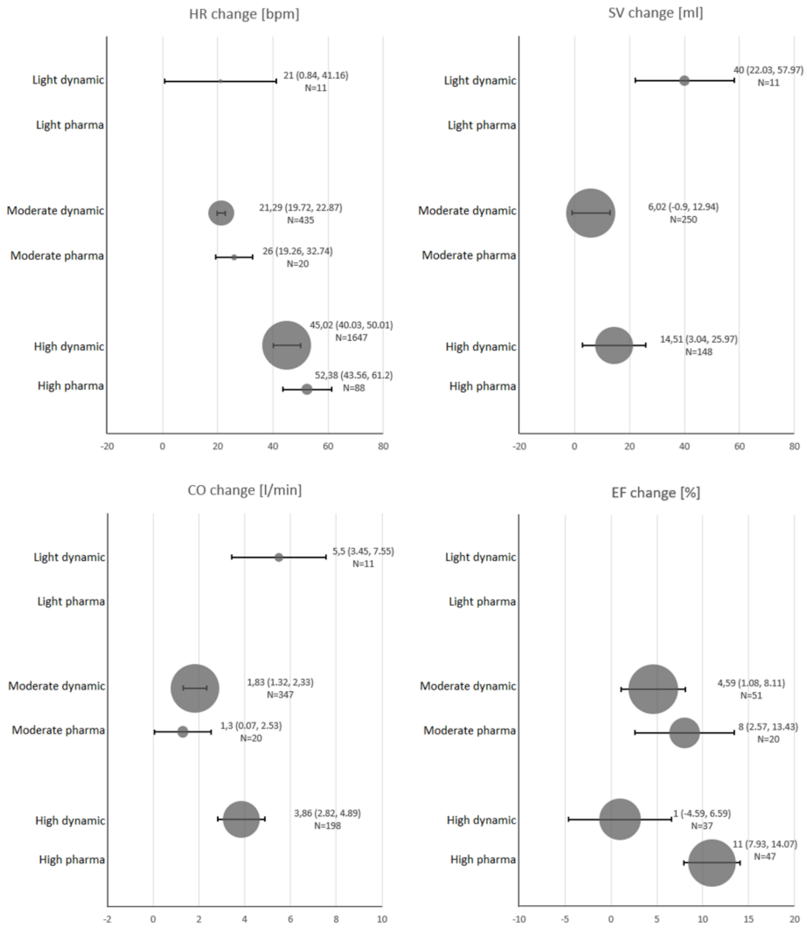

Figure S4. **Changes in haemodynamic parameters in heart failure patients with preserved ejection fraction (HFpEF) across different levels and types of stress testing**

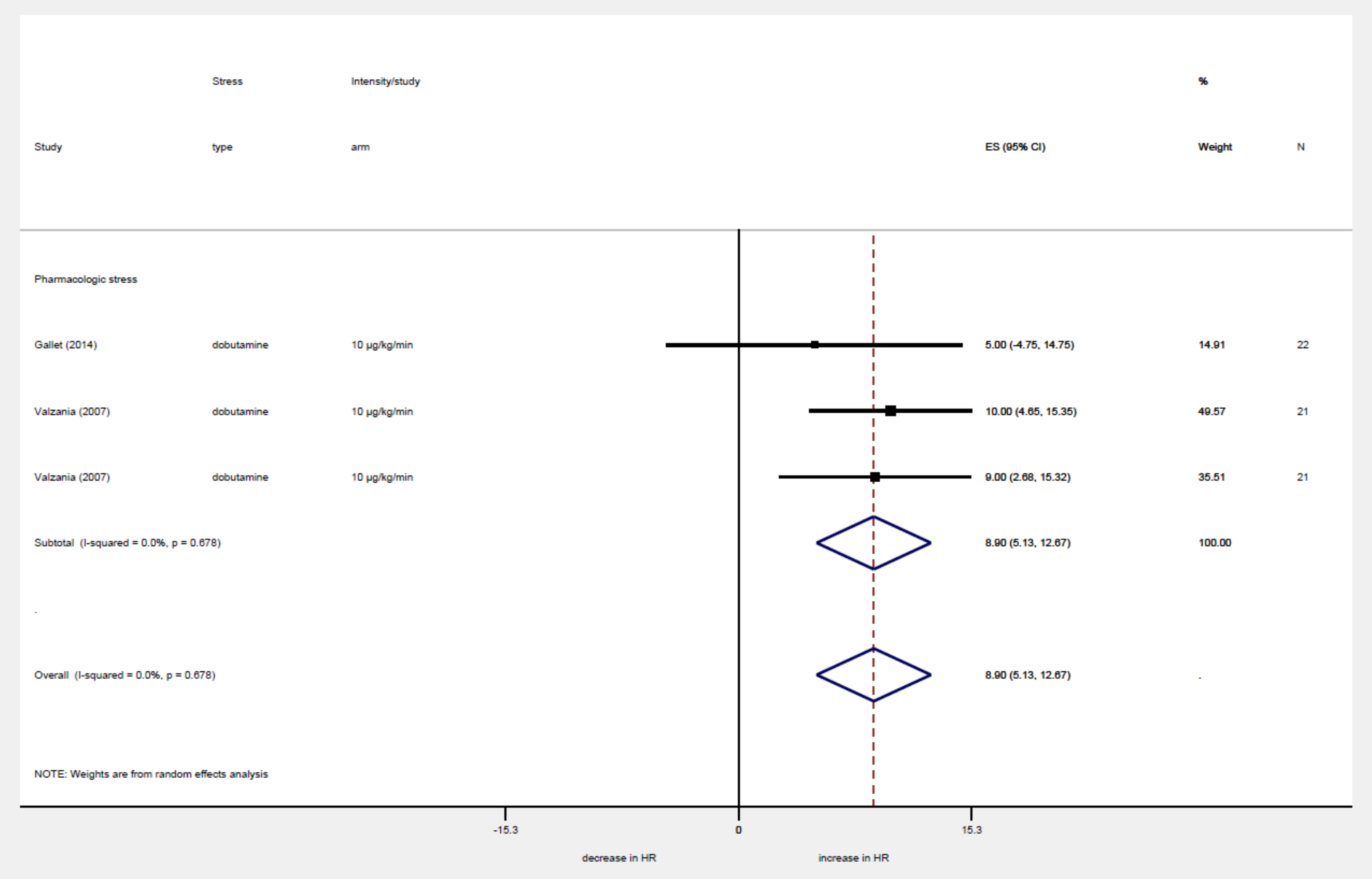

Figure S5. HR changes [bpm] in heart failure patients for light intensity pharmacological stress.

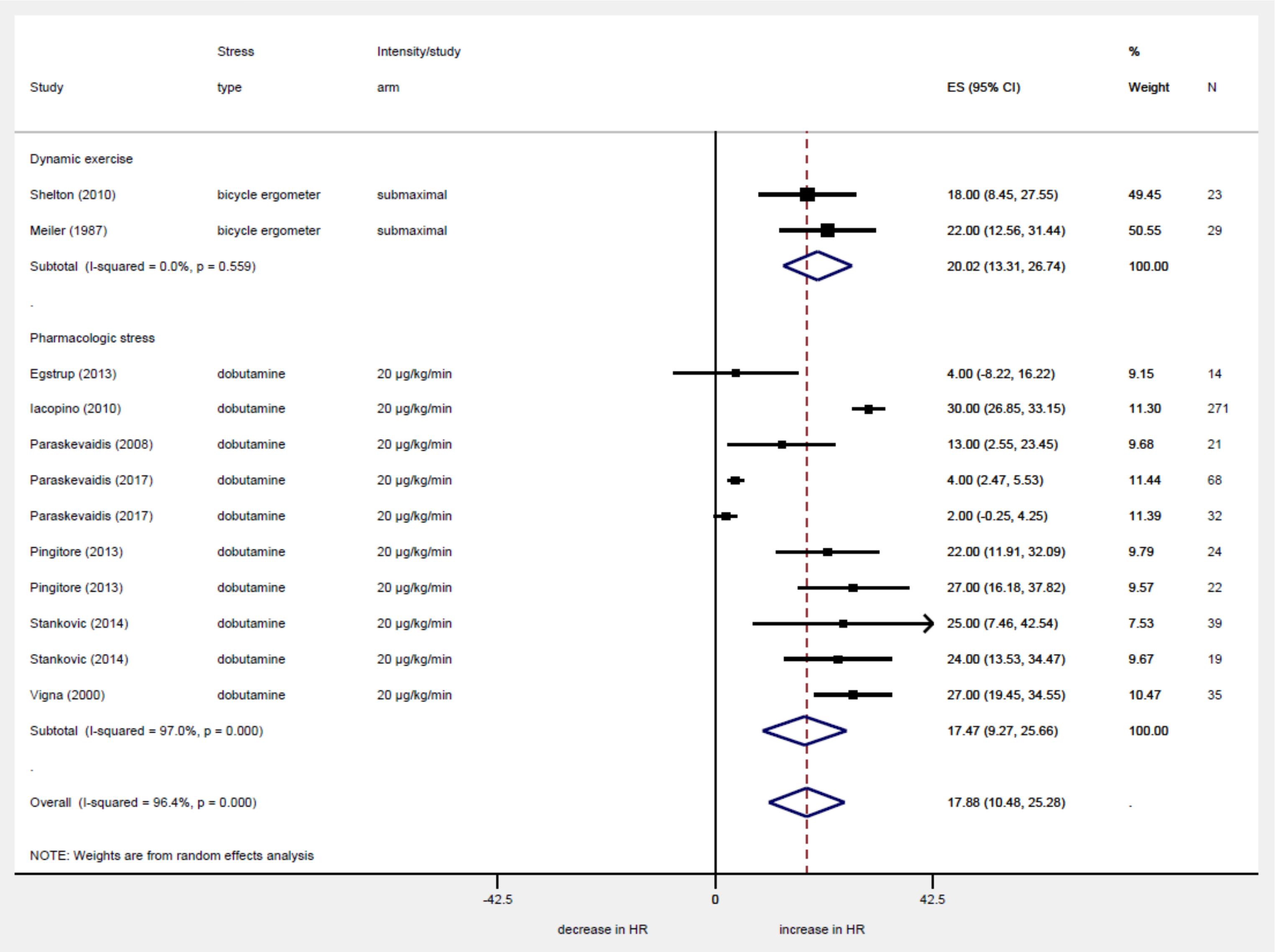

Figure S6. HR Changes [bpm] in heart failure patients for moderate intensity stress.

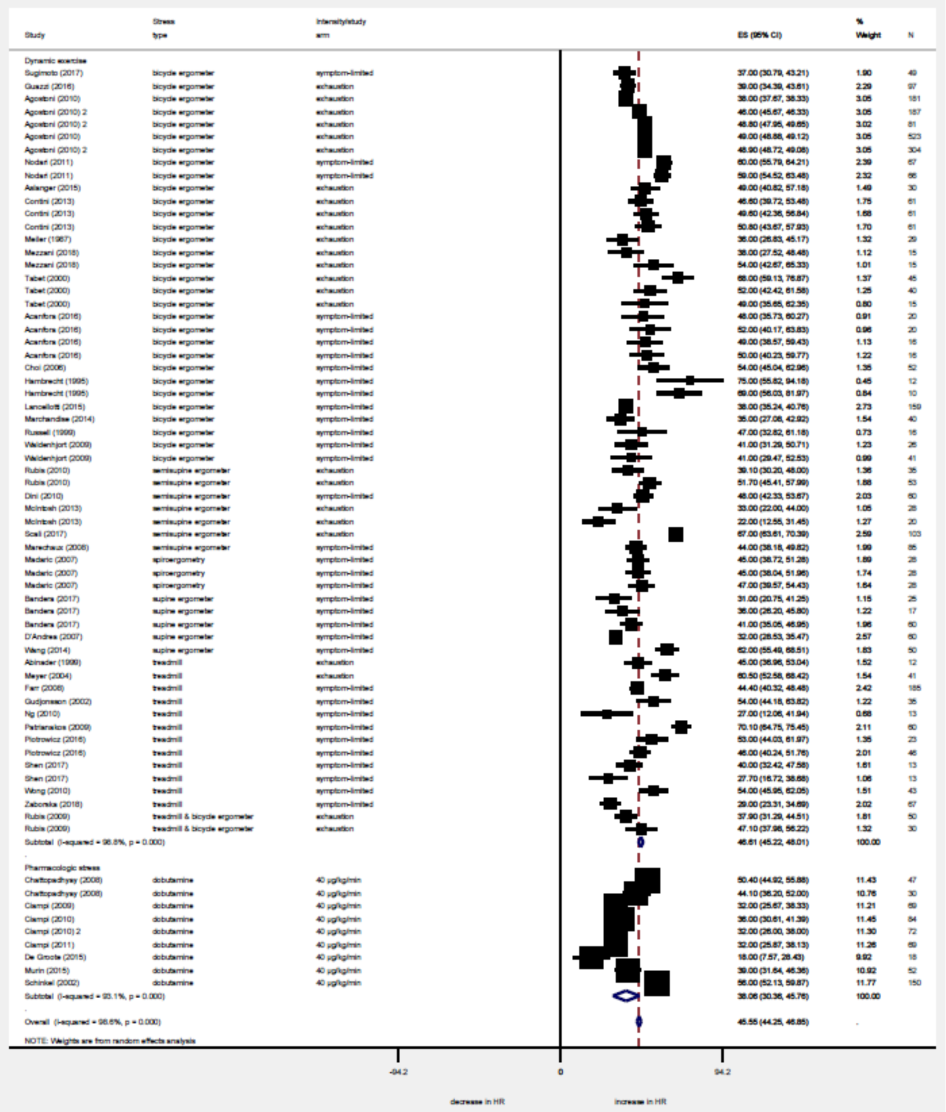

Figure S7. HR Changes [bpm] in heart failure patients for high intensity stress.

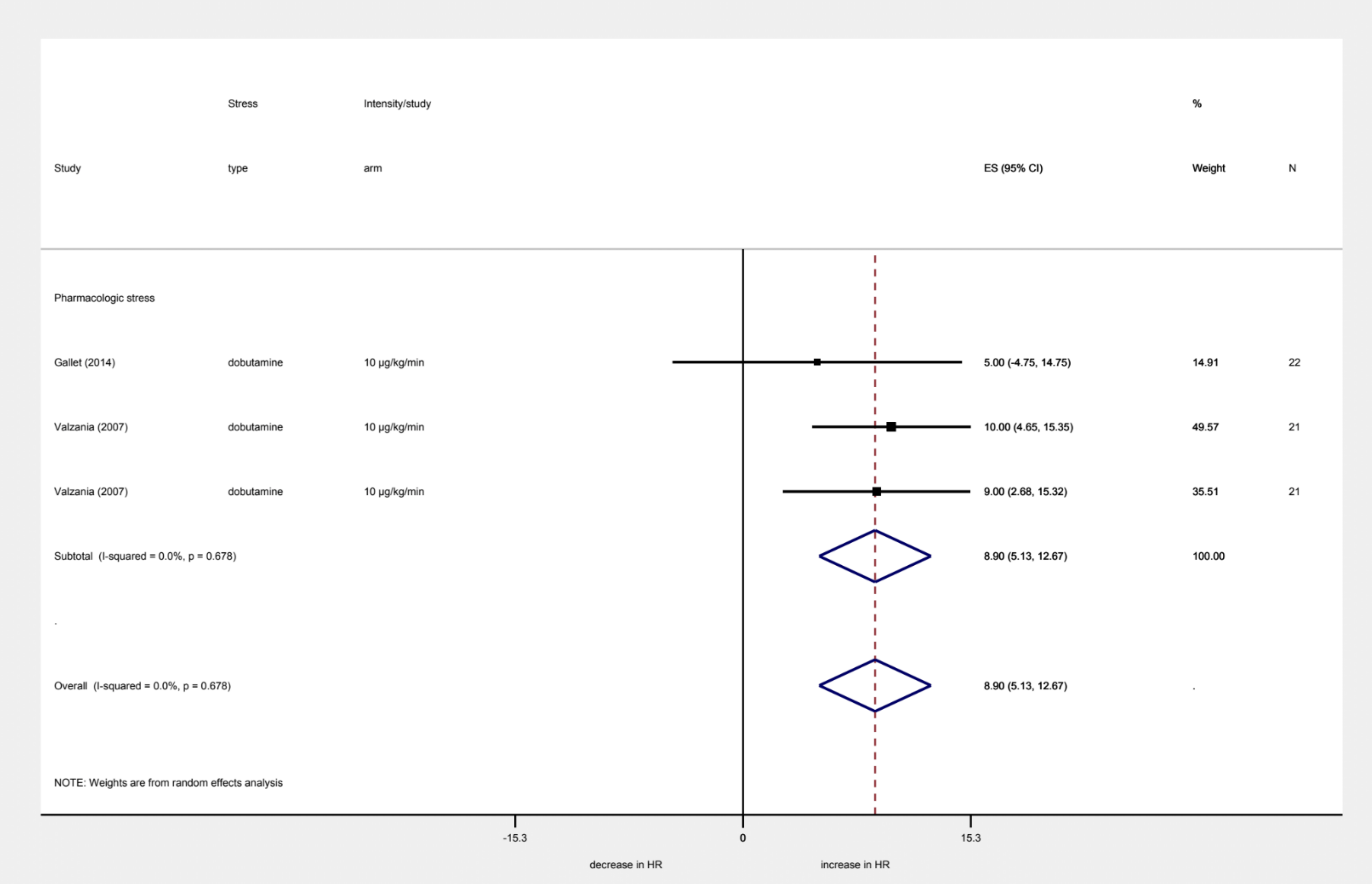

Figure S8. HR Changes [%] in heart failure patients with reduced ejection fraction for light intensity stress.

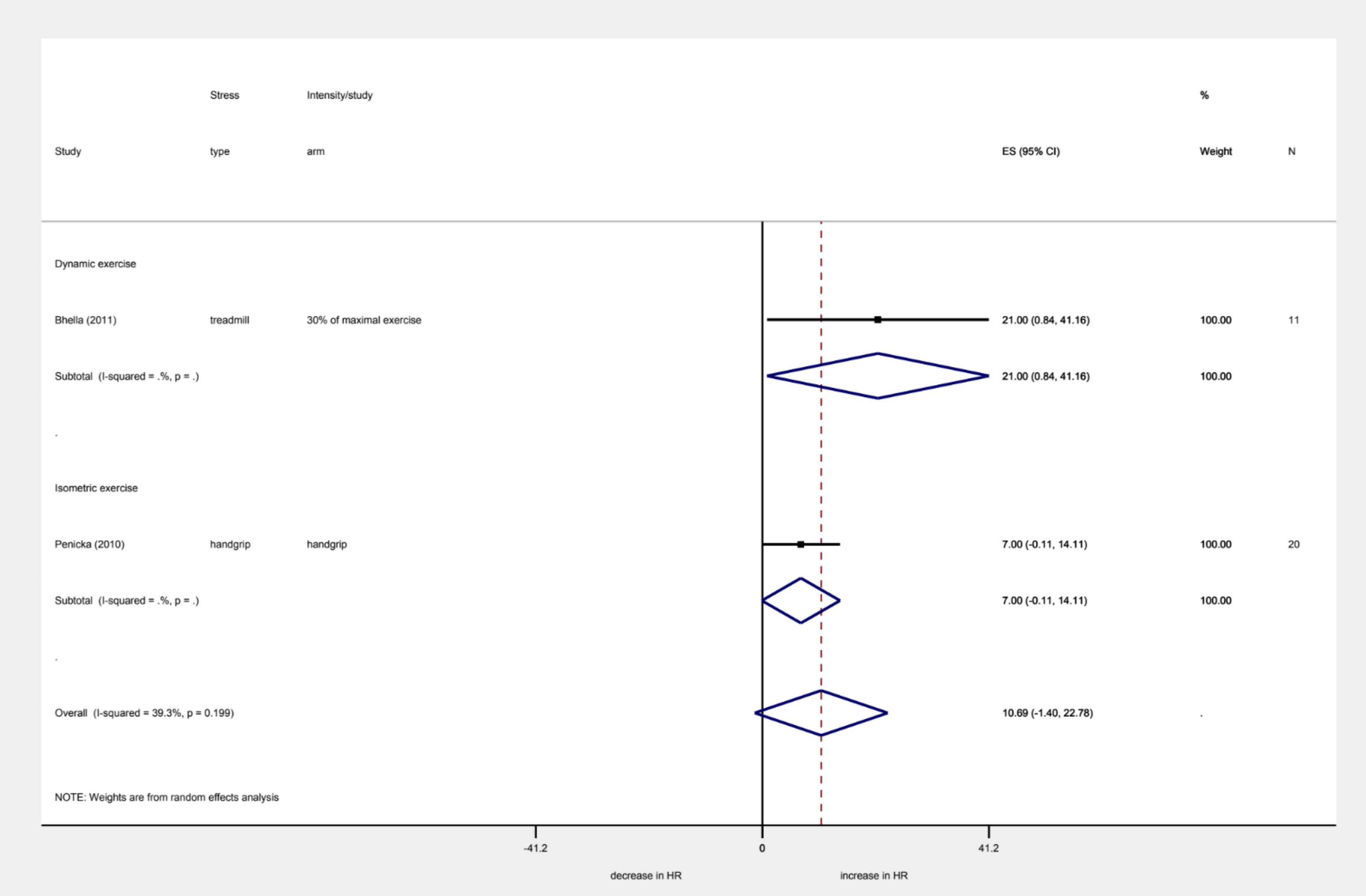

Figure S9. HR Changes [%] in heart failure patients with preserved ejection fraction for light intensity stress.

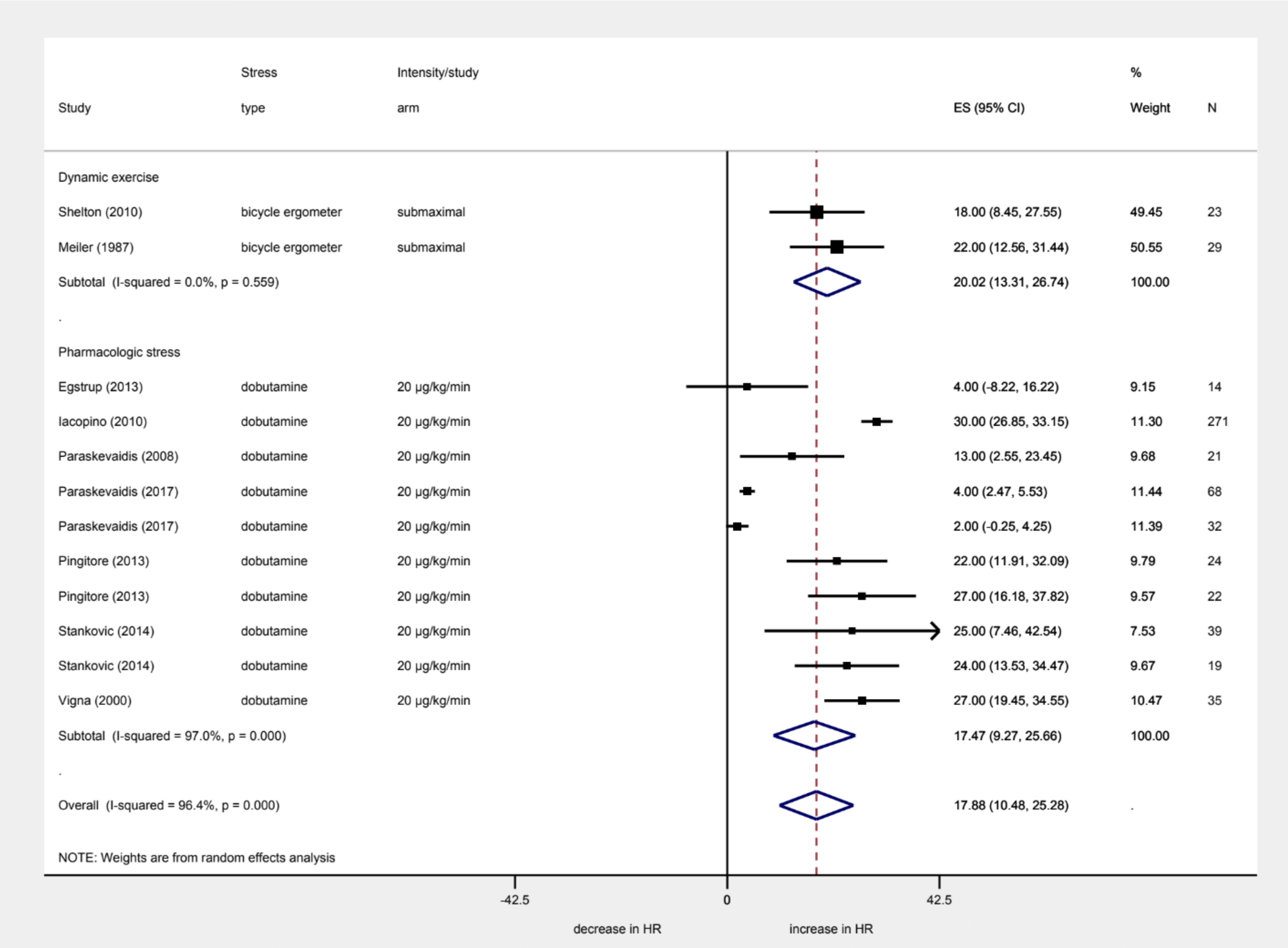

Figure S10. HR Changes [%] in heart failure patients with reduced ejection fraction for moderate intensity stress.

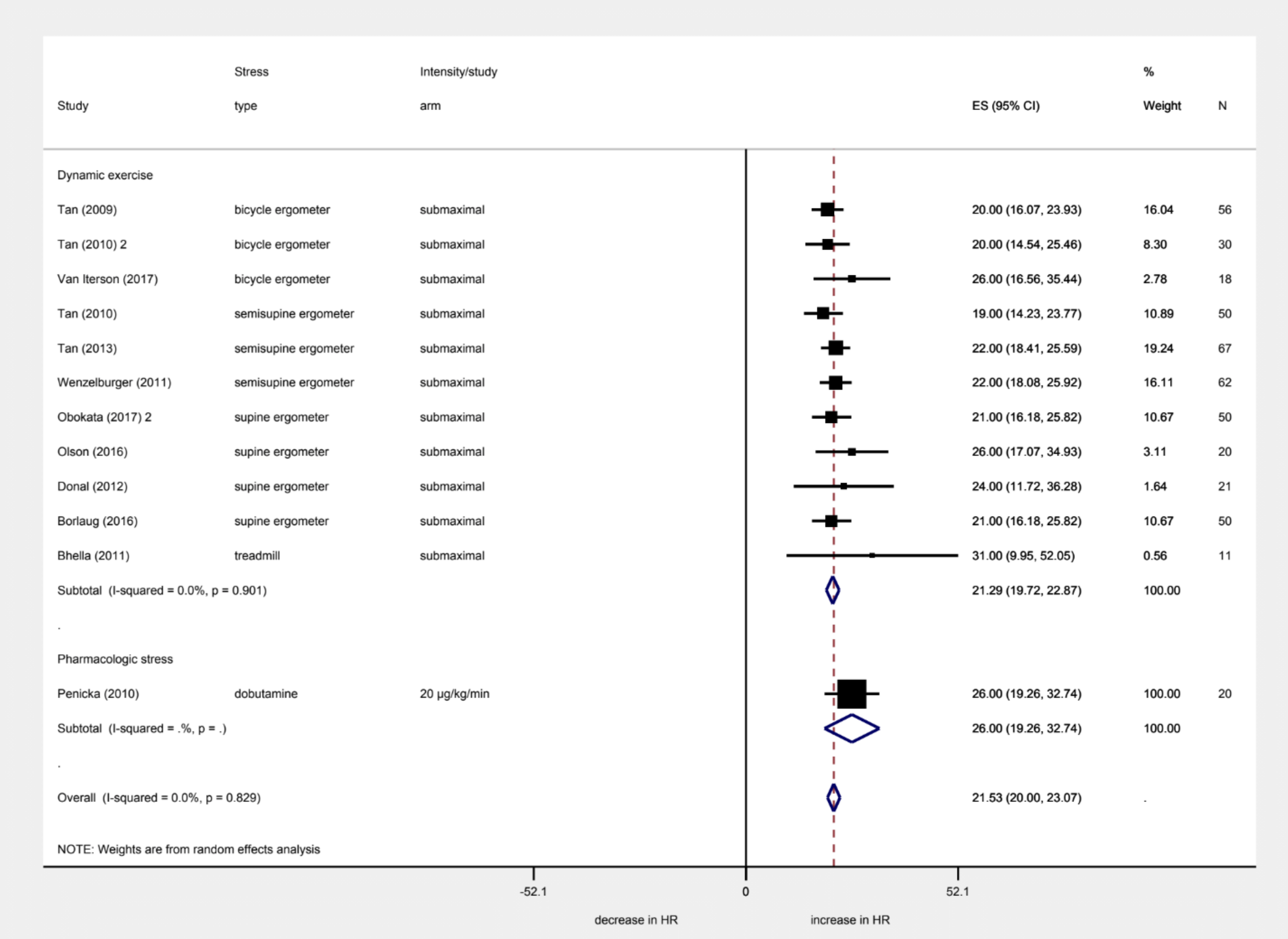

Figure S11. HR Changes [%] in heart failure patients with preserved ejection fraction for moderate intensity stress.

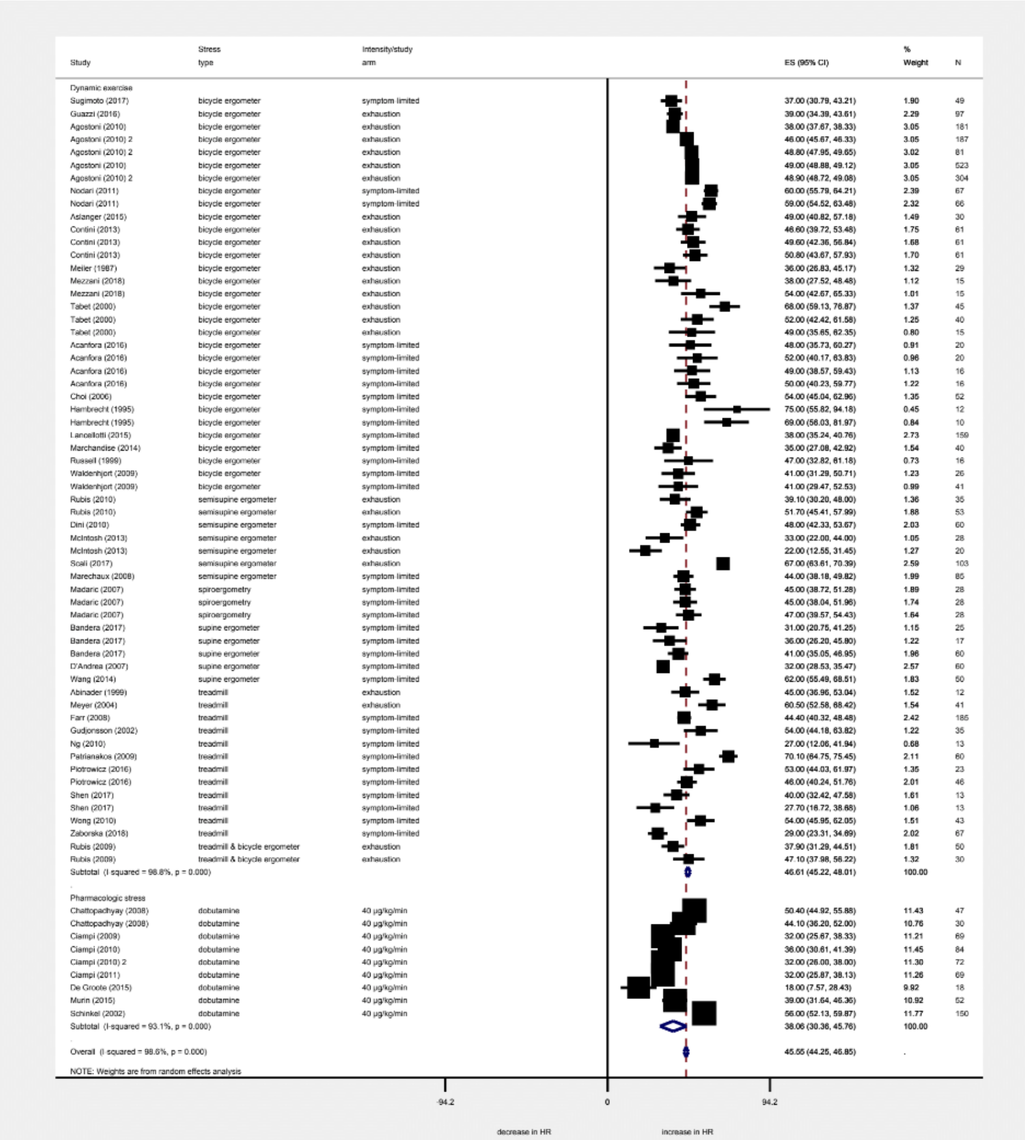

Figure S12. HR Changes [%] in heart failure patients with reduced ejection fraction for high intensity stress.

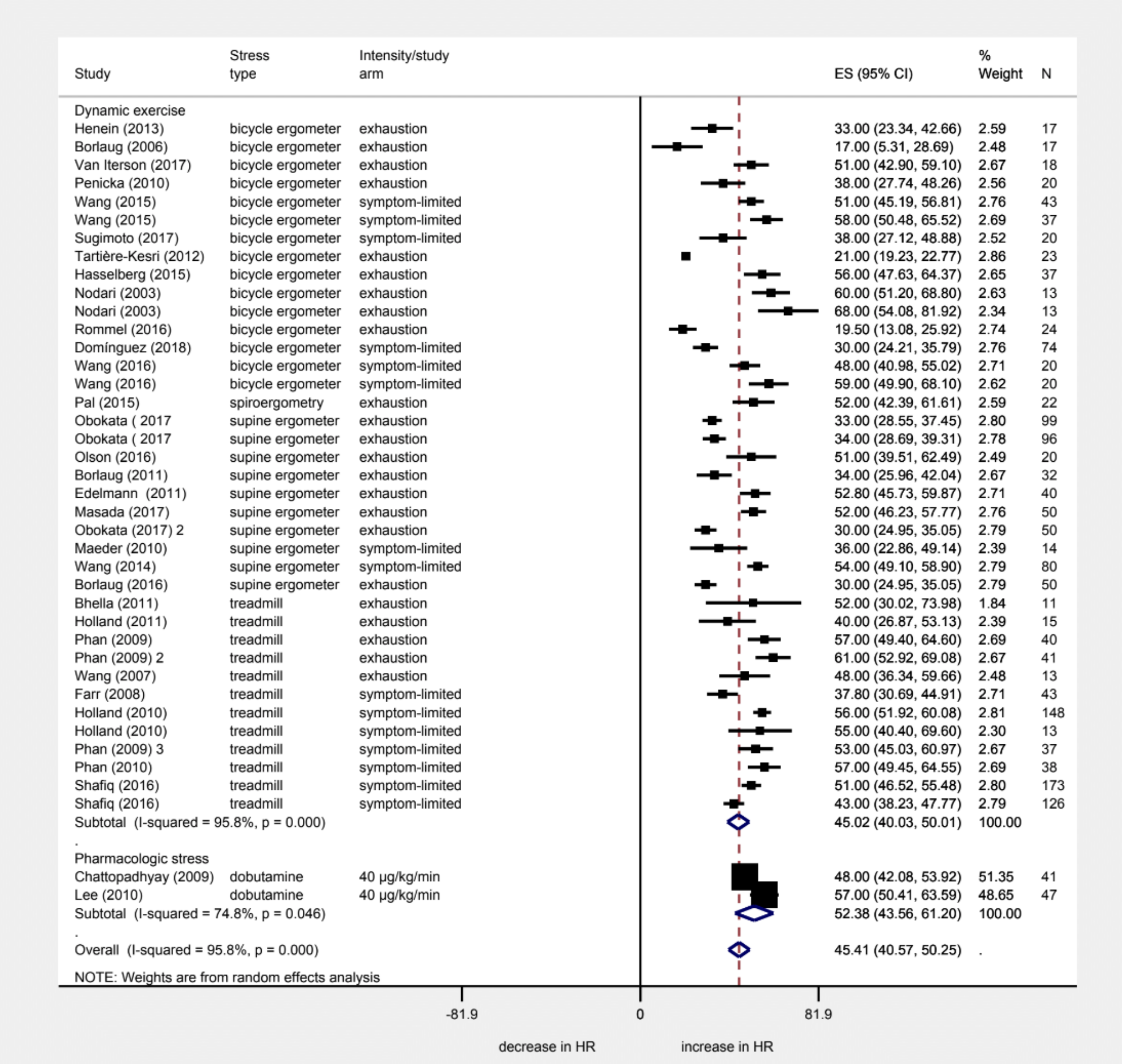

Figure S13. HR Changes [%] in heart failure patients with preserved ejection fraction for high intensity stress.

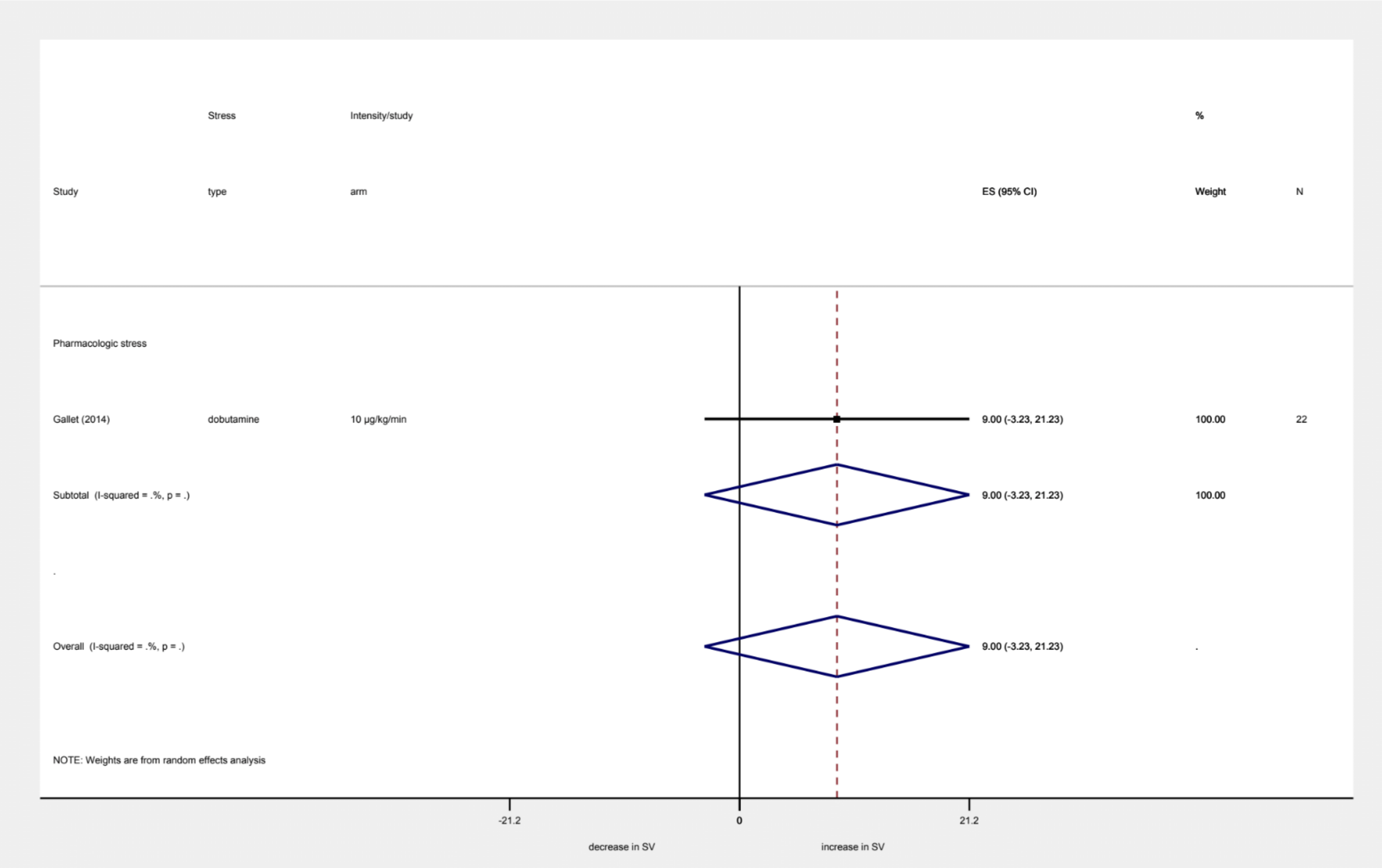

Figure S14. SV Changes [ml] in heart failure patients with reduced ejection fraction for light intensity stress.

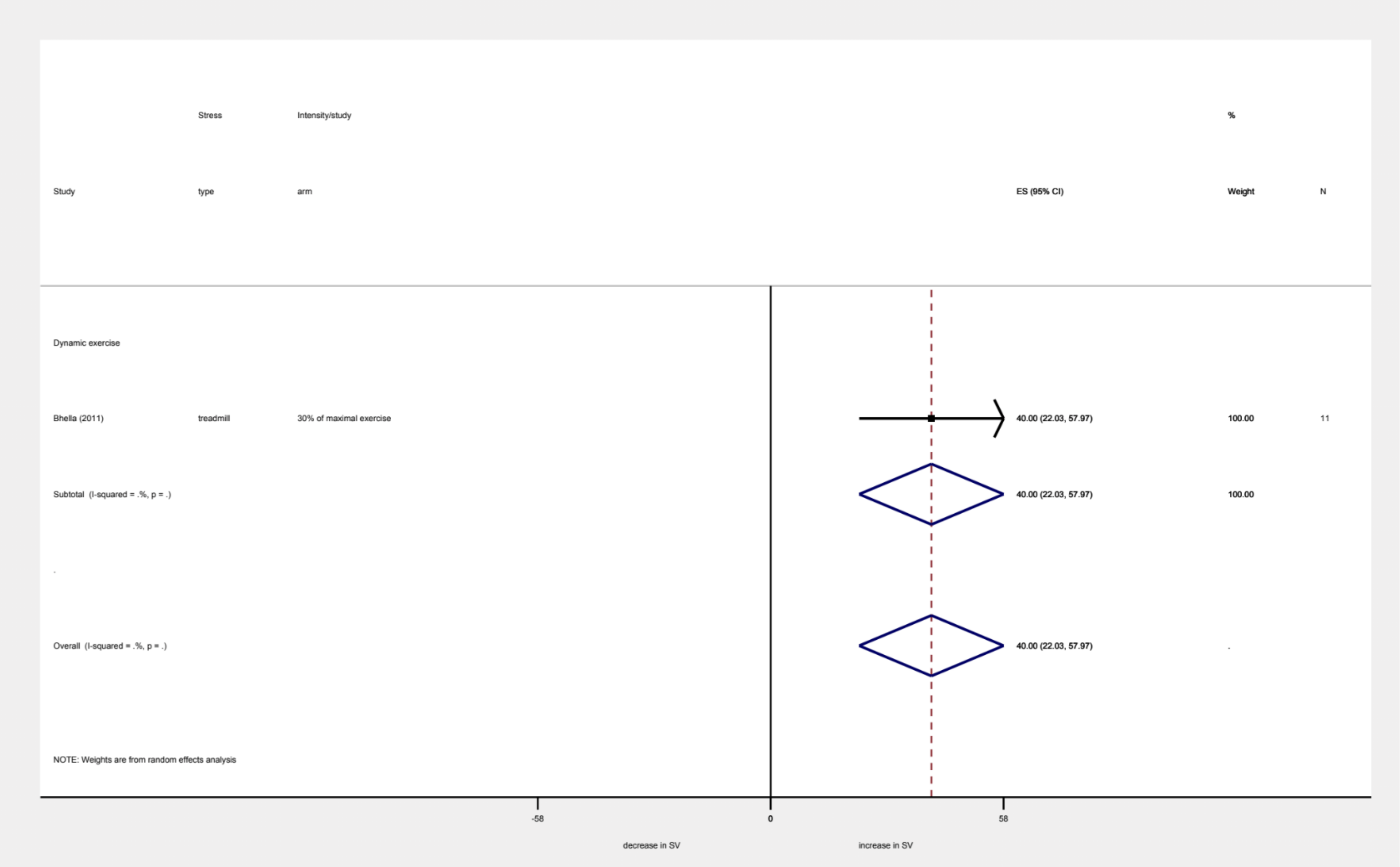

FigureS15. SV Changes [ml] in heart failure patients with preserved ejection fraction for light intensity stress.

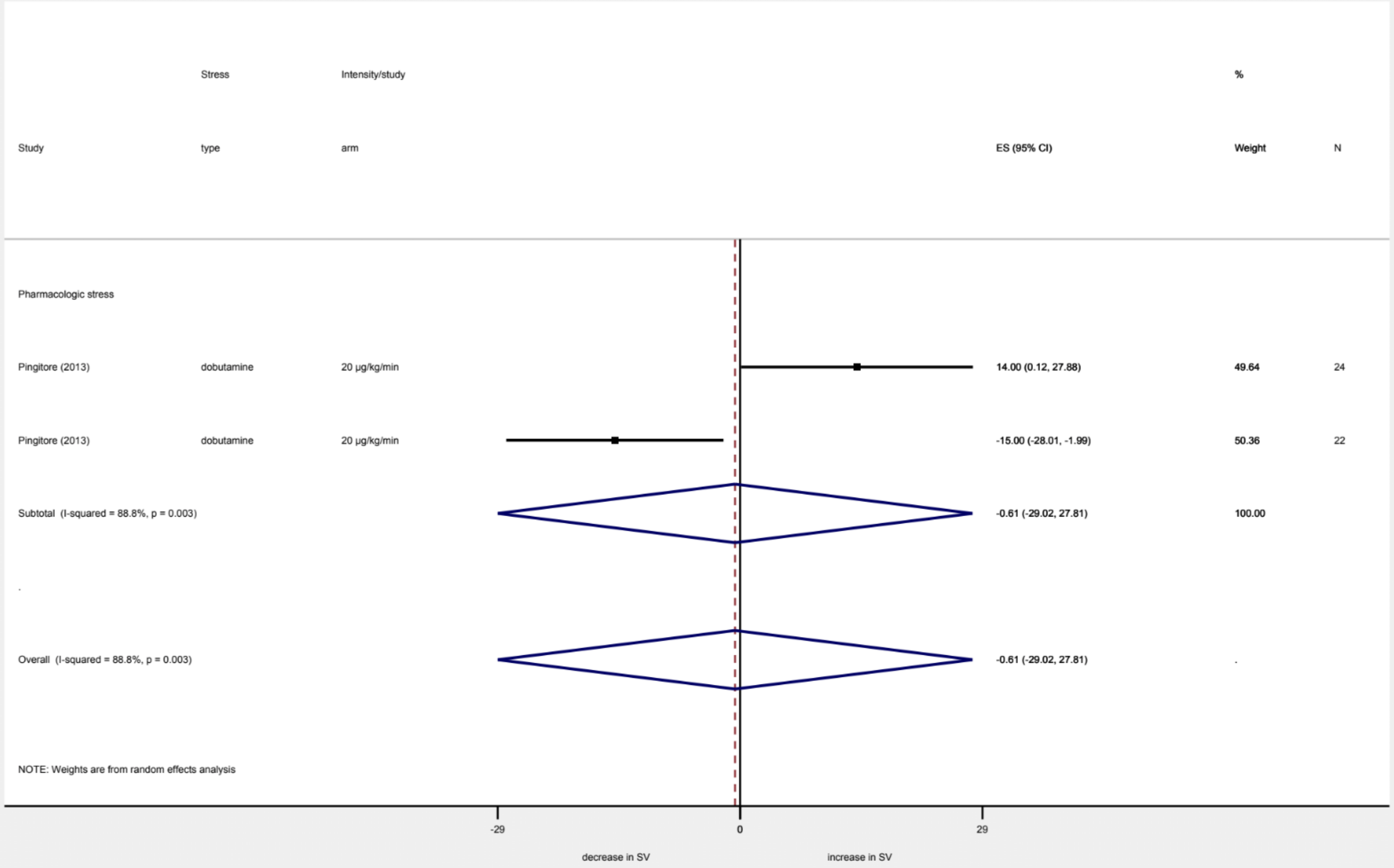

Figure S16. SV Changes [ml] in heart failure patients with reduced ejection fraction for moderate intensity stress.

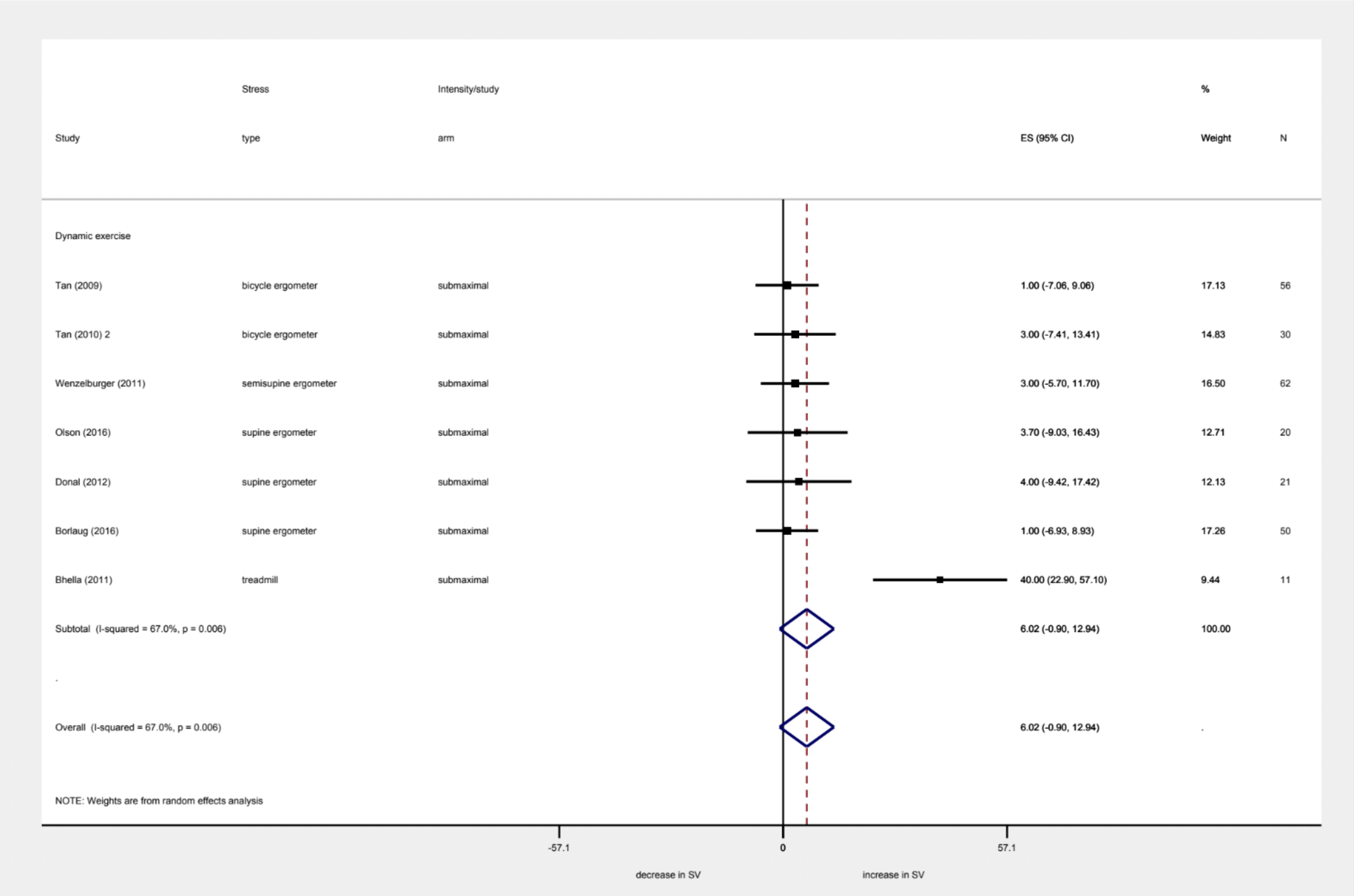

Figure S17. SV Changes [ml] in heart failure patients with preserved ejection fraction for moderate intensity stress.

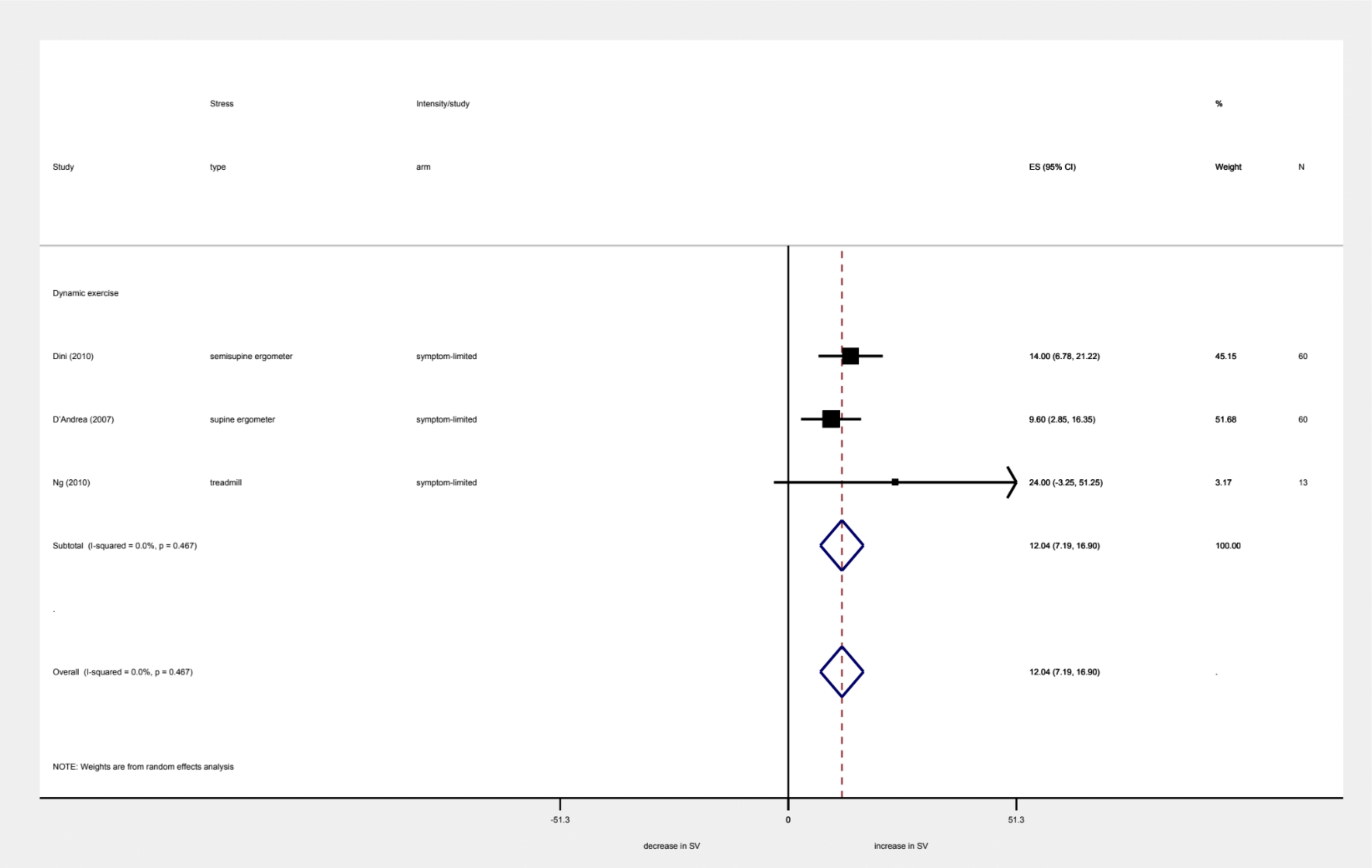

Figure S18. SV Changes [ml] in heart failure patients with reduced ejection fraction for high intensity stress.

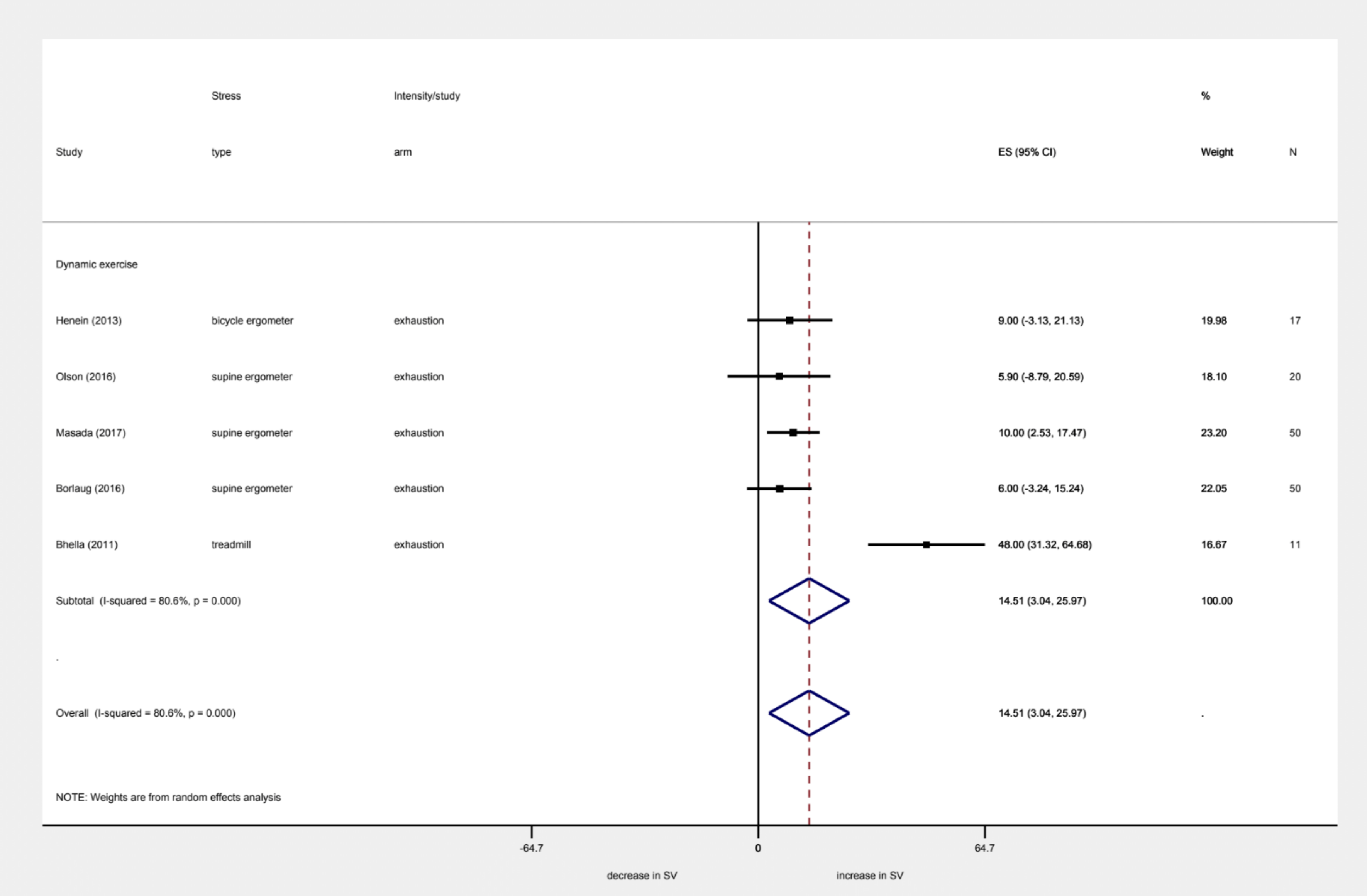

Figure S19. SV Changes [ml] in heart failure patients with preserved ejection fraction for high intensity stress.

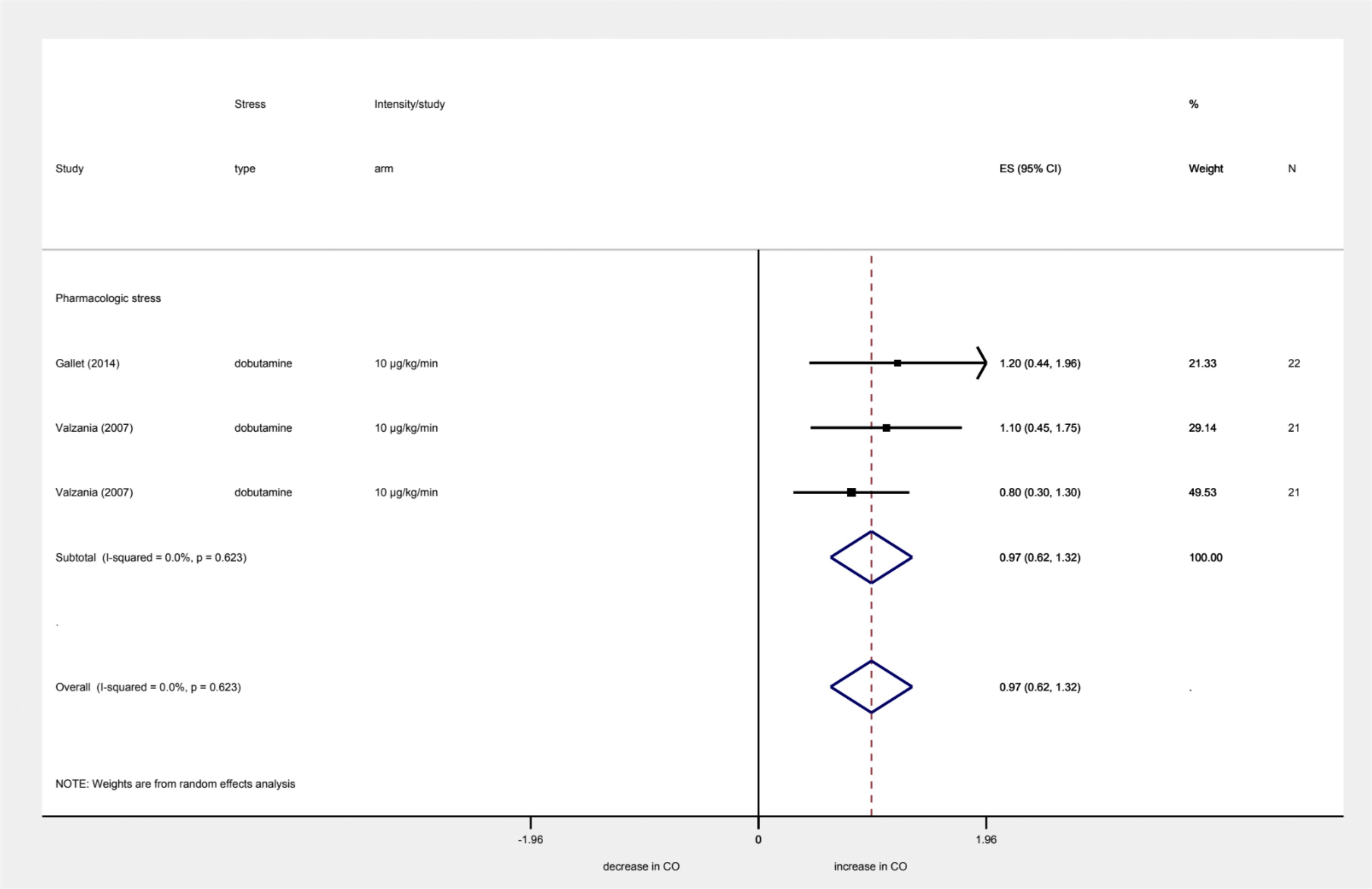

Figure S20. CO Changes [l/min] in heart failure patients with reduced ejection fraction for light intensity stress.

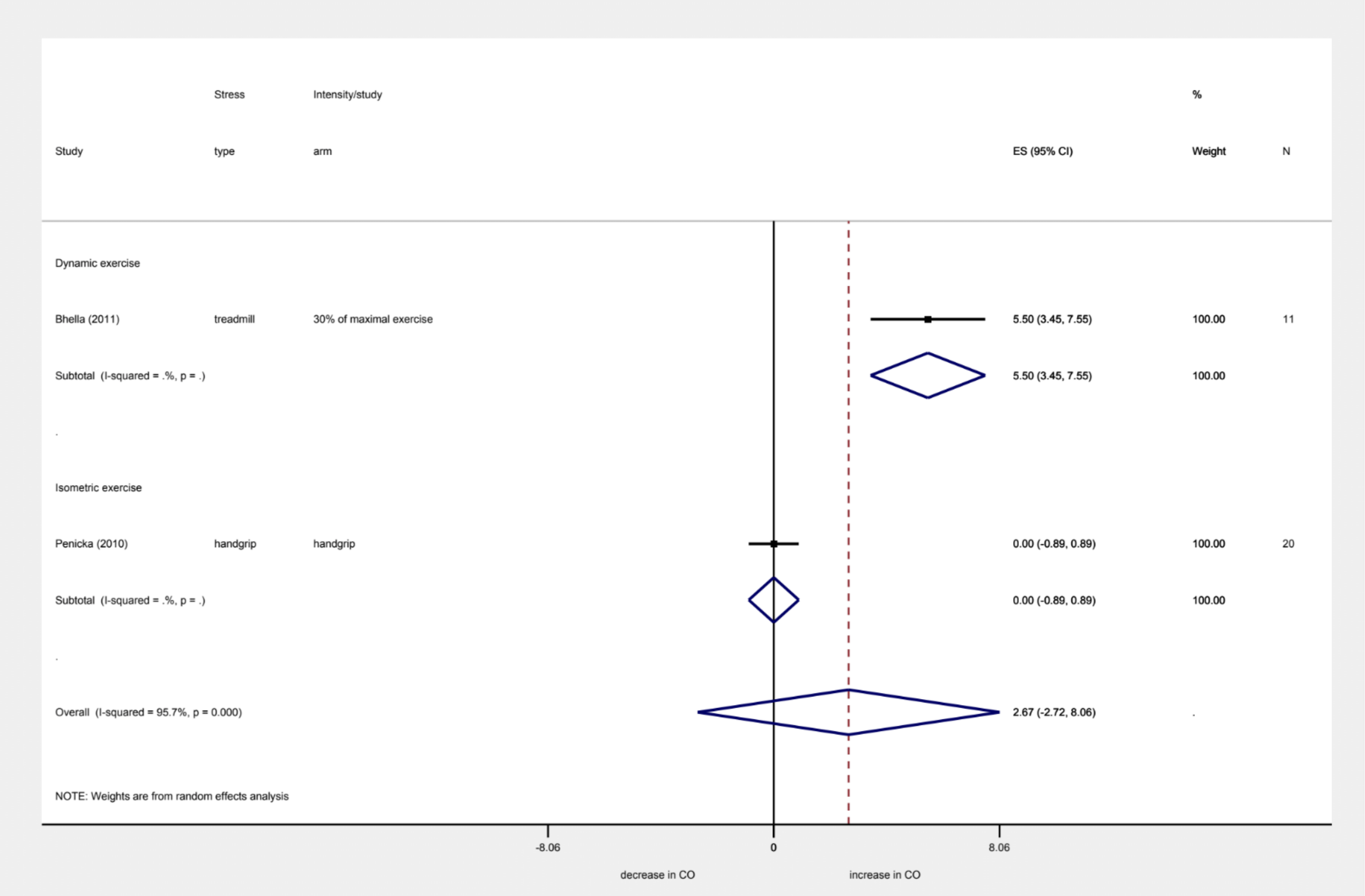

Figure S21. CO Changes [l/min] in heart failure patients with preserved ejection fraction for light intensity stress.

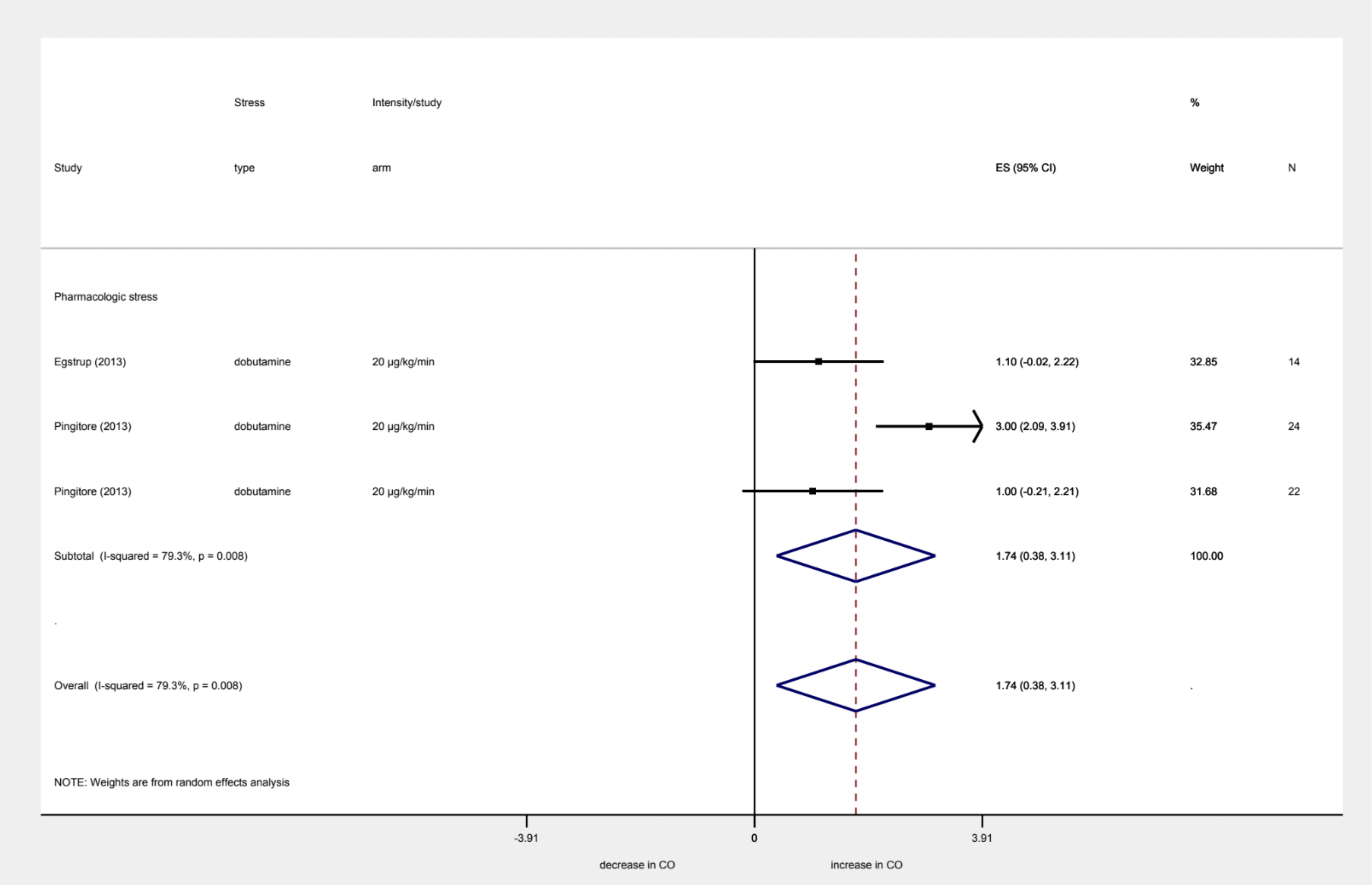

Figure S22. CO Changes [l/min] in heart failure patients with reduced ejection fraction for moderate intensity stress.

frontiers

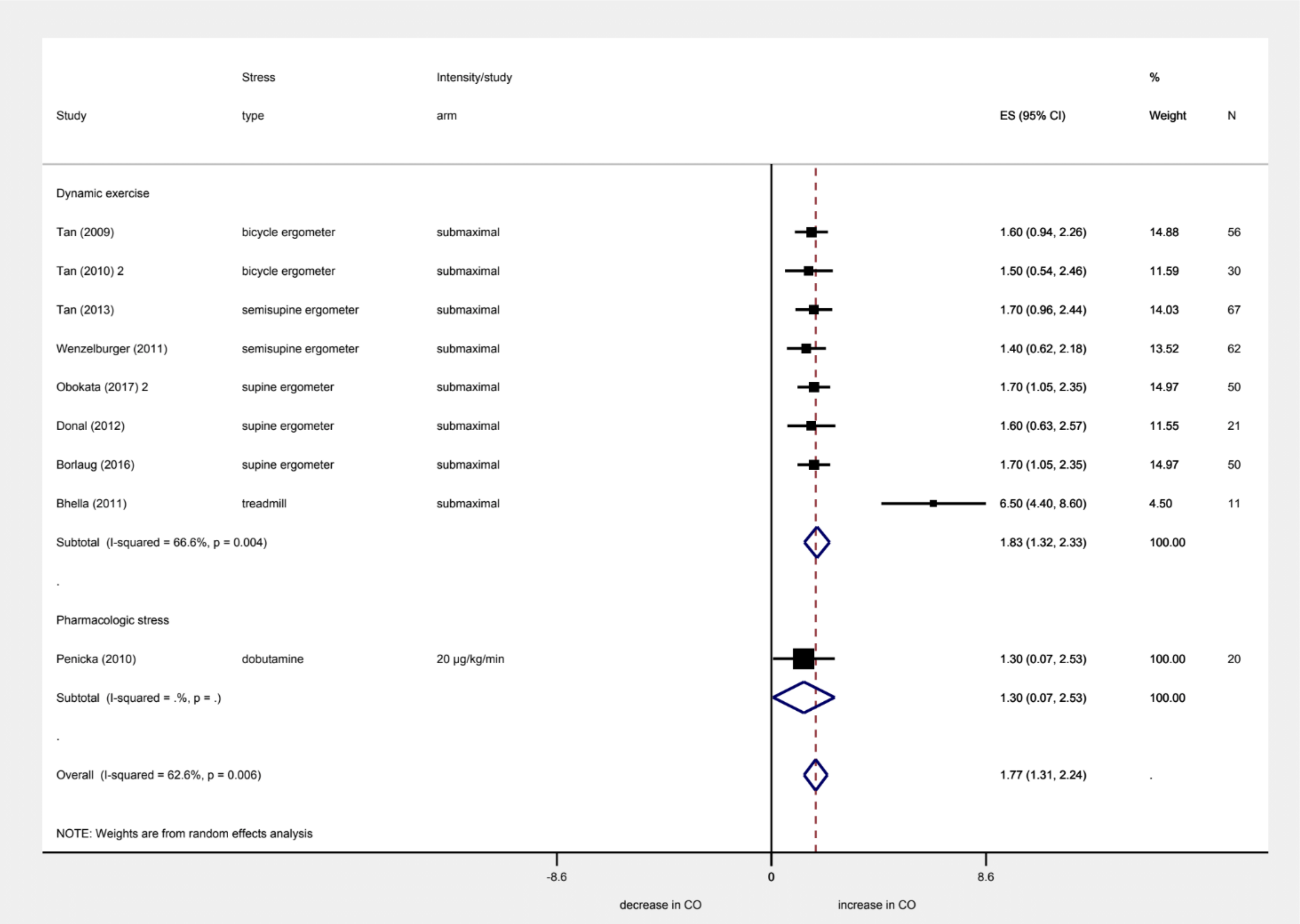

Figure S23. CO Changes [l/min] in heart failure patients with preserved ejection fraction for moderate intensity stress.

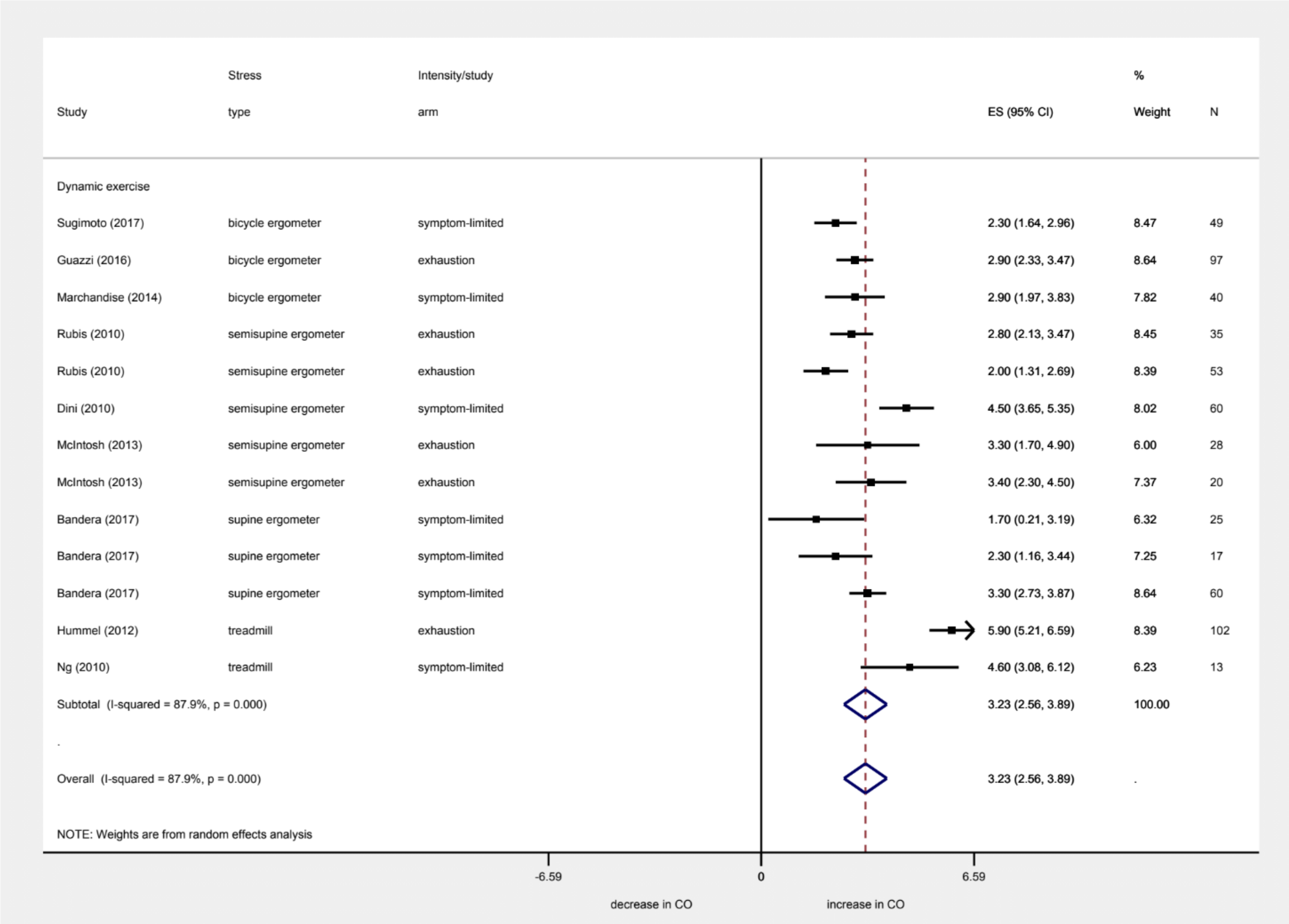

Figure S24. CO Changes [l/min] in heart failure patients with reduced ejection fraction for high intensity stress.

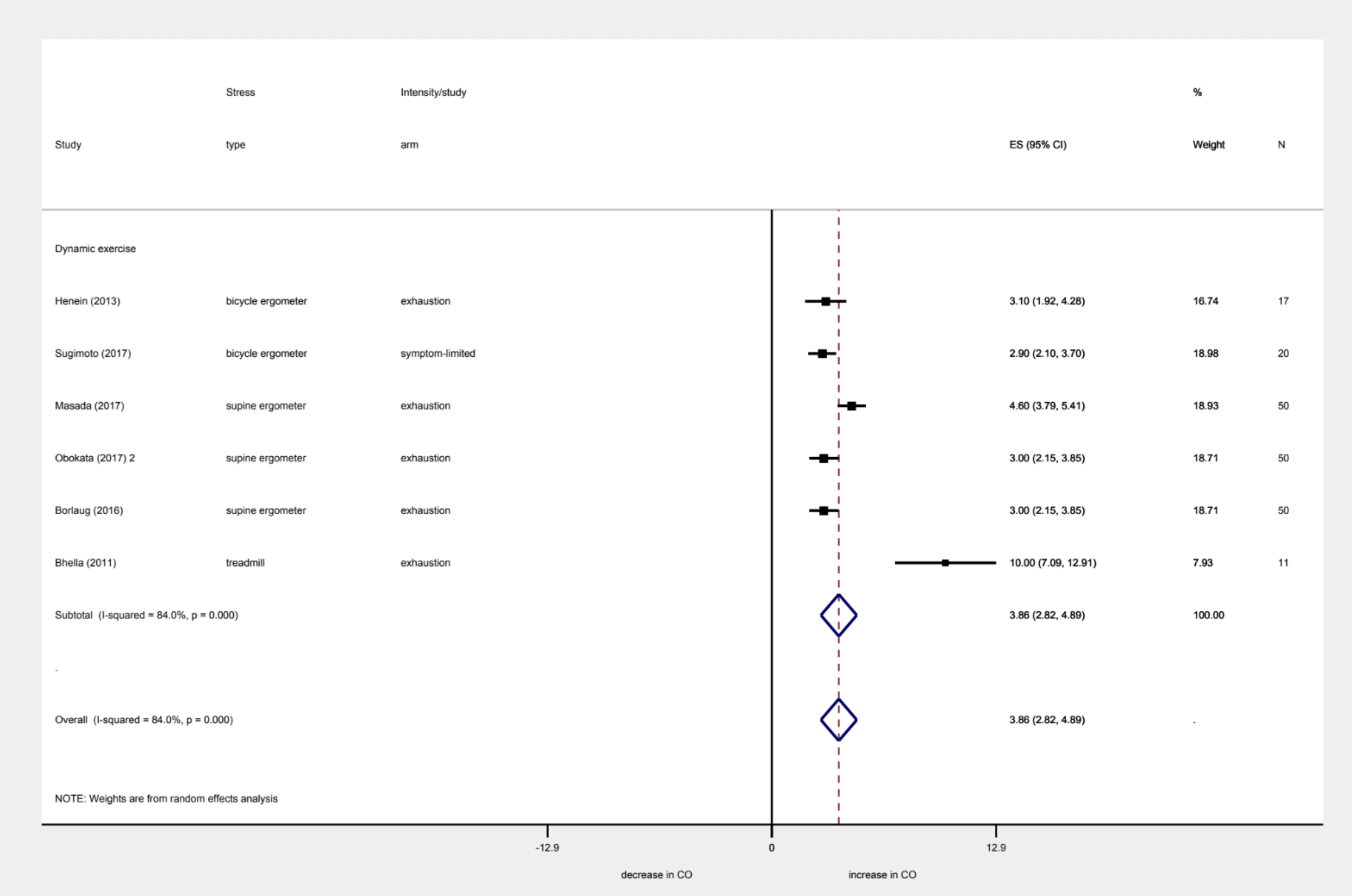

Figure S25. CO Changes [l/min] in heart failure patients with preserved ejection fraction for high intensity stress.

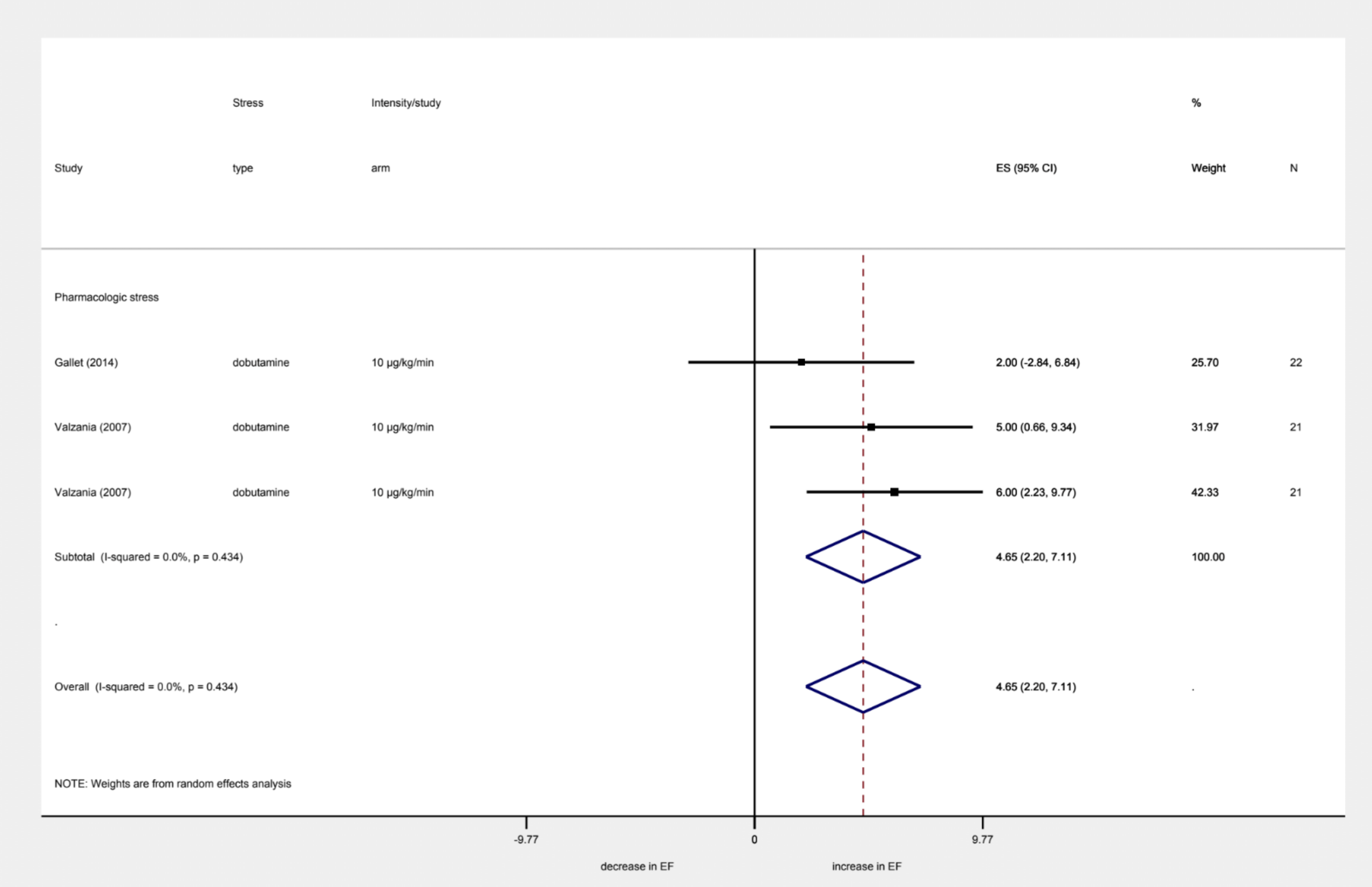

Figure S26. EF Changes [%] in heart failure patients with reduced ejection fraction for light intensity stress.

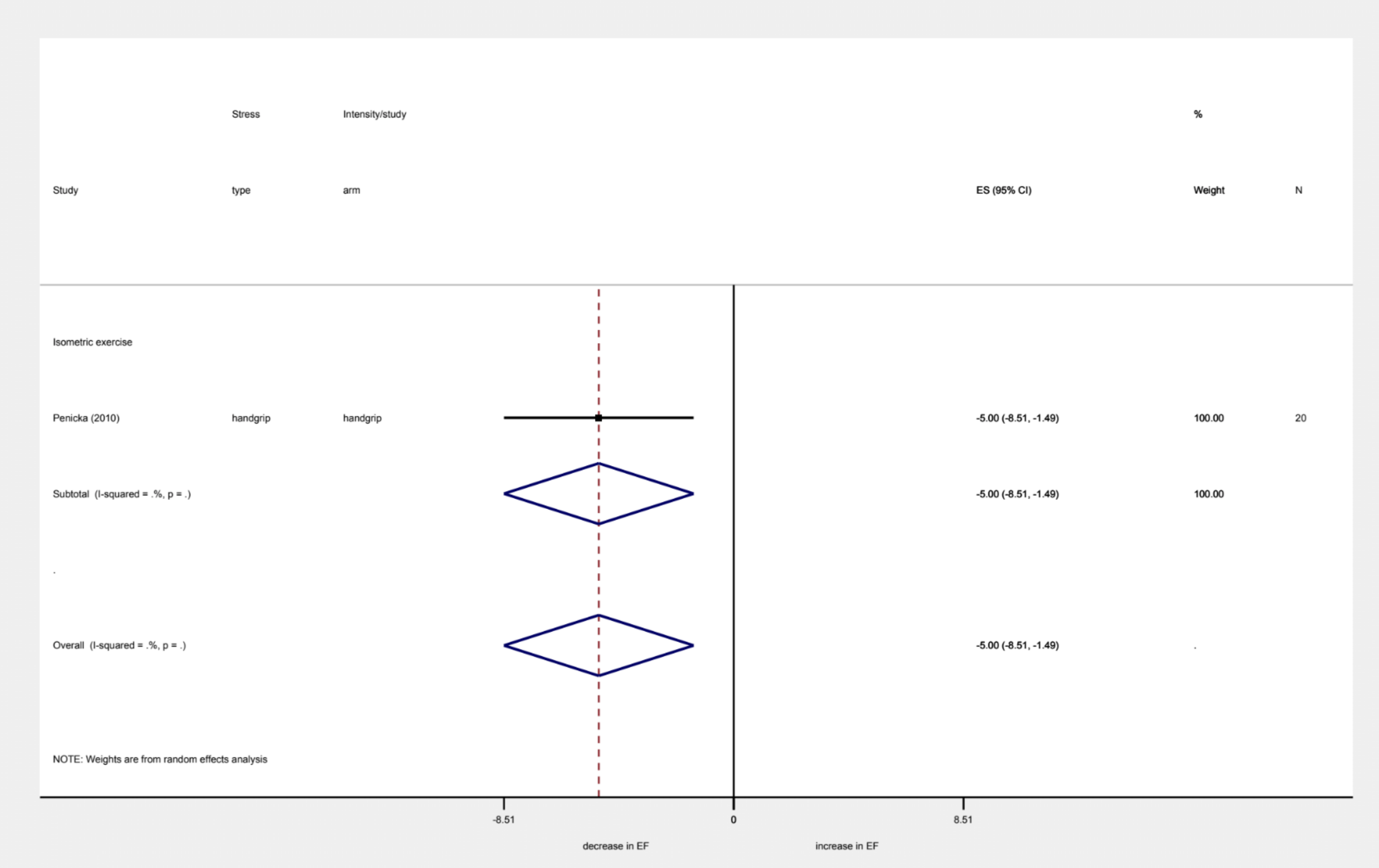

Figure S27. EF Changes [%] in heart failure patients with preserved ejection fraction for light intensity stress.

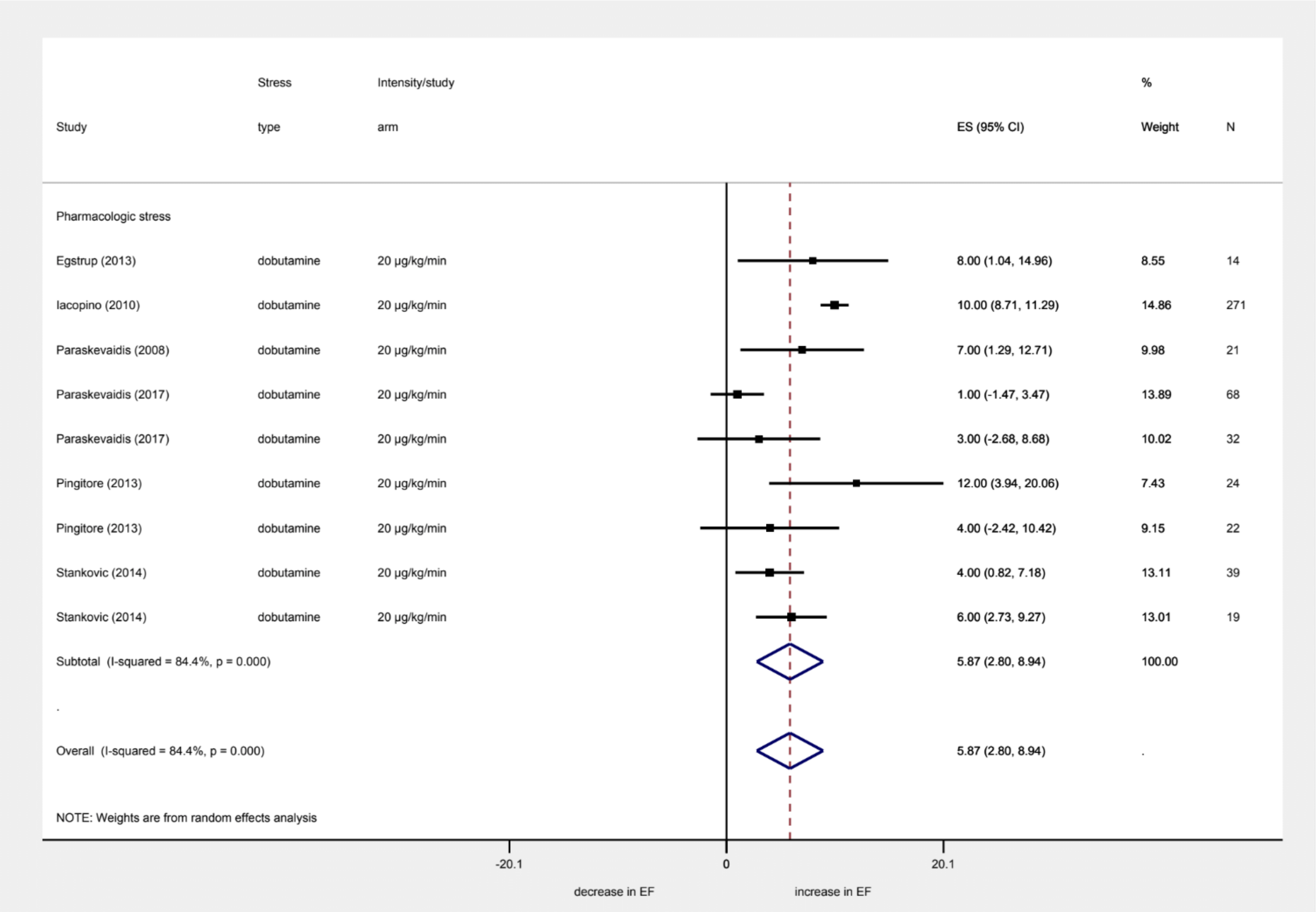

Figure S28. EF Changes [%] in heart failure patients with reduced ejection fraction for moderate intensity stress.

frontiers

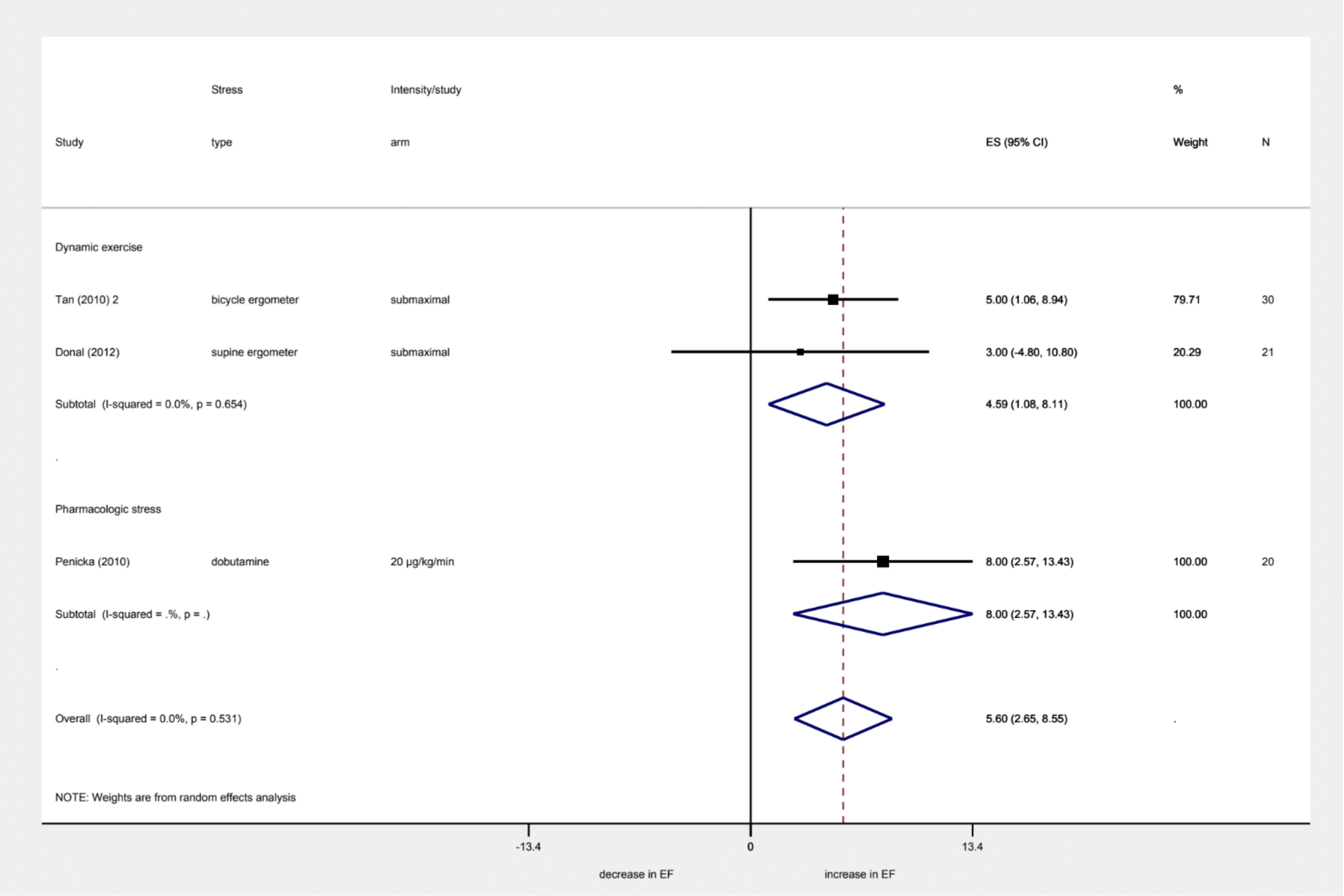

Figure S29. EF Changes [%] in heart failure patients with preserved ejection fraction for moderate intensity stress.

frontiers

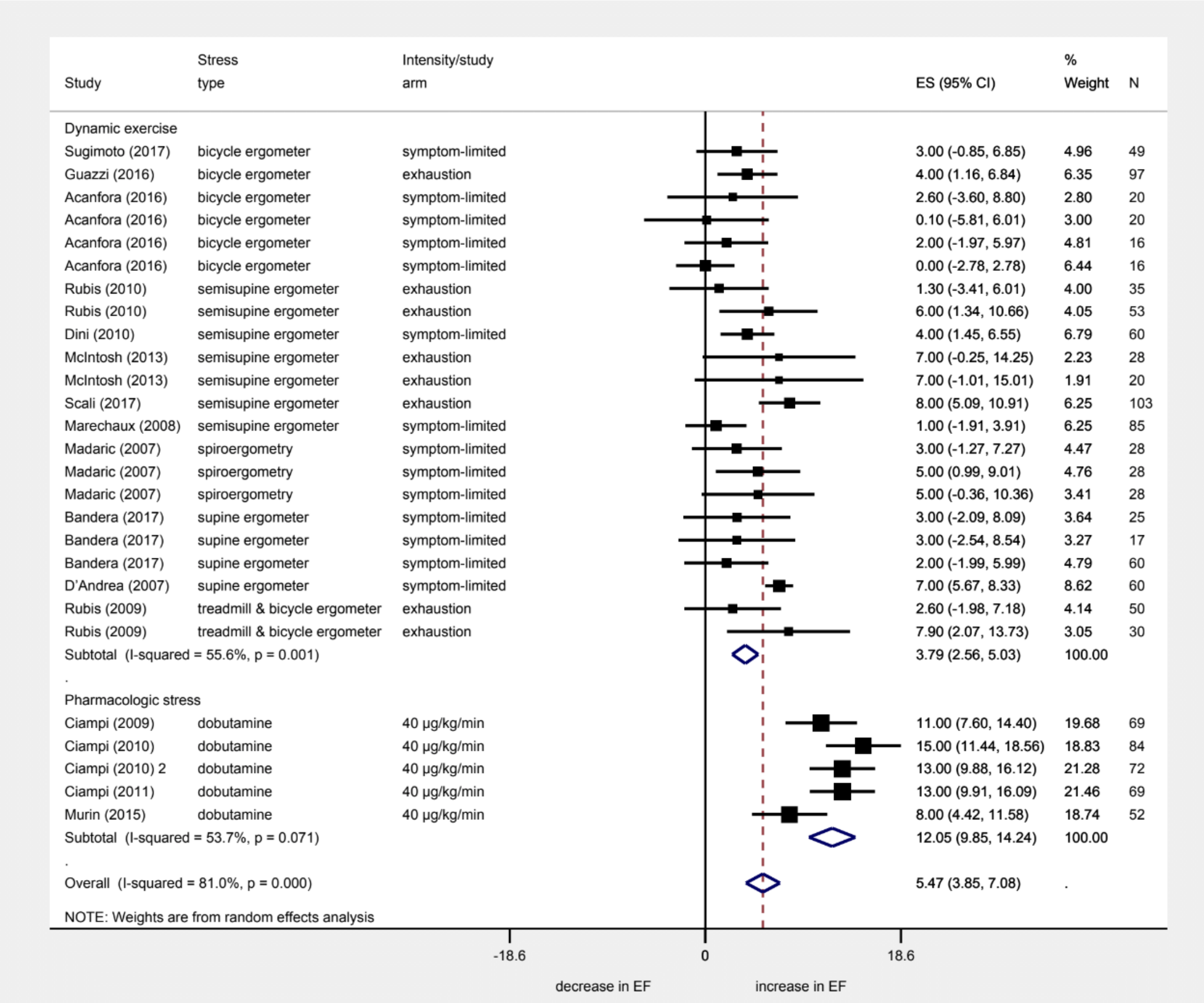

Figure S30. EF Changes [%] in heart failure patients with reduced ejection fraction for high intensity stress.

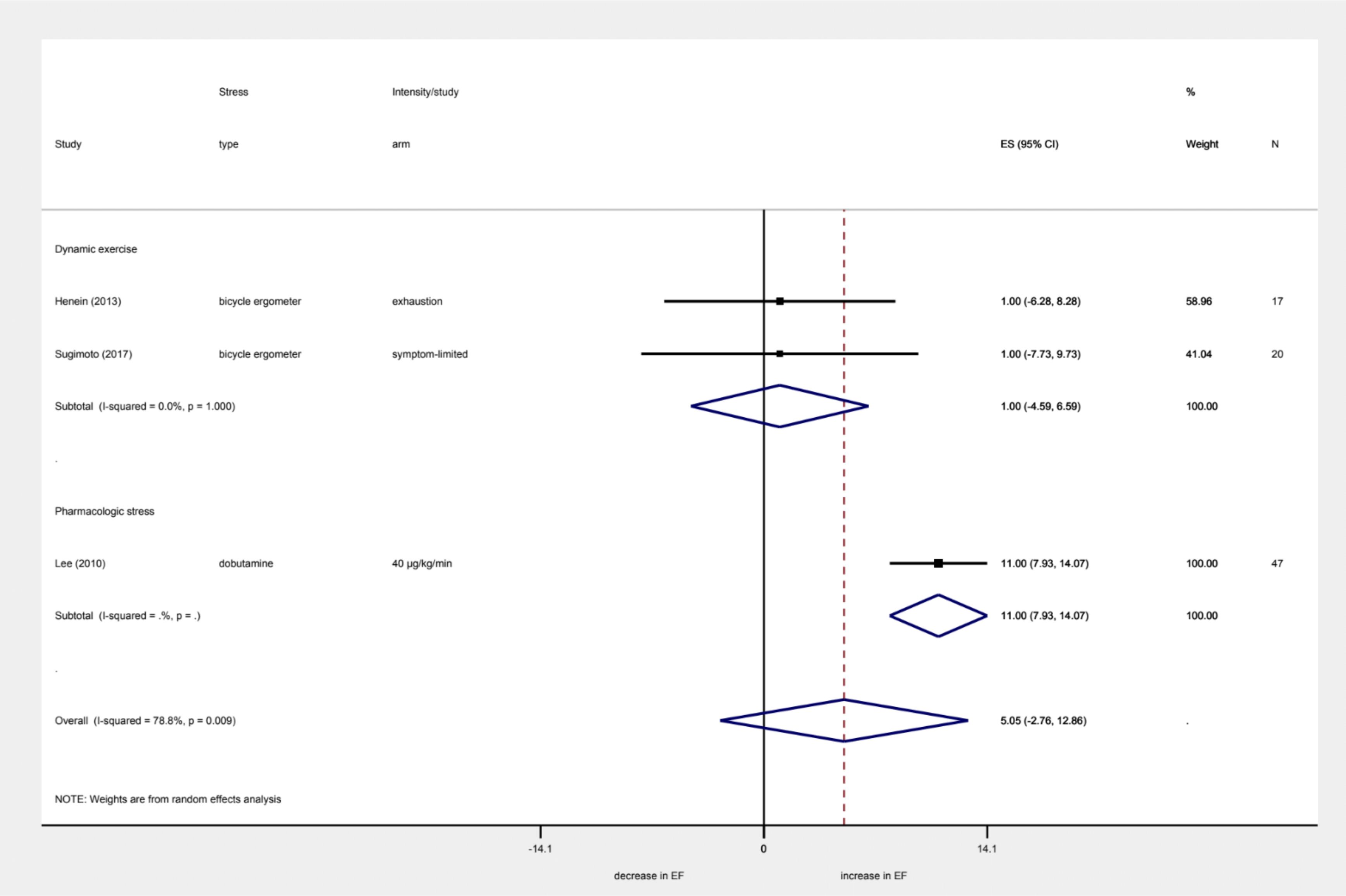

Figure S31. EF Changes [%] in heart failure patients with preserved ejection fraction for high intensity stress.
